# Identification and Molecular Characterization of the CAMTA Gene Family in Solanaceae with a Focus on the Expression Analysis of Eggplant Genes under Cold Stress

**DOI:** 10.3390/ijms25042064

**Published:** 2024-02-08

**Authors:** Peng Cai, Yanhong Lan, Fangyi Gong, Chun Li, Feng Xia, Yifan Li, Chao Fang

**Affiliations:** 1 Horticulture Research Institute, Sichuan Academy of Agricultural Sciences, Chengdu 610066, China; 2 Vegetable Germplasm Innovation and Variety Improvement Key Laboratory of Sichuan Province, Chengdu 610066, China

**Keywords:** CAMTA, eggplant, evolution, abiotic stress, cold

## Abstract

Calmodulin-binding transcription activator (CAMTA) is an important calmodulin-binding protein with a conserved structure in eukaryotes which is widely involved in plant stress response, growth and development, hormone signal transduction, and other biological processes. Although *CAMTA* genes have been identified and characterized in many plant species, a systematic and comprehensive analysis of *CAMTA* genes in the Solanaceae genome is performed for the first time in this study. A total of 28 *CAMTA* genes were identified using bioinformatics tools, and the biochemical/physicochemical properties of these proteins were investigated. *CAMTA* genes were categorized into three major groups according to phylogenetic analysis. Tissue-expression profiles indicated divergent spatiotemporal expression patterns of *SmCAMTAs*. Furthermore, transcriptome analysis of *SmCAMTA* genes showed that exposure to cold induced differential expression of many eggplant *CAMTA* genes. Yeast two-hybrid and bimolecular fluorescent complementary assays suggested an interaction between SmCAMTA2 and SmERF1, promoting the transcription of the cold key factor *SmCBF2*, which may be an important mechanism for plant cold resistance. In summary, our results provide essential information for further functional research on Solanaceae family genes, and possibly other plant families, in the determination of the development of plants.

## 1. Introduction

The Solanaceae family, comprising approximately 3000 plant species, plays a crucial role in the realm of vegetable crops and holds economic significance surpassed only by cereals and leguminous plants. Notable members of this family include potatoes, tomatoes, chili peppers, eggplants, goji berries, tobacco, and Bidong eggplants. Fruits from the Solanaceae family boast a wealth of nutrients, encompassing proteins, fats, carbohydrates, vitamins, antioxidant polyphenols, and essential trace elements like calcium, phosphorus, and iron. This nutritional richness makes them highly sought after as a popular food source and a vital vegetable crop [1,2]. The global cultivation of Solanaceae crops is extensive, particularly in Asia, with China leading as the largest producer. In 2020, China contributed to 68% of the global production. However, the cultivation of Solanaceae crops faces challenges, and their yield and quality are often compromised by cold damage [3,4]. Therefore, investigating the mechanisms through which crops respond to cold damage has become an urgent necessity. With continuous progress in genomics and bioinformatics, the isolation and cloning of and functional research on the cold regulatory factor CAMTA (Calmodulin-binding transcription activator) have gained considerable attention.

CAMTA represents an essential class of structurally conserved calmodulin-binding proteins which are widely present in eukaryotes [5,6,7]. The CAMTA protein family’s secondary structures consistently feature a conserved functional domain module. From the N-end, these structures comprise a CG-1 DNA binding domain, a transcription factor immunoglobulin (TIG) domain, an ankyrin repeat (ANK) domain, a Ca^2+^- dependent calmodulin binding domain (CaMBD), and a tandem repeat IQ motif (IQXXXRGXXX) functional domains [8,9]. Furthermore, evidence suggests that the TIG domain associates with non-specific DNA contact with transcription factors, the ANK repeat is linked to protein interactions, and the IQ motif can bind to calmodulin (CaM) or calmodulin-like (CML) proteins without relying on Ca^2+^ [10,11]. However, recent research has unveiled that the CG-1 domain of Arabidopsis CAMTA3 imparts temperature dependence to the ability of CAMTA3 TAD (newly identified transcriptional activation domain) to induce gene expression [12].

The CAMTA family has been identified in various plant species, including *Arabidopsis thaliana* [13], *Triticum aestivum* [14], *Musa nana* [15], *Oryza sativa* [16], *Brassica napus* [17], and *Camellia sinensis* [18]. CAMTA is a relatively small, plant-specific family usually consisting of fewer than 10 members, although there were 24 members identified in *B. napus*. Plant CAMTA genes play a crucial role in regulating various processes of plant growth and development, such as those related to flowers, leaves, and roots. Additionally, they are involved in responding to environmental stresses such as drought, salt, cold, and injury, as well as regulating responses to hormone signals like ethylene, abscisic acid (ABA), auxin, salicylic acid (SA), and jasmonic acid (JA) [19,20]. Under low-temperature conditions, *Arabidopsis* CAMTAs respond to rapid temperature decrease by activating downstream DREB1 expression [21]. *Arabidopsis* AtCAMTA3, for instance, interacts with 10 transcription factors, including AtCBF2, to positively regulate the expression of the cold stress gene *AtCBF2* [22]. Both *AtCAMTA1* and *AtCAMTA3* exhibit significantly reduced tolerance to freezing [23,24]. AtCAMTA1 and AtCAMTA2 synergistically interact with AtCAMTA3 at low temperature (4 °C), inducing high transcriptional levels of *AtCBF1*, *AtCBF2*, and *AtCBF3* within 2 h. This interaction promotes the biosynthesis of salicylic acid, enhancing plant cold resistance [23]. Two homozygous T-DNA insertion mutants (camta3-1, camta3-2) of *AtCAMTA3* in *Arabidopsis* show increased spontaneous damage [25]. Overexpression of *GmCAMTA12* in *Arabidopsis* and soybean reveals that they function in the regulation of soybean root development and drought stress [26]. *ZmCAMTA4a*, *ZmCAMTA7a*, and *ZmCAMTA7b* are significantly upregulated in the buds after cold stress treatment in maize, with only *ZmCAMTA4a* induced by cold stress in the roots [27]. Doherty et al. found that tomato SlCAMTA3 can bind to the conserved motif CCGCGT of SlCBF2, thereby positively regulating *SlCBF2*. In addition, the other two low-temperature-induced genes, *SlCBF1* and *ZAT12* (encoding a zinc finger protein), may also be directly regulated by *SlCAMTA3* [28]. However, their induction is inhibited by low temperature in the CAMTA1/sr2-CAMTA3/sr1 double mutant, suggesting joint regulation by CAMTA3/SR1 and CAMTA1/SR2 in plant responses to low-temperature stress [29]. Kim et al. observed that among the three functionally redundant CAMTA genes, only CAMTA3 expression was induced by low temperature. This may explain why the CAMTA3 mutant is sensitive to temperature drops when a single gene is missing [23,30]. Researchers also found that CAMT1-3 synergistically promotes the rapid response of CBF1-3 to low temperatures [23,31]. Despite the presence of many stress-related elements in the CAMTA gene promoter region, the upstream regulatory factors governing CAMTA gene expression remain unclear. Additionally, downstream genes regulated by CAMTA are currently limited in number. It is essential to identify target genes regulated by CAMTA on a genome-wide scale, providing a foundation for a comprehensive exploration of CAMTA functions and the analysis of its regulatory role in plant stress response, growth, and development processes.

Eggplant, particularly the purple variety, boasts a higher vitamin content compared to other members of the Solanaceae family, making it a popular and economically significant vegetable crop. However, eggplant is highly susceptible to low temperatures during the planting process, and enhancing its cold resistance has become a key research focus. In recent years, technological advancements, including modern molecular biology methods, high-throughput sequencing, and genetic engineering technologies, have led to continuous improvements in eggplant genome data. This progress has facilitated the extensive screening of low-temperature-tolerant germplasm and the identification of related gene expressions, garnering widespread attention [32,33]. These advancements offer a more in-depth understanding of the cold response mechanism and molecular pathways in eggplant. Against this backdrop, it is crucial to explore additional genes that respond to low temperatures in the eggplant genome. A comprehensive understanding and analysis of the regulatory mechanism of low-temperature stress in eggplant can be achieved through the use of modern technologies. This study conducts a genome-wide identification of CAMTA proteins from the Solanaceae family and examines the structure of CAMTA members at the whole-genome level. The evolutionary relationships among different members of this gene family are investigated, along with their expression patterns in eggplant under cold conditions. In summary, the results of this study aim to provide valuable information for further investigations into the functional and regulatory mechanisms of CAMTA, serving as core components in the Solanaceae family.

## 2. Results

### 2.1. Identification and Evolution Analysis of CAMTA Family Members

In this study, a total of 28 CAMTA members were identified from the Solanaceae Genomics database (https://solgenomics.net/, accessed on 14 October 2023) [34]. The number of CAMTA genes ranged from three to seven in different species. The basic physical and chemical properties of the CAMTA family members were analyzed (Table 1). The results showed that the ORF lengths of CAMTAs varied from 2.355 (*CaCAMTA4*) kb to 3.309 (*SmCAMTA2*) kb; amino acid lengths ranged from 785 (CaCAMTA4) to 1103 (SmCAMTA2) aa. The molecular weights (MWs) ranged from 88.879 (CaCAMTA4) to 123.588 (SmCAMTA2) kDa, and the theoretical isoelectric point (pIs) ranged from 5.43 (SmCAMTA2) to 7.15 (SmCAMTA1). SmCAMTA1 was predicted to be a basic protein with a theoretical isoelectric point greater than 7 (Table 1). All members were predicted to be located in the nucleus, and none were predicted to contain signal peptides or TMHs. Chromosomal localizations showed that CAMTAs from *Solanum melongena*, *Solanum lycopersicum*, *Solanum pennellii*, *Capsicum annuum*, and *Lycium barbarum* were unevenly distributed across eight chromosomes, respectively (Table 1). Some chromosomes contained two CAMTA genes, such as Chr1 and Chr5 in *S. melongena*; Chr1 and Chr12 in *S*. *lycopersicum*; and Chr11 in *C. annuum*. Nevertheless, Chr1 contained three CAMTA genes in *S. pennellii*.

**Figure 1:**
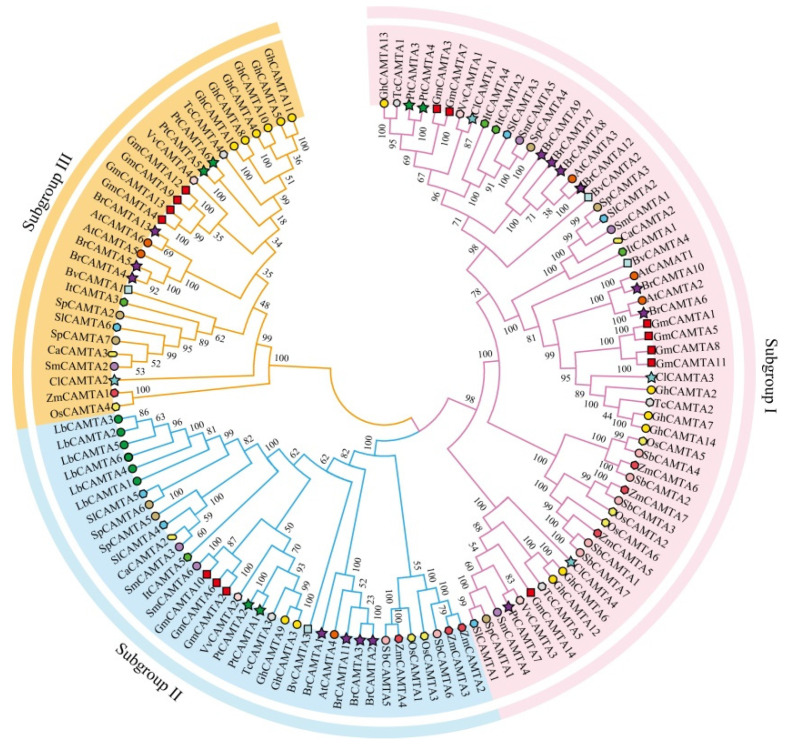
Phylogenetic analysis of SmCAMTAs and known CAMTAs of 18 plant species. Species abbreviations are as follows: *Capsicum annuum* (Ca), *Arabidopsis thaliana* (At), *Solanum lycopersicum* (Sl), *Oryza sativa* (Os), *Vitis vinifera* (Vv), *Solanum melongena* (Sm), *Sorghum bicolor* (Sb), *Citrullus lanatus* (Cl), *Theobroma cacao* (Tc), *Zea mays* (Zm), *Ipomoea trifida* (It), *Populus_trichocarpa* (Pt), *Gossypium hirsutum* (Gh), *Solanum pennellii* (Sp), *Lycium barbarum* (Lb), *Brassica rapa* (Br), and *Glycine_max* (Gm).

To investigate the phylogenetic relationship among Solanaceae CAMTA proteins and those from other plant species, an unrooted phylogenetic tree was constructed using the neighbor-joining (NJ) method in MEGA 11.0.10 software. The 124 CAMTA genes from 17 species were divided into three subgroups: subgroup I (59 members), subgroup II (36 members), and subgroup III (29 members) (Figure 1). Subgroup I included members from 16 plant species (excluding *L. barbarum*), subgroup II contained at least one member from all 17 plant species, and subgroup III was the smallest, exclusively containing CAMTAs from 15 plant species. Members of the LbCAMTA family were specifically distributed in subgroup II. In addition, we found that all CAMTA proteins had conserved domains of CG-1, TIG, ANK, and IQ.

### 2.2. Gene Structure and Conserved Motif Analysis of CAMTA Family Members

**Figure 2:**
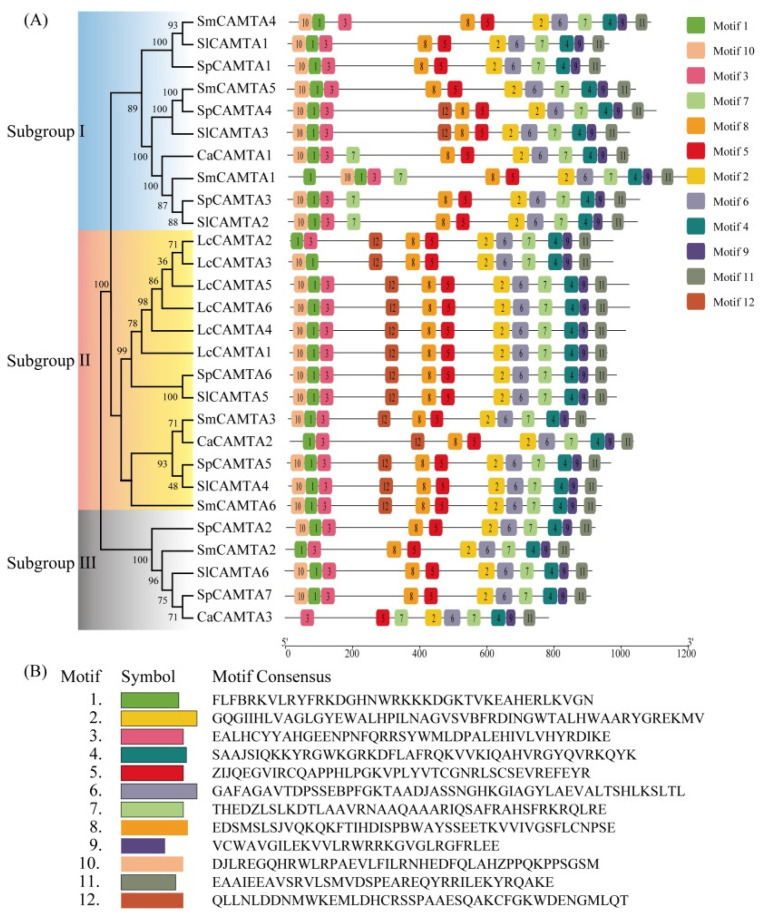
Comparative analysis of conserved motif of CAMTAs family. (**A**) Phylogenetic relationships and conserved protein motifs of CAMTA family. (**B**) Protein sequence of conserved motif.

Figure 2A illustrates a distinct cluster of Solanaceae CAMTA proteins, aligning with the phylogenetic tree constructed using CAMTA sequences from 17 plant species (Figure 1, Table S1). To infer structural variations and potential functional divergence, the coding sequences of CAMTA family genes were analyzed using the MEME tool (http://meme-suite.org/, accessed on 17 April 2020) [35]. The result showed that the CAMTA protein family contains 12 conserved motifs, named motifs 1–12 (Figure 2A,B). The number of motifs varied from 9 to 12. All CAMTA proteins contained motif 2, motif 3, motif 4, motif 5, motif 6, motif 7, motif 9, and motif 11; however, motif 12 was only present in subgroup II of CAMTA. Motif 7, motif 11, and motif 12 were unknown, and the Pfam 36.0 database (http://pfam.xfam.org/, accessed on 8 January 2021) was unable to find the corresponding domain. Motifs 1, 3, and 10 were associated with the CG-1 domain, motifs 2 and 6 with the ANK domain, and motifs 8 and 5 with the TIG domain, while motifs 4 and 9 were related to the IQ domain. Some motifs were specific to members of particular subgroups, suggesting subgroup-specific functions.

**Figure 3:**
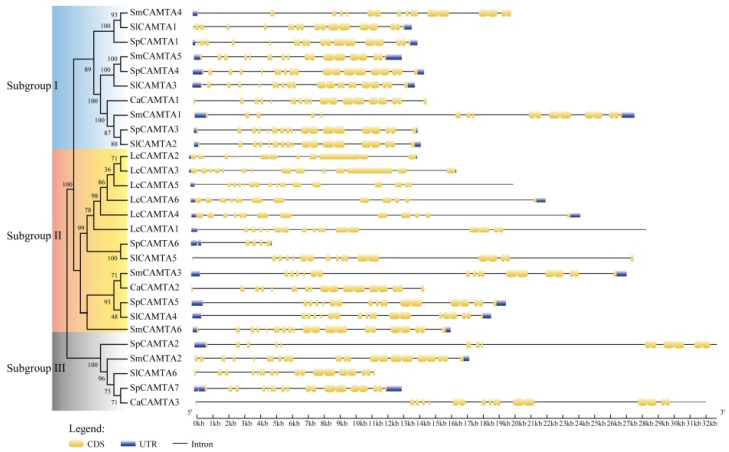
Structural analysis of *CAMTA* gene family members in Solanaceae. Blue color bars are the UTR regions, yellow color bars are the CDS regions, and solid black lines represent introns.

To comprehend the structural characteristics of *CAMTA* genes, exon–intron structures were analyzed. TBtools v2.042 software (https://github.com/CJ-Chen/TBtools-II/tree/2.042/, accessed on 21 September 2023) was employed for this analysis, and the gene structure map of the CAMTA family was generated. The number of introns varied from 5 to 14, with *SmCAMTA2* and *SmCAMTA6* possessing 14 introns each. *SpCAMTA6* and *LcCAMTA2* had fewer introns, namely five and eight, respectively. Notably, introns and exons in the gene structures of subgroup II members within the same branch exhibited significant differences. Although the introns of Solanaceae *CAMTA* genes differed, members with the highest homology, such as *SpCAMTA1*, *SmCAMTA5*, and *SlCAMTA3*, displayed similar gene structures, intron lengths, and the same number of exons. Exon–intron organization was generally consistent within the same group, supporting their close evolutionary relationships (Figure 3). 

### 2.3. Analysis of Cis-Regulatory Element of CAMTA Family Promoter

**Figure 4:**
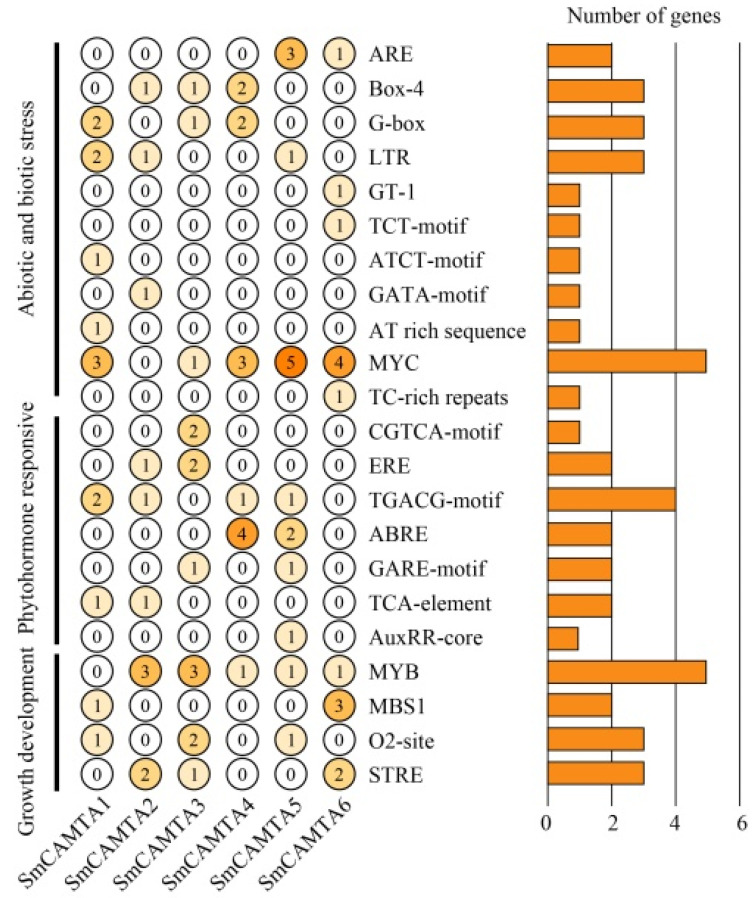
Distribution and function prediction of cis-acting elements in the upstream 2 kb regions of *SmCAMTA* genes. The different colors and numbers of the grids indicate the number of different cis-acting regulatory elements in these *SmCAMTA* genes.

To gain insight into the potential functions of *CAMTA* genes in eggplant, cis-regulatory elements were predicted using the PlantCARE online tool (http://bioinformatics.psb.ugent.be/webtools/plantcare/html/, accessed on 22 July 2022) within the 2000 bp promoter region of each gene [36]. Various cis-regulatory elements were identified in the promoter regions of SmCAMTAs, including elements related to biological and abiotic stress responses (11), plant hormone signaling (8), and growth and development (4), indicating the involvement of *CAMTA* genes in various stress responses. The highest number of biological and abiotic stress responses mainly include GATA-box (*SmCAMTA2*), G-box (*SmCAMTA1*, *SmCAMTA* 3, and *SmCAMTA4*), TCT motifs (*SmCAMTA6*), Box-4 (*SmCAMTA2*, *SmCAMTA3*, and *SmCAMTA4*), and LTR (*SmCAMTA1*, *SmCAMTA2*, and *SmCAMTA5*) (Figure 4). Additionally, several drought-responsive cis-elements (MYC) were present in almost all SmCAMTAs. There were eight phytohormone response elements in the promoter region: TCA responds to salicylic acid (*SmCAMTA1* and *SmCAMTA2*); ABRE responds to abscisic acid (*SmCAMTA4* and *SmCAMTA5*); AuxRR-core respond to auxin (*SmCAMTA5*); and TGACG-motif and CGTCA-motif respond to methyl jasmonate (*SmCAMTA1*, *SmCAMTA2*, *SmCAMTA3*, *SmCAMTA4*, and *SmCAMTA5*). Moreover, growth and development elements such as O2-site, MBS1, and MYB were identified in five genes (*SmCAMTA1*, *SmCAMTA3*, *SmCAMTA5*, and *SmCAMTA6*). These results underscore the diverse roles of each SmCAMTA in coping with diurnal changes, hormonal responses, and various stresses.

### 2.4. Expression Profiling of SmCAMTAs Genes in Different Tissues and under Cold Stress

**Figure 5:**
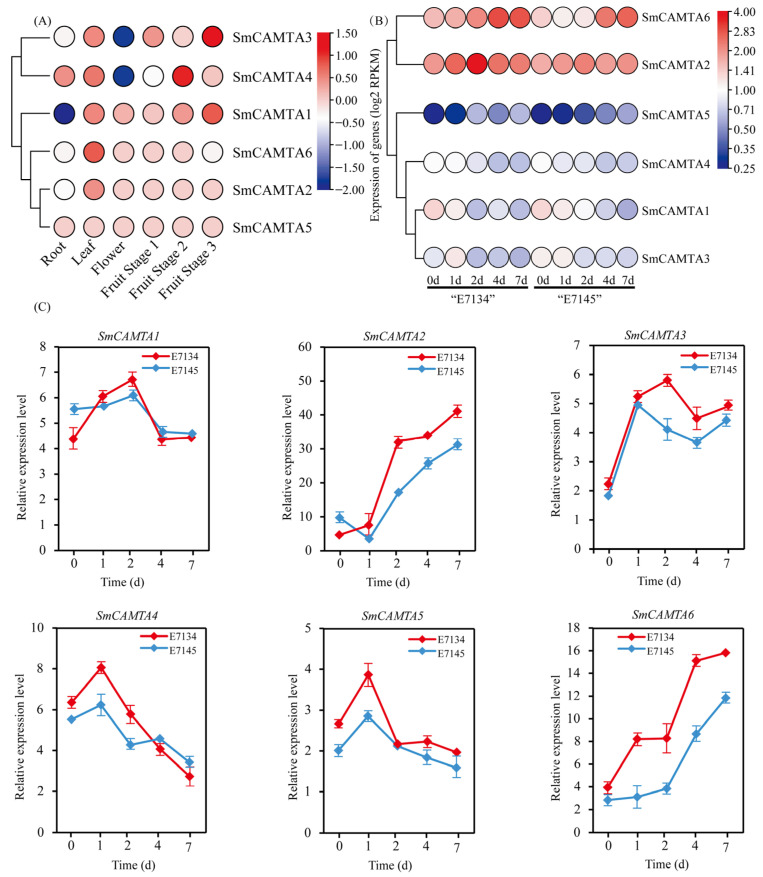
Expression patterns of *SmCAMTAs* under cold stress conditions. Samples at 0 d were set as controls, and the data were calculated by using 2^−ΔΔCt^ method and FPKM. Data are shown as means ± SE (n = 3). (**A**) Expression profiles of *SmCAMTAs* in various developmental stages. (**B**) Relative expression levels of *SmCAMTAs* under cold stress. (**C**) Relative expression levels of *SmCAMTAs* under cold treatment at five time points by qRT-PCR.

To elucidate the functional roles of *SmCAMTA* genes in eggplant during various developmental stages, the expression patterns of *SmCAMTAs* were analyzed across different tissues, including root, leaf, and flower, based on RNA-seq data from a previous study [37] (Figure 5A). *SmCAMTA* genes displayed diverse tissue-specific expression patterns. *SmCAMTA3*/*4* were lowly expressed in root tissue and highly expressed in fruit tissue. The expression levels of *SmCAMTA1* decreased in the root and increased in the leaf and fruit. *SmCAMTA2*/*6* genes were only expressed in leaf tissue. Notably, *SmCAMTA5* did not exhibit detectable expression in the tissue-specific transcriptome.

In the early stages of cold stress, the transcriptomes of both cold-sensitive (‘E7134’) and cold-tolerant (‘E7145’) eggplant varieties were analyzed. The spatiotemporal expression patterns of *SmCAMTA* genes under abiotic cold stress conditions were investigated based on transcriptomic data. As shown in Figure 5B, *SmCAMTAs* were differentially expressed under cold stress conditions. Specifically, all *SmCAMTAs* were induced to some extent by cold at different time points, with *SmCAMTA1*/*3*/*4*/*5* being slightly affected by cold, while *SmCAMTA2* and *SmCAMTA6* transcripts significantly increased within 7 d. These genes actively responded to cold stress, and their transcriptional abundance was notably higher in the cold-tolerant type compared to the cold-sensitive type.

We further explored the expression patterns between the cold-resistant variety ‘E7134’ and the cold-sensitive variety ‘E7145’ under cold stress conditions using qRT-PCR. As shown in Figure 5C, the expression patterns of *SmCAMTAs* varied at different times and in different species. Except for *SmCAMTA2* of ‘E7145’, other genes were upregulated under cold treatment an 0− 1 d, and then the expression levels of *SmCAMTAs* were divided into three categories from 1 d to 7 d under cold treatment time: the transcription levels of *SmCAMTA1*/*3* first increased and then significantly decreased at 2 d; the transcription levels of *SmCAMTA4*/*5* significantly decreased persistently after 2 d; and the transcription levels of *SmCAMTA2*/*6* significantly increased persistently throughout the cold stress treatment. In addition, in the early stage of cold stress, the expression level of *SmCAMTA1/2* in ‘E7145’ was higher than that in ‘E7134’, and at 4–7 days, the expression levels of *SmCAMTA1/4* in ‘E7145’ were higher than that in ‘E7134’. During the entire cold stress period, the expression level of *SmCAMTA3/5/6* in ‘E7134’ was higher than that in ‘E7145’. Importantly, the expression levels of *SmCAMTA2* and *SmCAMTA6* were more than 4.1-fold and 3.9-fold higher after 7 days under cold stress, respectively. This result shows that the similar expression patterns of *SmCAMTA2/6* may have a positive contribution to the cold resistance of eggplants.

### 2.5. Protein–Protein Interaction Analysis of SmCAMTAs

**Figure 6:**
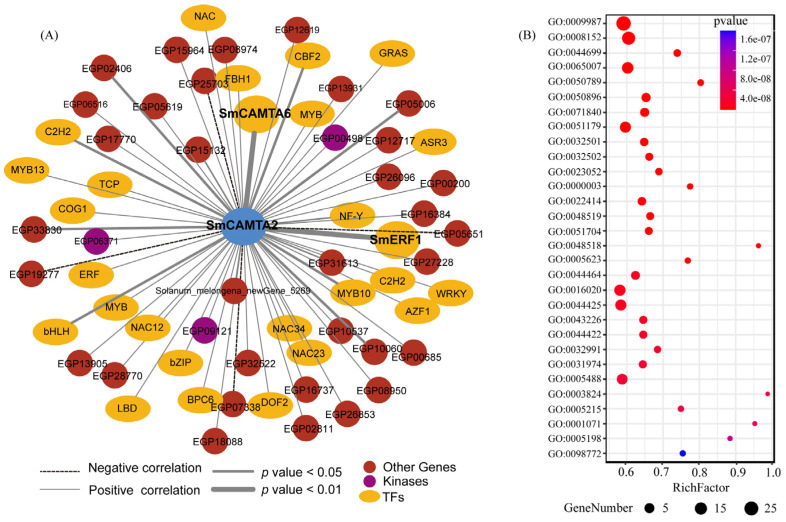
The protein–protein interaction network of CAMTA genes. Network nodes represent proteins; filled nodes represent proteins with known or predicted 3D structures. Edges represent protein–protein associations. Different colors represent various types of interactions.

Biological macromolecules typically engage in physiological functions through interactions with other proteins, forming complexes. Therefore, investigating these interaction relationships is crucial for understanding the function of CAMTA. The whole-transcriptome protein interaction network of eggplant was constructed using homologous mapping and domain interaction methods with *Arabidopsis* as the reference species. The subnetwork of the SmCAMTA2 core protein was extracted (Figure 6A and Table S4). A total of 62 non-redundant potential interacting proteins of *SmCAMTA2* were identified, comprising nine types of transcription factors (TFs) (C2H2, GRAS, bHLH, MYB, WRKY, TCP, AP2/ERF, NAC, DOF), three kinases (phosphoribulo, lectin, and shikimate kinases), as well as several enzymes and RNA-binding proteins. In addition, 93.5% of interacting protein genes were positively correlated with SmCAMTA2 (*p* < 0.05) under cold stress, and GO enrichment analysis showed that they were related to “DNA binding”, “hormone metabolism processes”, “photosynthesis”, and “catalytic activity” functions (Figure 6B). Therefore, it is suggested that SmCAMTA2 may recruit plant hormone-related proteins under cold stress. Notably, a strong interaction was observed between *SmERF1*, *SmCAMT6*, and *SmCAMTA2* (*p* < 0.001) (Figure 6A). 

**Figure 7:**
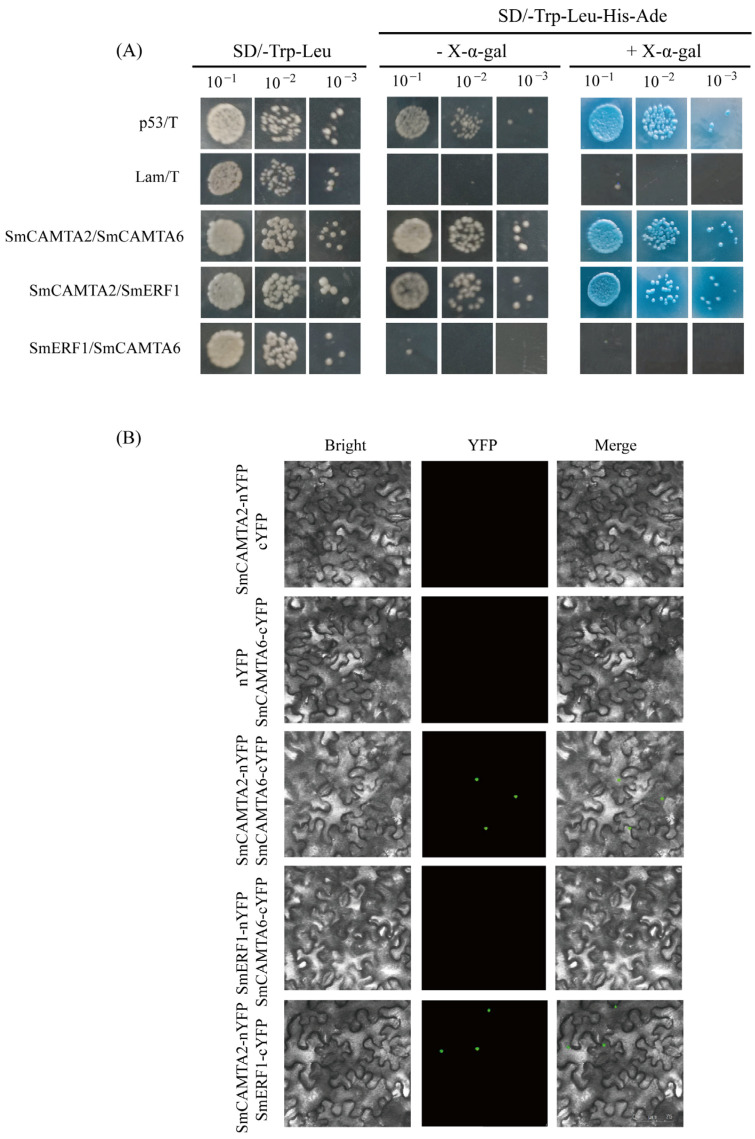
Interaction relationship detected between SmCAMTA2 and SmCAMTA6 using GAL4-based Y2H system and bimolecular fluorescence complementation. (**A**) Yeast two-hybrid experiment on SmCAMTAs. The interaction relationship was examined on SD/- Trp/- Leu/- His/− Ade medium containing X-a-gal. The pGBKT7-53+pGADT7-T vector and pGBKT7-Lam+pGADT7 were used as positive and negative controls, respectively. (**B**) In BiFC assays, *SmCAMTA2*-nYFP, cYFP, and nYFP and *SmCAMTA6*-cYFP, *SmCAMTA2*-nYFP, and *SmCAMTA6*-cYFP were co-transformed in *N. benthamiana* leaf cells, respectively; bar = 75 µm. Bright field, GFP field, and merged field indicate the state of fluorescent protein under three different channels.

A GAL4-based yeast two-hybrid (Y2H) system and bimolecular fluorescence complementation (BiFC) were employed to investigate the relationships between *SmCAMTA2*, *SmCAMTA6*, and SmERF1. As shown in Figure 7A, the positive control and experimental group transformants grew on SD/- Trp- His-Ura medium containing X-a-gal and turned blue, indicating that *SmCAMTA2* interacts with *SmCAMTA6* and *SmERF1*. Subsequently, a BiFC experiment was conducted on tobacco leaves (Figure 7B), confirming the interaction between *SmCAMTA2* and SmERF1, as well as *SmCAMTA6*. However, there was no interaction observed between SmCAMTA6 and SmERF1.

### 2.6. SmCAMTA2 and SmCAMTA6 Regulated the Transcription of Cold-Related CBF Genes under Cold Stress

**Figure 8:**
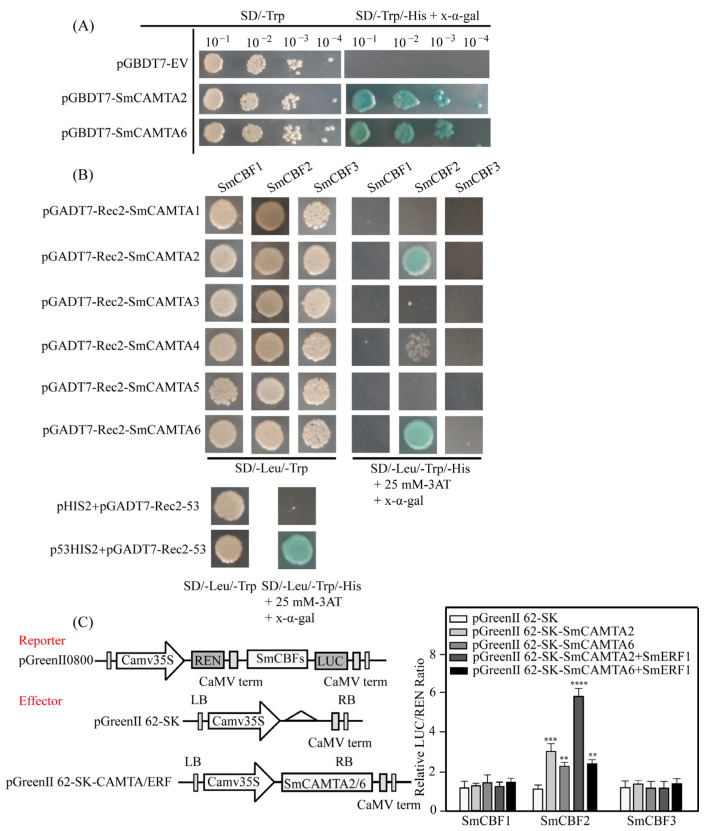
Identification of the interaction between *SmCAMTA2*/*6* and SmCBFs promoters. (**A**) Transcriptional activation associated with *SmCAMTA2*/*6* in yeast cells. (**B**) Interaction between *SmCAMTA2*/*6* and SmCBFs promoters system. pHIS2+pGADT7-Rec2-53 and p53HIS2+pGADT7-Rec2-53 were the negative control and positive control, respectively. (**C**) Calculation of structure and LUC/REN ratio used in transient trans activation analysis as the final transcriptional activity. Blank carriers are used as effectors in control experiments. Using the promoter of CBF in LRA, each value represents the average of three biological replicates. Values represented as mean ± standard deviation, and *p* values of <0.01 (**), <0.001 (***), and <0.001 (****) were considered to be significant statistically.

To investigate the functions of SmCAMTA2 and SmCAMTA6 and their potential transcriptional activation activity, trans-activation assays were conducted on transcription factors. In the yeast system, both SmCAMTA2-BD and SmCAMTA6-BD yeast cells, as well as positive controls, exhibited robust growth on selective media (SD/- Trp/- His + X-α-gal) and turned blue. Conversely, negative controls (BD) could not grow on selective media (Figure 8A). This observation provides additional evidence supporting the role of SmCAMTA2 and SmCAMTA6 in activating transcription factors.

The AtCBFs cold response pathway plays a central role in cold acclimation [38]. Previously, it was reported that the expressions of DREB1/CBF genes were regulated by many transcription factors, indicating that the expression regulation of these genes was highly complex. To explore the transcriptional regulation of SmCBFs by *SmCAMTA2* and *SmCAMTA6*, and the involvement of *SmERF1* in the regulation of SmCBFs genes, yeast one-hybrid experiments and the LUC/REN reporting system were employed. The results indicated that the co-transformed single colonies of the SmCBF control group grew normally on SD/- Leu/- Trp medium without 3-AT inhibitors. In contrast, the yeast colonies of the negative control group did not grow on a plate containing 25 mM 3-AT, while the colonies of the positive experimental group exhibited normal growth in size and morphology (Figure 8B). In addition, the co-transformation of SmCBFs pro LUC and pGreenII 62-SK into tobacco leaves showed very low luciferase luminescence, while luminescence was significantly enhanced through the co-transformation of *SmCBF2* pro LUC with *SmCAMTA2*-62-SK and *SmCAMTA2*-62-SK. LAR confirmed that *SmCAMTA2* and *SmCAMTA6* activated the *SmCBF2* promoter in tobacco leaves (Figure 8C). Furthermore, the LUC/REN ratio showed a significant, approximately twofold increase when SmERF1-62-SK and SmCAMTA2-62-SK were co-presented, with no significant change observed when they were co-present with SmCAMTA6-62-SK. These results suggest that SmERF1 promotes the expression of CBF2 by interacting with SmCAMTA2 and binding to the promoter element of SmCBF2.

## 3. Discussion

CAMTA transcription factors play a pivotal role in calcium/calmodulin signaling pathways, mediating gene transcriptional regulation, which is an essential process for plants to respond to exogenous hormones and abiotic stress [39,40,41]. Eggplant development exhibits distinct specificity, with growth stimulated by various biotic and abiotic stressors and hormones. This suggests a potential crucial biological function for CAMTA in eggplant. With the completion of genome sequencing for eggplant, we were able to conduct a comprehensive analysis of CAMTA. In this study, we identified the CAMTA gene family of Solanaceae at the whole-genome level. The number of CAMTAs ranged from three to seven. The copy number of the homologous CAMTA gene family generally varies among different species, which is caused by different gene gain and loss rates. The copy number variation of gene families provides a genetic basis for the innovation and diversification of species phenotypes and is closely related to the evolution of genome size and species differentiation in organisms. In this study, we comprehensively analyzed the gene structure and phylogeny of the CAMTA gene family. The promoter elements and transcriptional expression patterns of eggplant CAMTAs were also analyzed. Twenty-eight CAMTA genes were identified in Solanaceae. HMMER 3.0 (hidden Markov model, HMM) online software (http://eddylab.org/software/hmmer3/3.0/, accessed on 15 May 2018) software and the blastp program of the hidden Markov model were employed to identify CAMTA members of other species at the whole-genome level. The intersection of the results of the two outputs was taken for further conservative domain analysis. Finally, CAMTA members confirmed to contain the CG-1 domain were identified.

The phylogenetic tree results show that the closer the clustering relationship, the more likely it is to have similar functions [42]. Phylogenetic analysis revealed that CAMTA genes can be divided into three subgroups, and the distribution of *Arabidopsis* CAMTAs in other phylogenetic groups aligns with that of this specific phylogenetic group. The motif composition of CAMTA proteins in Solanaceae is highly conservative, indicating a relatively consistent function for CAMTAs. Notably, some genes lack certain motifs, potentially contributing to the functional diversity observed in CAMTA genes. Gene structure analysis identified a high similarity in the CAMTA homeotic genes of Solanaceae, suggesting a highly conservative gene structure [43,44,45]. The relative position and number of introns within the same branch were also conservative, but variations across different branches contributed to the diversification of gene function. Genetic phylogenesis, gene classification, and gene structure analysis can provide valuable insights for more accurate and convenient exploration of similar gene families and their functions. 

The function of CAMTA genes in *Arabidopsis* has been extensively studied, providing insights into the potential functions of related genes in Solanaceae. Evolutionarily related genes often share similar functions. For instance, *AtCAMTA3* in Arabidopsis positively regulates the CBF2 gene, playing a crucial role in plant low-temperature stress response, and influencing the biosynthesis of salicylic acid (SA) [23]. *SmCAMTA2* and *SmCAMTA6* were significantly and continuously upregulated by low-temperature stress; it was speculated that *SmCAMTA2* and *SmCAMTA6* may play a role in low-temperature stress through biochemical reactions similar to *AtCAMTA3*. At the same time, *AtCAMTA3* was also a drought stress response factor [46], suggesting that *SmCAMTA2* and *SmCAMTA6* may also have functional diversity. Pandey et al. found through gene chip research that 17 genes related to auxin were upregulated in *AtCAMTA1* mutants [39]. Both *AtCAMTA1* mutants and RNAi-mediated CAMTA1 inhibitory transgenic lines displayed a phenotype of auxin hypersensitivity to hypocotyl elongation, indicating the involvement of *AtCAMTA1* in auxin signaling pathways and its role in regulating plant growth and development. Yang et al. observed differential expression of seven CAMTA genes during tomato fruit development and maturation, suggesting their potential role in the regulation of tomato fruit development [47]. It was speculated that CAMTA homeotic genes in eggplant were involved in the growth and development of tissues and organs. Research in Arabidopsis showed that *AtCAMTA2* is an activated transcription factor of *AtALMT1* (aluminum activated malic acid transporter 1) [48], and *AtCAMTA3* plays a negative regulation role in SA-mediated plant defense response [30,49]. The loss of repression of *AtCAMTA1*, *AtCAMTA2*, and *AtCAMTA3* can induce the initiation of plant defense genes and systemic resistance [23]. *MeCAMTA3* in cassava regulated the resistance of cassava to bacterial wilt disease by regulating various immune responses during the interaction between cassava and *Xanthomonas flavescens* [50]. Therefore, it is speculated that *SmCAMTA4* and *SmCAMTA5* genes exhibit functional diversity and may be involved in hormone signal transduction, growth and development regulation, low-temperature stress response, salt stress, and defense response. Among them, *SmCAMTA2* and *SmCAMTA6* genes are identified as important candidate genes for further studying the molecular mechanism of eggplant response to low-temperature stress. This study represents the first systematic analysis of the *CAMTA* gene, laying the foundation for further research on the function of the *CAMTA* gene in Solanaceae.

The results of protein interaction network analysis revealed a complex regulatory network involving directly functional proteins which regulated plant growth and development through transcriptional regulation of *SmCAMTAs* and other functional genes. *SmCAMTA2* and *SmCMATA6* exhibited high expression levels under cold conditions (Figure 5B,C), suggesting a potential role in binding to the CM cis-element in the *SmCBF2* promoter. This binding could regulate the expression of downstream *CBF* genes, similar to their orthologs such as *AtCAMTA3* and *AtCAMTA2*. Similarly, the interaction analyses of SmCAMTAs demonstrated that *SmCAMTA2* and *SmCAMTA6* were directly related to CBF/DREB1C. It is important that the interaction of SmCAMTA2 and SmERF1 promoted the transcriptional activity of *SmCBF2*, which may be an important mechanism in eggplant’s response to cold stress. Previous studies have shown that AtCAMTA1 and AtCAMTA2 work synergistically with CAMTA3 at low temperatures (4 °C), inducing high transcriptional expression of *CBF1*, *CBF2*, and *CBF3*, thereby enhancing plant frost resistance. Additionally, *AtCAMTA1*, *AtCAMTA2*, and *AtCAMTA3* collectively inhibit SA biosynthesis at warm temperatures (22 °C) [23]. Functional analyses using AtCAMTA mutants indicated that *AtCAMTA3* negatively regulated immunity triggered by flg22 [30]. *SmCAMTA1* and *SmCAMTA5* were an ortholog of *AtCAMTA5*, which speculates that its function may be related to the pollen pollination and development of eggplant [51]. It has been reported that the CAMTA family is involved in fruit development and abiotic stress processes in tomato [46], soybean [26], and other plants [52]. In tomato, for example, *SlCAMTA3* expression is scarce in leaves in the seedling and flowering stages, while it is highly expressed in roots in the seedling stage [53]. In the case of eggplant, the expression of the *SmCAMTA3* and *SmCAMTA4* genes is highly observed in the leaves but is lowly detected in eggplant flowers (Figure 5A). The results of protein–protein interaction network analysis indicate that these three proteins are directly related to each other, suggesting their potential collaborative role in responding to cold stress during the reproductive growth stage of eggplant.

## 4. Methods and Materials

### 4.1. Plant Materials, Growth Conditions, and Stress Treatments

Two varieties of *S. melongena*, ‘E7134’ (a cold-tolerant variety) and ‘E7145’ (a cold-sensitive variety), were utilized for cold treatment in this study. Both eggplant materials were preserved and provided by the Sichuan Academy of Agricultural Sciences. Seedlings were cultivated in a greenhouse under controlled conditions at 26 ± 1 °C, 65% relative humidity, and a 16/8 h light/dark period in the agricultural science area of Sichuan Province (Chengdu, China). For the cold treatment, seedlings with 5 to 6 leaves were transplanted into plastic pots. Subsequently, all seedlings were exposed to cold stress for 7 days, maintaining a temperature of 5 °C during the day and 10 °C during the night. Sampling was conducted at 0, 1, 2, 4, and 7 days after the initiation of cold stress, with three biological replicates collected at each time point. The samples from both *S. melongena* varieties’ seedlings were promptly frozen in liquid nitrogen and stored at −80 °C for subsequent analyses.

### 4.2. Identification of CAMTA Genes 

All *CAMTA* genes in the complete Solanaceae genome were identified from Solanaceae species (https://solgenomics.net/, accessed on 14 October 2023) using HMMER 3.0 online software (http://eddylab.org/software/hmmer3/3.0/, accessed on 15 May 2018) [54]. HMM profiles for the *CAMTA* gene family (Pfam03859: CG-1; Pfam12796: ankyrin repeats; Pfam01833: TIG domain; and Pfam00612: IQ) were obtained from Pfam 36.0 (http://pfam.xfam.org/, accessed on 8 January 2021) [55]. The whole-genome sequences of the other 16 plants (*C. annuum*, *A. thaliana*, *S. lycopersicum*, *O. sativa*, *V. vinifera*, *S. bicolor*, *C. lanatus*, *T. cacao*, *Z. mays*, *I. trifida*, *P. trichocarpa*, *G. hirsutum*, *S. pennellii*, *L. barbarum*, *B. rapa*, and *G. max*) were downloaded from the Plant JGI Database phytozome v13 (https://phytozome.jgi.doe.gov/pz/portal.html, accessed on 6 October 2022) [56]. These profiles served as queries against whole-genome peptide sequences of *A. thaliana* retrieved from whole-genome sequence data. After eliminating redundant sequences, the identity of CAMTA proteins containing functional protein domains was confirmed using NCBI-CDD (National Center for Biotechnology Information—Conserved Domain Database, https://www.ncbi.nlm.nih.gov/Structure/bwrpsb/bwrpsb.cgi, accessed on 10 September 2021) [57]. Any sequences with missing structural domains were excluded. To understand the genomic organization of *CAMTA* genes, information on their chromosomal positions was extracted from gff3 files and visualized using TBtools v2.042 (https://github.com/CJ-Chen/TBtools-II/tree/2.042/, accessed on 21 September 2023) [58]. A detailed list of accession names for CAMTA genes is provided in Table 1.

### 4.3. Phylogenetic Analysis and Classification

The amino acid sequences of the 124 identified CAMTA proteins (refer to Table S3) were utilized for phylogenetic analysis. Multiple sequence alignments of all selected CAMTA sequences were conducted using the MUCLE method with default parameters [59]. Subsequently, unrooted phylogenetic trees of the CAMTA proteins were constructed using MEGA 11.0.10 software (https://www.megasoftware.net/, accessed on 23 April 2021) [60]. The parameters included Poisson correction, pairwise deletion, and a bootstrap test with 1000 replicates. Furthermore, the phylogenetic tree was visually enhanced using EvolView v3 (https://www.evolgenius.info//evolview/#login, accessed on 26 May 2020) [61].

### 4.4. Motif Composition and Gene Structure Analysis

To identify additional conserved motifs beyond the CAMTA proteins, the Multiple Em for Motif Elicitation tool (http://meme-suite.org/tools/meme, accessed on 17 December 2022) [35] was employed. Specific parameters, including maximum width, minimum width, and maximum number of motifs, were set at 5, 150, and 12, respectively. The motifs were sequentially numbered based on their order in MEME. Signatures common to genes within one of the three similarity groups were assigned as group-specific motifs. The outcomes were visualized using TBtools v2.042 (https://github.com/CJ-Chen/TBtools-II/tree/2.042/, accessed on 21 September 2023) [58] to protein structures. The CDS and DNA sequences of CAMTAs were obtained from Solanaceae species (https://solgenomics.net/, accessed on 14 October 2023) and GSDS v2.0 (Gene Structure Display Server, http://gsds.cbi.pku.edu.cn/, accessed on 27 May 2020) [59] was utilized to analyze gene structures, providing a comprehensive visualization of the exon–intron organization of *CAMTA* genes.

### 4.5. Analysis of Cis-Acting Elements in the Promoter Region 

The PlantCARE database (http://bioinformatics.psb.ugent.be/webtools/plantcare/html/, accessed on 22 July 2022) was utilized to identify cis-acting elements within the 2000 bp sequence upstream of the transcription start site of *SmCAMTA* genes [36]. The distribution map of cis-acting elements was visualized via TBtools v2.042 (https://github.com/CJ-Chen/TBtools-II/tree/2.042/, accessed on 21 September 2023) [58].

### 4.6. Gene Expression Analysis 

Expression data for SmCAMTA family members from different tissues were obtained from the China National Center for Bioinformation/Beijing Institute of Genomics, Chinese Academy of Sciences (GSA: CRA013268) and are publicly accessible at https://ngdc.cncb.ac.cn/gsa. Log2 (1 + FPKM (treatment)/1 + FPKM (control)) were calculated using FPKM to indicate the fold change in gene expression levels. Subsequently, the expression levels of selected plant *SmCAMTA* genes were analyzed using TBtools v2.042 (https://github.com/CJ-Chen/TBtools-II/tree/2.042/, accessed on 21 September 2023) [58].

### 4.7. RNA Isolation and qRT-PCR Analysis

Total RNA was extracted from leaves subjected to different abiotic stresses using the RNA Prep Pure Plant Kit (Tiangen Biochemical Technology, Beijing, China: DP201101X). The concentration and purity of the RNA were assessed using a Nanodrop micro spectrophotometer (Thermo Scientific, Waltham, MA, USA) and agarose gel electrophoresis. The first strand of cDNA, derived from mRNA, was synthesized using HiScript^®^Q RT SuperMix (Vazyme, Nanjing, China). Quantitative real-time PCR (qRT-PCR) was performed using SYBR-green fluorescence with a QuantStudioTM Real-Time PCR System (Thermo Fisher Scientific). Six CAMTA genes were selected to validate expression patterns under cold stress. The data were normalized by the internal control gene β-actin and the 2^−∆∆CT^ analysis method was utilized to calculate relative expression levels [62]. The data were finally visualized as mean values ± standard error (±SE). Primers are listed in the Table S2.

### 4.8. Protein–Protein Analysis of SmCAMTAs 

For the yeast two-hybrid experiment, the coding regions of *SmCAMTA2* and *SmERF1* were amplified and fused in-frame downstream of the GAL4 DNA-binding domain in the pGBKT7 vector, while SmCAMTA6 was fused in the pGADT7 vector. Primers are listed in Table S2. The constructs were transformed into strain AH109 (*Saccharomyces cerevisiae*) and grown on SD/- Trp and SD/- Trp/- His/- Ura medium, respectively. Autoactivation activity was examined on SD/- Trp/- His/- Ura medium containing X-a-gal. The pGBKT7-53+pGADT7-T vector and pGBKT7-Lam+pGADT7 were used as positive and negative controls, respectively.

For the bimolecular fluorescent complementation of the *SmCAMTA2/6* transcription factors in intact cells, the plant transient expression vectors pXY103 (cYFP) and pXY104 (nYFP) were utilized. A transient transformation system was established using 1M MgCl_2_ and 0.5 M acetosyringone. The construct recombinant plasmids *SmCAMTA2*-cYFP and *SmCAMTA6*-nYFP were then transferred into tobacco leaves using *Agrobacterium* strain (GV3101), with *SmCAMTA2*-cYFP+nYFP and cYFP+*SmCAMTA6*-nYFP as the control. The fluorescence of fusion proteins was detected by laser scanning confocal microscopy, Leica (Zeiss, Jena, Germany).

### 4.9. SmCAMTAs Participate in Transcriptional Regulation of SmCBFs

The effector vector pGADT7-SmCAMTAs and the reporter vector pHIS2-SmCBFs pro were constructed and co-transformed into the yeast strain Y187. The transformed yeast cells were cultured at 28 ℃ on SD (SD/-Trp/-Leu) medium until colonies grew normally. After dilution based on a gradient, they were applied to SD (SD/-Trp/-Leu/-His) medium with a final concentration of 20 mM 3-AT inhibitor for inverted cultivation, and colony morphology was observed. The primers used are listed in Table S3.

The promoter sequences of SmCBF genes were cloned into the pGreenII 0800-LUC double-reporter vector. The full coding sequences (CDS) of *SmCAMTA2*, *SmCAMTA6*, and *SmERF1* were then inserted into the pGreenII 62-SK effector vector. Co-infiltration of the reporter and effector plasmids was performed in *Nicotiana benthamiana* leaves [63]. The Dual-Luciferase Assay Kit (Promega, Madison, WI, USA) was utilized to analyze luciferase activity. Results are expressed as the LUC (firefly luciferase)/REN (renilla luciferase) ratio, representing an average of six replicates.

## 5. Conclusions

This study identified six members of the CAMTA gene family in Solanaceae through genome-wide analysis, categorizing them into three subgroups—subgroup I, subgroup II, and subgroup III—based on their phylogenetic relationships. Comprehensive analyses of phylogenetics, gene structure, and motif composition were conducted in Solanaceae. Expression pattern analyses of SmCAMTAs revealed distinct expression profiles in various tissues of eggplant. RNA-seq data and qRT-PCR results indicated that *SmCAMTA2*/*6* genes positively responded to cold stress, and the interaction between *SmCAMTA2* and *SmERF1* facilitated the transcription of *SmCBF2*. These findings offer crucial insights into the significant role of CAMTA in responding to cold stress and lay the groundwork for further exploration of the functional aspects of Solanaceae CAMTA gene family members.

## Figures and Tables

**Table 1:** Basic physical and chemical analysis of SmCAMTAs. ORF, Open Reading Fame; AA, number of amino acid residues; MW, Molecular Weight; pI, theoretical isoelectric point; Chr, chromosome location.

Species	Gene ID	Name	Subgroup	ORF (bp)	AA	pI	MW (kDa)	Chr
*Solanum melongena*	SMEL4.1_01g027530.1.01	SmCAMTA1	Subgroup I	2769	923	7.15	104.200	1
	SMEL4.1_11g009470.1.01	SmCAMTA2	Subgroup III	3309	1103	5.43	123.588	11
	SMEL4.1_03g010310.1.01	SmCAMTA3	Subgroup II	2889	963	5.83	107.849	3
	SMEL4.1_05g010190.1.01	SmCAMTA4	Subgroup I	2865	955	6.48	106.575	5
	SMEL4.1_05g021930.1.01	SmCAMTA5	Subgroup I	2703	901	6.78	102.294	5
	SMEL4.1_01g009270.1.01	SmCAMTA6	Subgroup III	3147	1049	5.65	118.563	1
*Solanum lycopersicum*	Solyc01g057270.2.1	SlCAMTA1	Subgroup I	2871	957	8.92	107.785	1
	Solyc01g105230.2.1	SlCAMTA2	Subgroup I	3120	1040	5.47	117.417	1
	Solyc04g056270.2.1	SlCAMTA3	Subgroup III	3063	1021	5.76	114.146	4
	Solyc05g015650.2.1	SlCAMTA4	Subgroup II	2805	935	5.89	105.304	5
	Solyc12g035520.1.1	SlCAMTA5	Subgroup II	2919	973	6.25	108.982	12
	Solyc12g099340.1.1	SlCAMTA6	Subgroup III	2945	915	6.74	103.939	12
*Solanum pennellii*	Sopen01g021880.1	SpCAMTA1	Subgroup I	2835	945	9.02	106.506	1
	Sopen01g026290.1	SpCAMTA2	Subgroup III	2760	920	8.07	104.079	1
	Sopen01g047740.1	SpCAMTA3	Subgroup I	3147	1049	5.45	118.478	1
	Sopen04g025150.1	SpCAMTA4	Subgroup I	3294	1098	5.51	122.826	4
	Sopen05g011110.1	SpCAMTA5	Subgroup II	2892	964	5.72	108.263	5
	Sopen12g015500.1	SpCAMTA6	Subgroup II	2919	973	6.25	108.922	12
	Sopen12g034010.1	SpCAMTA7	Subgroup III	2730	910	6.66	103.230	2
*Capsicum annuum*	CA08g15740	CaCAMTA1	Subgroup I	3054	1018	5.81	114.798	8
	CA11g07570	CaCAMTA2	Subgroup II	3069	1023	6.19	115.200	11
	CA11g10930	CaCAMTA3	Subgroup III	2355	785	6.01	88.879	11
*Lycium barbarum*	XP_060191074.1	LbCAMTA1	Subgroup II	2838	946	6.24	105.811	11
	XP_060191073.1	LbCAMTA2	Subgroup II	2826	962	6.04	107.508	11
	XP_060191072.1	LbCAMTA3	Subgroup II	2898	966	6.13	107.783	11
	XP_060191071.1	LbCAMTA4	Subgroup II	3003	1001	6.09	111.977	11
	XP_060191070.1	LbCAMTA5	Subgroup II	3024	1008	6.04	112.836	11
	XP_060191069.1	LbCAMTA6	Subgroup II	3033	1011	6.09	113.150	11

## Data Availability

The transcriptome raw data are available from the online web site China National Center for Bioinformation/Beijing Institute of Genomics, Chinese Academy of Sciences (https://ngdc.cncb.ac.cn/gsa accessed on 6 Jan 2023, GSA: CRA0132687).

## Supplementary Materials

The following supporting information can be downloaded at: https://www.mdpi.com/article/10.3390/ijms25042064/s1**.**
