# Supplementary material for: Identification and Molecular Characterization of the CAMTA Gene Family in Solanaceae with a Focus on the Expression Analysis of Eggplant Genes under Cold Stress
Source: Int J Mol Sci. 2024 Feb 8;25(4):2064. doi: 10.3390/ijms25042064 (PMC10888690; doi:10.3390/ijms25042064)
Supplement: Supplementary file 1 [file ijms-25-02064-s001.zip › ijms-2813236-supplementary.pdf]

Table S1. All sequences used to construct phylogenetic tree

>SmCAMTA1

MVNSIKFILHDVLKDLLLVVINVLEVRVQGKAFYLIGETLRRWVLCDKKVQLYKVVVRST

MLYGVKCWSVKNSHIRKIKVAEMRMLRWMHGHIKKDKIRNEVIRHKVGVSLMKDKIWEVR

LRCFGHIKRRCTDASVRRCERLTMDDFKRGRDITQILSEVQHRWLRPAEICEILRNHRKF

HLTPEAPYRPVSGSVFLFDRKVLRYFRKDGHNWRKKKDGKTVKEAHEKLKVGSIDVLHCY

YAHGEEDDNFQRRSYWMLEQDLMHIVFVHYLEVKGNKVNVSCIRSTKPAHQNYLNDCSLS

DSFPTRHKNLASANTDSASLASTLTEAHEEAESEDSHQACSRFHSYPDRASGMDSHVVEN

RDTICSSYGSPPSSVEYTPLPSIDGASKCDLGNFASAPQRTIDLGSQEPVSQHYSNGEMV

CQDDFKNNLSVHGNWQYSFGHSPLQFHGQNVNQDMIADSSYDLGLPSDLLTVRRQSYLYP

DDQEEKLTQLNLQYLNSLVEVQGDINQENSMDMLGLGDYSMSKQPHLSSFKLEEGLKKVD

SFSRWVAKELEDVEELHMHPSNRLSWNVIDTEDDGSCLPSQLHVDSDSLDPSLSQEQVFS

IIDFSPNWAYSNLETKVLITGRFLKSESELLKYKWSCMFGEVEVPAEVLADGVLRCHAPP

HKPGVLPFYMTCSNRLACSEVREFEYRFGPYQEFGAADVSKTEMHLLERIENLLSLGPVS

SCRNSDSLEASKERQSTVNKIICMMEEENQQMIQSSSYYDTSQCRVEEDLFSERKLKQNF

YAWLIHQVTDDGKGRTHLDDEGQGILHLVAALGYDWALKPILASGVSVDFRDMNGWTALH

WAAFYGREKTVVGLVSLGASPGALTDPSSEFPLARTPADLASANGHKGISGFLAESSLTT

HLSKLTVTDAKEELALEVCGAKVGERVAECVAVATTGNDVPDVLSLKDSLAAIRNATQAA

ARIHQIFRVQSFQRKQIIEHCDNELSSDENALSIVASRASKLGQNNVIAHAAAIKIQKKF

RGWNKRKEFLLIRQKIVKIQAHIRGHQVRKKYKPIIWSVGILEKVILRWRRKRSGLRGFR

SEAVMNKSSTIDDSLPEDDYDFLKEGRKQTEVRMQKALSRVKSMTQYPEGRAQYRRLLTA

AEGLREVKDGPTQIPEIPEDAIYPEEDLFDVESLLDDDTFMSIAFE

>SmCAMTA2

MESSVSGRLVGWEIHGFRTMQGGTIVLFDRKMLRNFRRDGHNWKKKKDGKTVKEAHEHLK

VGNEERIHVYYAHGEDNTTFVRRCYWLLDKTLEHIVLVHYRETQEGSSNSTVAQGSPAAP

VSSGSALSDPADLSSSWVLSPDSAVDQQYPASQHANLETNRDMTVQNHEQRLLEINTLEW

DDLLAPGDSNKIIATQQGGKTAYVQHTLYEQHNLRELNGYSLNGVSSSPERMSTVNNLNE

ITFQTMDGQMTEYGVMTVSTGDSFDSLNNNRLQTQDSFGRWMNYLITDSSGSTDDPTPES

SVSTGQSNAREWVFNITEISPAWAPSNEETKIFVIGHFHGAQSHLESSSLHCVCGDACFP

AEVLQPGVYRCIVSPQTPGVVNIYLSFDGTKPISQVMSFEFRAPLVHVWTEPPESKSNWD

EFRNEMRLAHLLFSTSKSLNILSSKINQDLLKDAKTFAGKCSHIIDDWACLIKSIEDKKL

SVPHAKDCLFELSLKTRLQEWLLERIVEGCKISEHDEQGQGVIHLCAILGYTWAVYPFSW

SGLSLDYRDKYGWTALHWAAYYGREKMVATLLSAGAKPNLVTDPTSENLGGCTASDLASK

NGHEGLGAYLAEKALVAQFKDMTLAGNISGSLQTTTESINPGNFTQEELNLKDSLAAYRT

AADAAARIQAAFRERELKVRTEAVESSNTEMDARSIIAAMKIQHAFRNYEMQKQLAAAAR

IQYRFRTWKMRKEFLHMRRQAIKIQSVFRGFQVRRQYGKIIWSVGVLEKALFRWRLKRKG

FRGLKLQSSQVVHKPDDVEEDFFQASRKQAEERIERSVVRVQAMFRSKQAQEQYRRMKLE

HNKATLEYEGTRDPDTEMG

>SmCAMTA3

MKSSDEFLVPTRALGNDGYDINDLIREAQTRWLKPAEVLFILRNHENHQLSSGSLFLFNK

RILRFFRKDGHSWRKKKDGRTVGEAHERLKVGNAEALNCYYAHGEQNPNFQRRSYWMLDP

AYDHIVLVHYRDITEGRQNPAFMSESSPISSAFSPSPSSYSTQHTGSTVIASESYEQYQN

QPSPAEICSDAIINNYGTLDTTGRKEEVISSPGLEMSQALRRLEEQLSLNDDSFKEIDPL

YADAINDDSSLIQMQGNSNSLLLQHHSDICHDPSLGPRRDTANGEPEGIPNKPLSISLTH

PWSQKQNKYQQVI

>SmCAMTA4

MDHSTQPLGLLLLQVFSSSTFLCQYLEQILQEAHRRWLRLHEVCEILRNHQKFYLTQEPP

LKPPGSSMFLFDRKVVPYFRKDGHHWRKKKDGKTVKEAHEKLKVINFFTDCRQEALMLFT

VTMPMGRTTSTFRGDVIGCLEKFYGRRNGECVGKKMTADGKEIENTVLGGEQLEHIFLVH

YRDVKEEAEVNNHFFLQCIPAVNLWNVLMYAWSKMDNPKNTFDLLRSWEIIGRRNSEEDW

WILIPAYIWWTLQKERNARCFEGKVNSIQKIRVGASRLQPVHPGPLLENPECSSTPCFES

VCRTVQESHTSSPSSVDWKEQGLSSELHSADSRGLVFSRDSFQLNPQVRAFMRTDVYRSS

RNLNVMIQQKFYSAHLNVADLLSSKLTYARLNAGKAVANSRNRLTVTSGEGVEENIHVAP

AQIQNISSSQTVVTLDTAVQTSSLEGGHYSEEAVSLKKLDSFGRCMDREIGGDRDKSLMS

SDSGNCLNILDTDNGDKEVSCLSRHMQLDTNFLGPSVSQEQLFSISEFAPDWAYSGGETK

VFIIGTFLDHEKHFTCQKWSCMFGEDEVSAEVLTQGIIRCQVPSHSPGRVPFYVTCSNRL

PCSEVQEFEYREKPSELALALRPSDEVRLQVQLAKFLYSGLNKKILDCSSGECEKCKLKT

QLCSLKGKTRNAFERLEDLLAIVECDHINFRDVQVQNFMKDKLYDWLVSRAHEEDKGPNI

LDDKGQGVIHLMAALGYEWGLHPLIATGISPNFRDGHGRTALHWAAHYGREDTVIALIKL

GVAASAVDDPTAAFPWGRTAADLASSRGHKGIAGYLAKLDLATHQQSLATNNNALDNIGA

GLEAEKAFESTVQEFVPLNGTIDDDISLKSALACLRKSAHDAALIQASFRARSFHLRQLT

ECRNDVSEASSDLAALGSLNKVQRVSHFEDYLLSAAIKIQQKYHGWEGRKDFLKIRNRIV

KIQAHARGYQVRKQCKKFVWSVSILEKVILRWRRKKKGLRGFQPEKTSQKGTLEFDKKDE

YEYLSIGLKQKFAGVEKALARVQSMVRHPEARDQYMRLVAKFESSKLDDGGRSSSPV

>SmCAMTA5

MVLPGGSLFLFDRKVLRYFRKDGHSWRKKRDGKTVKEAHERLKAGSIDVLHCYYAHGEEN

ENFQRRSYWMLEEEMSHIVLVHYREVKGNRTNFSRIREPQQVTPDLQETEEDVHSSEVDS

SASTKFYPNDYQVNSQVTETTSLSSAQASEYEDAESVYNQHPTSGFHSFLDAQPSAGDGL

AVPYHPIPFSNDQVQFAGSSGLTFSSIPPGNENRNTANTYMPSRNLDFPSWEAISIDNPV

AYQSLHFQPSGQSGANNLMHEQGNTAMGQMFLNDFKRQEHENHIDGLGNWQPSRLGAKTD

DSKPFILRQQAEPTPS

>SmCAMTA6

MFLFNKRVLRYFRKDGHSWRKKKDGRTVGEAHERLKVGNAEALNCYYAHGEKNSNFQRRS

YWMLDPVYEHIVLVHYRDITEVSLQEPNCTNIPCPDMTLATN

>SpCAMTA1

MKGFQIFMYLLMESKVDLEQILKELHHRWLLPHEVCQILRNHQSFCLTQQLQLKPPAGSI

FLYDRKLLPNFCKDGHHWRKNKDGQTIKEAHEKFKAGSVDVLHCYYVHGEGNKNFQRRSY

WMLEEQLEHIVLVHYRDVKEGYRLGASRLQPVHPGLLLENPDSSSRPCFVFGPAFQKSHT

SSPSLVDLKEQALSSELHSGDSKGLMAFSRSKERFQLNPQVRAFMSSGFRRFERNLNVML

QRKFYSGHFNLADLRSSKLTYAKLYAGKAVANNRSRLAITSGKVFEVNIHVAPPQIQNIS

SSQTVVTPDAAVKTSSLDGGLNSDEVGSRKWMDREFAGGNKSLMSSDSGNYWNTLDTDNG

DKEVSTLSRHLLLEANSVGTSPSQKQLFRIFDFSPQWAFSGVETKVLIVGTFLVHRKYLT

CHKWSCMFGEVEVSAEVQTQSIRCQVPFHAPGHVPFYVTCGNRLACSEVREFEYREKSSE

LALALRPSDEVHLQVQLVKLLYSGLNKKFLDCSSRECEKCKLKTQLCSLKCLTGNATERL

EDLLAVIECDHINFKDVQIQNFMKDKLYEWLVSRAHEEDKGPNILNDQGKGVIHLVAALG

YEWGLLPLIAAGISPNFRDACGRTALHWAAHYGREDMVIALIKLGVAAGAVDDPTTASPG

GRTAADLASSRGYKGIAGYLAESDLTSHHQLLATSKNALDTIGAGLEAEKVYESAVQEIV

PLNGTIDDDVSLKASLASLRKSAHAAALIQAAFRARSFRQRQLTESRNDVSEASLDLVAL

GSLNKVQKVNCFEDYLHSAAINIQQKYCGWKGRREFLKVHNQIVKMQALVRGHEVRKQYK
KFVWAVSILEKGILRWRRKKTGLRGFWPEKTSETGTVEREKEEEYDYLSIGLKQKCAGVE
KALGRVESMVRHPEARDQYMRMVAKFKSCKLDDGGREVNRSSPPV
>SpCAMTA2
MESNRAGQLTGKEIHGFRTLQDLDIPSILEEAKMRWLRPNEIHAILCNYKYFNIFVKPVN
LPTSGTIVLFDRKMLRNFRKDGHNWKKKKDGKTVKEAHEHLKVGNDERIHVYYAHGEDLP
TFVRRCYWLLDKSLEHIVLVHYRETQETRGAPETSVAKSSPATPVNSSSSSDPSDPSGWI
LSEECNSVDEQAYGASQHANLELNRDMTAKTHEQRLLEINTLDWDELLAPNDPNKLMATQ
EVGGRASVGQQSQCEVNGYSLNDGSSSMARAPIASLESFVGQVAGSNAVNFNPLNDMSFR
SGDGQMTSNFQKKESGVMTVGAGDSSDSLNKDGLQTQDSFGRWINYFISDSSGSADELMT
PESSVTIDQSYVMQQTFNITEISPSWALSTEETKILVVGHFPGRQSPLAKSNLFCVCADV
CFTAEFVQSGVYRCVISPQAPGLVNLYLSLDGNTPISQVMTFEFRAPSAHKWTDPLEDQS
NWDEFRVQMRLAHLLFSTSKSLSIFSSKVHQNSLKDAKKFVRKCAYITNNWAYLIKSIEG
RKVPSMHAKDCLFELSLQTKFHEWLLERVIEGCKTSERDEQGQGVIHLCAILGYTWAIYP
FTWSGLSVDYRDKHGWTALHWAAHYGREKMVAALLSAGANPNLVTDPNSENPDGYTAADL
ASKNGFDGLGAYLAEKALVAHFEAMTLAGNVSGSLQTTTEPINPENFTEEELYLKDTLAA
YRTAADAAARIQAAFREQSFKLQTKAVEYVNQETEARNIIAAMKIQHAFRNYESRKKLAA
AARIQYRFRTWKMRKDFLAMRRHAIKIQAVFRGYKERKQYRKIVWSVGVLEKAVLRWRLK
RKGFRGLQVQSSESVDIKPDGEVEDFFRASRKQAEERVERSVVRVQAMFRSKRAQEEYSR
MKMEHNNASLEYKRLINPDN
>SpCAMTA3
MEDCGSDPPGFRLDITQILSEVQHRWLRPAEICEILRNHRKFHLTPEAPFRPVSGSVFLF
DRKVLRYFRKDGHNWRKKKDGKTVKEAHEKLKVGSIDVLHCYYAHGEEDDNFQRRSYWML
EQDLMHIVFVHYLEVKGNKVNVSSIRSTKSVHPNYLNDCSLSDSFPTRHKKLTSANADST
SLASTLTEAHEEAESEDSHQACSRFHSYPDRASGMDSHLVENRDTISSSYGSPQSSVEYT
PLPGIDGSGKCDLGNFASGPQRTIDLGSWEPLPQHCSNGEMVCQDDFKNNLSVHGNWQYS
FGQSPLQFHGQNVNQDLIADSSYDLGLPWDLLTVRGPSYLCSNEKEEQLTQLNLQFLKSL
VEVQGDINQENSMDMLELGDYSTIKQPHLSSVKVEEGLKKVDSFSRWVAKELEDVEELHM
QPSNQMSWNVIDTEEEGSCLPSQLHVDSDSLNLSLSQEQVFSIIDFSPNWAYSNLETKIL
ITGRFLKSEGELVEYKWSCMFGEVEVPAEVLADGVLRCHAPPHKPGVLPFYVTCSNRLAC
SEVREFEYRFGPYQEVGAADVSMTEKHLLERIENLLSLGPVSSCRSSDSMEDSEEKRSTV
NKIISMMEEENKQIIERASYGDTSQCRVKEDLYFERKLKQNFYAWLVHQVTDDGRGRTLL
DGEGQGVLHLVAALGYDWAFKPILASGLSVDFRDMNGWTALHWAAFYGREKTVVSLVSLG
ASPGALTDPSAEFPLGRTPADLASANGHKGISGFVAESSLTTHLSKLTVTDAKEELDSEV
CEAKVGETVTERVAVSTTESDVPDVLSLKDSLAAIRNATQAAARIHQIFRVQSFQRKQII
ENCDNELSSDENAIAIVASRACKLGQNNGIAHAAAIQIQKKFRGWNKRKEFLLIRQKIVQ
IQAHIRGHQVRKKYKPIIWSVGILEKVILRWRRKRSGLRGFRSEAVMSKPSTQEDSLPED
DYDFLKEGRKQTEVRMQKALARVKSMTQYSEGRAQYRRLLTAAEGLREVKQDGPIQIPEI
PEDTIYPEEELFDVDSLLDDDTFMSIAFE
>SpCAMTA4
MADSRRYGLNAQLDIEQILLEAQHRWLRPAEICEILKNYQKFRIAPEPPNRPPSGSLFLF
DRKVLRYFRKDGHSWRKKRDGKTVKEAHERLKAGSIDVLHCYYAHGEENENFQRRSYWML
EEEMSHIVLVHYREVKGNRTNFSRIREPLQVTPDLQETDEDVHSSEVDSSASTKFYPNDY
QVNSQVTDTTSFSSAQASEYEDAESVYNQHPTSGFHSFLDAQPSAGDGLAVPYHPIPFSN

DQVQFAGSSGTSFSSIPPGNGNTNTANTYVPSRNLDFASWGTISVNNPAAYQSLHFQPSA

QSSANNMMHEQGNTTMGQLFSNDFTRQEHENHIDGLGNWQTSEVDSSFISKWSMDQKLNP

DLTSGQTIGSSGVYGVERHNSLEASQLLSAQQDKHPMQNELQSQLSDANIGGSLNADLDH

NLSLGVKTDYSALKQPLLDGVLKREGLKKLDSFDRWISKELGDVSESHMQSNSSSYWDNV

GDEDGVDNSTIASQVQLDTYVLSPSLAQDQIFSIIDFSPNWAFSGSEIKVLITGRFLKSQ

QEVENCSWACMFGELEVPAEVIADGVLRCHTPVQKAGRVPFYITCSNRLACSEVREFEFR

VTEGQDVDVANPNSCSSSESLLHMRFGKLLSLESFVSQTSPPISEDDVSHISSKINSLLR

DDDNEWEEMLHLTSENNFMAEKVKDQLLQKLLKEKLRVWLLQKVAEGGKGPNILDEGGQG

VLHFAAALGYDWAVPPTIAAGVSVNFRDVNGWTALHWAASYGRERTVGFLISLGAAAGAL

TDPTPKHPSGRTPADLASSNGHKGIAGYLAESSLSSHLSSLELKEKKQGENEQAFGEAVQ

TVSERTATPAWDGDWSHGVSLKDSLAAVRNATQAAARIHQVFRVQSFQRKQLKEYGGSEF

GLSDERALSLLAMKTNRAGQHDEPHAAAVRIQNKFRSWKGRRDFLLIRQRIIKIQAHVRG

HQVRNKYKNIIWSVGILEKVILRWRRKGSGLRGFKPEAPTEGSNMQDQPVQEDDYDFLKE

GRKQTEERLQKALERVKSMVQYPEARDQYRRLLNVVSDMQEPNSTAASYNSAEAVDFNDD

LIDLGDLLDDDTFMPTAP

>SpCAMTA5

MAESGYDINDLVREAQIRWLKPAEVLFILRNHENHQLSSEPSQKPPSGSLFLYNKRVLRF

FRKDGHSWRKKKDGRTVGEAHERLKVGNAEALNCYYAHGEQNPNFQRRSYWMLDPAYDHI

VLVHYRDIIEGRQNPAFMSESSPISSAFSPSPSSYSTPHTGSTGIASESYEQYQNQSSPG

EICSDAIINNNGMSDTIGRTEEVISSPGLEMSLALRRLEEQLSLNDDSLKEIDPLYGDAI

NDDSSLIQMQGNSNRLLLQHHSGESSESHHQDLTQDAHVWKDMLDHYGVSAAAESQTKYL

HKLDENAMLQTLSERRAIEAYESYKWRDFSDKEAQTAPVQAFKQLEDFKYPTYPSDITTF

GSNPDEYTTIFDQDQIGTSLEDEMSLTIAQKQKFTIRHISPDWGYSSEPTKIVIIGSFLC

NPSECTWTCMFGDIEVPVRIIQEGVICCQAPRHLPGKVTLCVTSGNRESCSEVREFEYRV

KPDDCARNNQPDVEGAYRSTDELLLLVRFVQLLLSDLSVQKGESSELGNDLLEKSKASED

SWSHIIESLLFGTSVPMVTIDWLLQELLKDKFQQWLCSKLQQKDNQIDCSLSKKEQGIIH

MVAGLGFEWALHPILNAGVSANFRDINGWTALHWAARFGREKMVASLIASGASAGAVTDP

SSRDPVGKTAASIASSCGHKGLAGYLSEVALTSHLSSLTLEESELSKGTADVEAERTISS

ISNTSATINEDQRSLKDTLAAVRNAAQAAARIQSAFRAHSFRKRQQREFGVSATTSGDEY

GILSNDIQGLSAASKLAFRNPREYNSAALAIQKKYRGWKGRKDFLAFRQKVVKIQAHVRG

YQVRKQYKVCWAVGILEKVVLRWRRRGVGLRGFRHDTESIDEIEDEDILKVFRKQKVDAA

LDEAVSRVLSMVESPGARQQYHRILEKYRQSKAELEGADSETASTAHGDMSNMENDDIYQ

FPSY

>SpCAMTA6

MAESGYNTNDLVQEGRFRWLRPAEVLFILQNHDDRQLAHQPPQKPASGSMFLFNKRVLRY

FRKDGHSWRKKKDGRTVGEAHERLKVGNAEALNCYYAHGEKNSNFQRRSYWILDPAYEHI

VLVHYRDITEGRQIAAFMSQSSPISSTFPLSPSLYSTQHPGFNVPGSESYQQYQDESRPG

YGEICSDAVIHSNGMNVSDITRMMEGVSNSPKVEISQALRRLEEQLNLNDDSSSEIYSLY

SEIENSNDAENVVHDKSSLVQIQDNSNNFLFLPHSGESSESRDQLLNLDDSMWKEMLDHC

RSSPASQPQAKCFEKLDENGMLQTSSGSEPIEAIKSDRWPIIGGKEALKCSVTNLKQVDD

FKYIGCAQINAFGSYPDQCTTIFDQDQIGISSETNMSLTIVQKQKFTIHDISPDWGYASD

ATKVVIIGSYLCNPSEYTWTCMFGDTEVPVQIIKDGAIRCQAPPHLPGKVALCVTTGNRI

PCSEVREFEYRAKFDDRGQNVVPEVGGASKSSEELLLLVRFVQMLLSDSSVQIGDGSESN

NDILEKSKASEDSWSQVIGSLLFGTSTSTVTIDWLLQELLKNKLQQWLSSKLQVKNNEMV

YSLSRKDQGIVHMIAGLGFEWALHPVLNAGVSANFRDIRGWTALHWAARFGREKMVASLI

ASGAFAGAVTDPSSQDPFGKTAASIASSCGHKGVAGYLSEVALTSHLTSLTLEECDVSKG

TADIEAEQTISNITTTSPVTHEDQLSLKDTLDAVRNAAQAAARIQSAFRAHSFRKRRLRE

AAHVATTCRDEYCILSNDVLGLSAASKLAFRNVRDYNSAALSIQRKYRGWKGRKDFLVFR

QKVVKIQAHVRGYQVRKEYKVCWAVGILEKVVLRWRRRGVGLRGFRLEDEPIEESENEDI

LKLFRKQKVDAAINEAVSRVLLMVDSPEARQQYRRILEKYRQAKAEVAGAKSDAISTAHS

DISNVENNDVYHS

>SpCAMTA7

MESSVSGRLLGCEIHGFRTMQDLDIPNIMEESKMRWLRPNEIHAILCNHKYFNINVKPVN

LPKSGTIVLFDRKMLRNFRRDGYNWKKKKDGKTVKEAHEHLKVGNDERIHVYYAHGEDNT

TFVRRCYWLLDKTLEHVVLVHYRETQEVSSNSTVAQGSPAAPVSSGSALSDPADLSASWV

LSGELDSAVDQQYSASRHAHLEPNRDMTVQNHEQRLLEINTLEWDDLLAPGDPNKMVATQ

QAGGKTAYVQHTSYEQRNLCELNGYSLDGGVSSSLERISTFNNSNEITFQTVDGQMTSSF

EKNESGVMTVSTGDSLDSLNQDRLQTQDSFGRWMNYLIKDSPESIDDPTPESSVSTGQSY

ASEQIFNITEILPAWAPSTEETKICVIGQFHGEQSHLESSSLHCVCGDACFPAEVLQPGV

YRCIVSPQTPGLVNIYLSFDGNKPISQVMSFEFRAPSVHVWTEPPENKSDWDEFRNQMRL

AHLLFSTSKSLNILSSKIHQDLLKDAKKFAGKCSHIIDDWACLIKSIEDKKVSVPRAKDC

LFELSLKTRLQEWLLERVVEGCKISEHDEQGQGVIHLCAILGYTWAVYPFSWSGLSLDYR

DKYGWTALHWAAYYGREKMVATLLSAGAKPNLVTDPTSENLGGCTASDLASKNGHEGLGA

YLAEKALVAQFKDMTLAGNISGSLQTTTESINPGNFTEEELNLKDSLAAYRTAADAAARI

QAAFRERALKVRTKAVESSNPEMEARNIIAAMKIQHAFRNYEMQKQLAAAARIQYRFRTW

KMRKEFLHMRRQAIKIQAVFRGFQVRRHYRKIIWSVGVLEKALFRWRLKRKGLRGLKLQS

SQVIKPDDVEEDFFQASRKQAEERIERSVVRVQAMFRSKQAQEQYRRMKLEHDKATLEYE

GTLNPDTEMD

>LcCAMTA1

MAESGYNVNDLVREGRFRWLRPAEVLFILQNHEDRQLAHQPPQKPASGSMFLFNKRVLRYFRKDGHNWRK

KKDGRAVGEAHERLKVGNAEALNCYYAHGEKNPNFQRRSYWMLDPAYEHIVLVHYRDITEGKQIAAFMSQ

SSPVSSTFSLSPSLSSTQHPGFTVVGSESCQQYQNESSPGYGEICSDAVINSNGMNVSDITGRTEGVSSL

PRVEISQALRRLEEQLSLNDDSSGEIYPLYSEIENSNDAENLVHDKNSLFQIQDNSNNLLLQPHSGESSE

SQDQLLNLDDNMWKEMLDHCRSSPAAESQAKCFGKWDENGMLQTSSGSVPIEATESDRWPKFGGKEALES

SLTNLKQVDDFKYPARAQISTFGSYPDQYTTIFDQDQIGTSFEADMSLTIVQKQKFTIHDISPDWGYSSE

TTKVVIVGSYLCNPSDYTWTCMFGDIEVPVQIIKEGAIRCQAPPHLPGKVALCITTGNRVSCSEVREFEY

RVKLDDRGQSILPEVGGASKSSEELLLLVRFVQMLLSDSSVQKGDGSESNNDILEKSKASEDSWSQVIES

LFFGTSASMITVDWLLQELLKDKLQQWLSSKLQEKDNQIGYSLSRKEQGIIHMIAGLGFEWALHPILNAG

VSVNFRDISGWTALHWAARFGREKMVASLIASGAFAGAVTDPSSKDPFGKTAASIASSCGHKGVAGYLSE

VALTSHLKSLTLEESELSKGTADVEAEKTISSVSTTSPVTHEDQLSLKDTLDAVRNAAQAAARIQSAFRA

HSFRKRRLREAACAATTCGDEYWILSNDALGLSAASKLAFRNTRDYNSAALSIQKKYRGWKCRKDFLAFR

QKVVKIQAHVRGYQVRKEYKVCWAVGILEKVVLRWRRRGVGLRGFRLEEEPIEDGEDEDILKLFRKKKVD

AAINEAVSRVLSMVDSPEARRQYHRILEKYRQAKAG

>LcCAMTA2

MFLFNKRVLRYFRKDGHNWRKKKDGRAVGEAHERLKVGNAEALNCYYAHGEKNPNFQRRSYWMLDPAYEH

IVLVHYRDITEGKQIAAFMSQSSPVSSTFSLSPSLSSTQHPGFTVVGSESCQQYQNESSPGYGEICSDAV

INSNGMNVSDITGRTEGVSSLPRVEISQALRRLEEQLSLNDDSSGEIYPLYSEIENSNDAENLVHDKNSL

FQIQDNSNNLLLQPHSGESSESQDQLLNLDDNMWKEMLDHCRSSPAAESQAKCFGKWDENGMLQTSSGSV

PIEATESDRWPKFGGKEALESSLTNLKQVDDFKYPARAQISTFGSYPDQYTTIFDQDQIGTSFEADMSLT
IVQKQKFTIHDISPDWGYSSETTKVVIVGSYLCNPSDYTWTCMFGDIEVPVQIIKEGAIRCQAPPHLPGK
VALCITTGNRVSCSEVREFEYRVKLDDRGQSILPEVGGASKSSEELLLLVRFVQMLLSDSSVQKGDGSES
NNDILEKSKASEDSWSQVIESLFFGTSASMITVDWLLQELLKDKLQQWLSSKLQEKDNQIGYSLSRKEQG
IIHMIAGLGFEWALHPILNAGVSVNFRDISGWTALHWAARFGREKMVASLIASGAFAGAVTDPSSKDPFG
KTAASIASSCGHKGVAGYLSEVALTSHLKSLTLEESELSKGTADVEAEKTISSVSTTSPVTHEDQLSLKD
TLDAVRNAAQAAARIQSAFRAHSFRKRRLREAACAATTCGDEYWILSNDALGLSAASKLAFRNTRDYNSA
ALSIQKKYRGWKCRKDFLAFRQKVVKIQAHVRGYQVRKEYKVCWAVGILEKVVLRWRRRGVGLRGFRLEE
EPIEDGEDEDILKLFRKKKVDAAINEAVSRVLSMVDSPEARRQYHRILEKYRQAKEIMSSNLLRQVELGG
VKSDTISTAHSDISNAENNDVYTVRRDANKTTLRSPPLVDEFLNVFNASVWF

>LcCAMTA3

MAESGYNVNDLVREGRFRWLRPAEVLFILQNHEDRQLAHQPPQKPASGSMFLFNKRVLRYFRKDGHNWRK
KKDGRAVGEAHERLKGKQIAAFMSQSSPVSSTFSLSPSLSSTQHPGFTVVGSESCQQYQNESSPGYGEIC
SDAVINSNGMNVSDITGRTEGVSSLPRVEISQALRRLEEQLSLNDDSSGEIYPLYSEIENSNDAENLVHD
KNSLFQIQDNSNNLLLQPHSGESSESQDQLLNLDDNMWKEMLDHCRSSPAAESQAKCFGKWDENGMLQTS
SGSVPIEATESDRWPKFGGKEALESSLTNLKQVDDFKYPARAQISTFGSYPDQYTTIFDQDQIGTSFEAD
MSLTIVQKQKFTIHDISPDWGYSSETTKVVIVGSYLCNPSDYTWTCMFGDIEVPVQIIKEGAIRCQAPPH
LPGKVALCITTGNRVSCSEVREFEYRVKLDDRGQSILPEVGGASKSSEELLLLVRFVQMLLSDSSVQKGD
GSESNNDILEKSKASEDSWSQVIESLFFGTSASMITVDWLLQELLKDKLQQWLSSKLQEKDNQIGYSLSR
KEQGIIHMIAGLGFEWALHPILNAGVSVNFRDISGWTALHWAARFGREKMVASLIASGAFAGAVTDPSSK
DPFGKTAASIASSCGHKGVAGYLSEVALTSHLKSLTLEESELSKGTADVEAEKTISSVSTTSPVTHEDQL
SLKDTLDAVRNAAQAAARIQSAFRAHSFRKRRLREAACAATTCGDEYWILSNDALGLSAASKLAFRNTRD
YNSAALSIQKKYRGWKCRKDFLAFRQKVVKIQAHVRGYQVRKEYKVCWAVGILEKVVLRWRRRGVGLRGF
RLEEEPIEDGEDEDILKLFRKKKVDAAINEAVSRVLSMVDSPEARRQYHRILEKYRQAKEIMSSNLLRQV
ELGGVKSDTISTAHSDISNAENNDVYTVRRDANKTTLRSPPLVDEFLNVFNASVWF

>LcCAMTA4

MAESGYNVNDLVREGRFRWLRPAEVLFILQNHEDRQLAHQPPQKPASGSMFLFNKRVLRYFRKDGHNWRK
KKDGRAVGEAHERLKVGNAEALNCYYAHGEKNPNFQRRSYWMLDPAYEHIVLVHYRDITEGKQIAAFMSQ
SSPVSSTFSLSPSLSSTQHPGFTVVGSESCQQYQNESSPGYGEICSDAVINSNGMNVSDITGRTEGVSSL
PRVEISQALRRLEEQLSLNDDSSGEIYPLYSEIENSNDAENLVHDKNSLFQIQDNSNNLLLQPHSGESSE
SQDQLLNLDDNMWKEMLDHCRSSPAAESQAKCFGKWDENGMLQTSSGSVPIEATESDRWPKFGGKEALES
SLTNLKQVDDFKYPARAQISTFGSYPDQYTTIFDQDQIGTSFEADMSLTIVQKQKFTIHDISPDWGYSSE
TTKVVIVGSYLCNPSDYTWTCMFGDIEVPVQIIKEGAIRCQAPPHLPGKVALCITTGNRVSCSEVREFEY
RVKLDDRGQSILPEVGGASKSSEELLLLVRFVQMLLSDSSVQKGDGSESNNDILEKSKASEDSWSQVIES
LFFGTSASMITVDWLLQELLKDKLQQWLSSKLQEKDNQIGYSLSRKEQGIIHMIAGLGFEWALHPILNAG
VSVNFRDISGWTALHWAARFGREKMVASLIASGAFAGAVTDPSSKDPFGKTAASIASSCGHKGVAGYLSE
VALTSHLKSLTLEESELSKGTADVEAEKTISSVSTTSPVTHEDQLSLKDTLDAVRNAAQAAARIQSAFRA
HSFRKRRLREAACAATTCGDEYWILSNDALGLSAASKLAFRNTRDYNSAALSIQKKYRGWKCRKDFLAFR
QKVVKIQAHVRGYQVRKEYKVCWAVGILEKVVLRWRRRGVGLRGFRLEEEPIEDGEDEDILKLFRKKKVD
AAINEAVSRVLSMVDSPEARRQYHRILEKYRQAKVELGGVKSDTISTAHSDISNAENNDVYTVRRDANKT
TLRSPPLVDEFLNVFNASVWF

>LcCAMTA5

MAESGYNVNDLVREGRFRWLRPAEVLFILQNHEDRQLAHQPPQKPASGSMFLFNKRVLRYFRKDGHNWRK
KKDGRAVGEAHERLKVGNAEALNCYYAHGEKNPNFQRRSYWMLDPAYEHIVLVHYRDITEIAAFMSQSSP

VSSTFSLSPSLSSTQHPGFTVVGSESCQQYQNESSPGYGEICSDAVINSNGMNVSDITGRTEGVSSLPRV
EISQALRRLEEQLSLNDDSSGEIYPLYSEIENSNDAENLVHDKNSLFQIQDNSNNLLLQPHSGESSESQD
QLLNLDDNMWKEMLDHCRSSPAAESQAKCFGKWDENGMLQTSSGSVPIEATESDRWPKFGGKEALESSLT
NLKQVDDFKYPARAQISTFGSYPDQYTTIFDQDQIGTSFEADMSLTIVQKQKFTIHDISPDWGYSSETTK
VVIVGSYLCNPSDYTWTCMFGDIEVPVQIIKEGAIRCQAPPHLPGKVALCITTGNRVSCSEVREFEYRVK
LDDRGQSILPEVGGASKSSEELLLLVRFVQMLLSDSSVQKGDGSESNNDILEKSKASEDSWSQVIESLFF
GTSASMITVDWLLQELLKDKLQQWLSSKLQEKDNQIGYSLSRKEQGIIHMIAGLGFEWALHPILNAGVSV
NFRDISGWTALHWAARFGREKMVASLIASGAFAGAVTDPSSKDPFGKTAASIASSCGHKGVAGYLSEVAL
TSHLKSLTLEESELSKGTADVEAEKTISSVSTTSPVTHEDQLSLKDTLDAVRNAAQAAARIQSAFRAHSF
RKRRLREAACAATTCGDEYWILSNDALGLSAASKLAFRNTRDYNSAALSIQKKYRGWKCRKDFLAFRQKV
VKIQAHVRGYQVRKEYKVCWAVGILEKVVLRWRRRGVGLRGFRLEEEPIEDGEDEDILKLFRKKKVDAAI
NEAVSRVLSMVDSPEARRQYHRILEKYRQAKEIMSSNLLRQVELGGVKSDTISTAHSDISNAENNDVYTV
RRDANKTTLRSPPLVDEFLNVFNASVWF

>LcCAMTA6

MAESGYNVNDLVREGRFRWLRPAEVLFILQNHEDRQLAHQPPQKPASGSMFLFNKRVLRYFRKDGHNWRK
KKDGRAVGEAHERLKVGNAEALNCYYAHGEKNPNFQRRSYWMLDPAYEHIVLVHYRDITEGKQIAAFMSQ
SSPVSSTFSLSPSLSSTQHPGFTVVGSESCQQYQNESSPGYGEICSDAVINSNGMNVSDITGRTEGVSSL
PRVEISQALRRLEEQLSLNDDSSGEIYPLYSEIENSNDAENLVHDKNSLFQIQDNSNNLLLQPHSGESSE
SQDQLLNLDDNMWKEMLDHCRSSPAAESQAKCFGKWDENGMLQTSSGSVPIEATESDRWPKFGGKEALES
SLTNLKQVDDFKYPARAQISTFGSYPDQYTTIFDQDQIGTSFEADMSLTIVQKQKFTIHDISPDWGYSSE
TTKVVIVGSYLCNPSDYTWTCMFGDIEVPVQIIKEGAIRCQAPPHLPGKVALCITTGNRVSCSEVREFEY
RVKLDDRGQSILPEVGGASKSSEELLLLVRFVQMLLSDSSVQKGDGSESNNDILEKSKASEDSWSQVIES
LFFGTSASMITVDWLLQELLKDKLQQWLSSKLQEKDNQIGYSLSRKEQGIIHMIAGLGFEWALHPILNAG
VSVNFRDISGWTALHWAARFGREKMVASLIASGAFAGAVTDPSSKDPFGKTAASIASSCGHKGVAGYLSE
VALTSHLKSLTLEESELSKGTADVEAEKTISSVSTTSPVTHEDQLSLKDTLDAVRNAAQAAARIQSAFRA
HSFRKRRLREAACAATTCGDEYWILSNDALGLSAASKLAFRNTRDYNSAALSIQKKYRGWKCRKDFLAFR
QKVVKIQAHVRGYQVRKEYKVCWAVGILEKVVLRWRRRGVGLRGFRLEEEPIEDGEDEDILKLFRKKKVD
AAINEAVSRVLSMVDSPEARRQYHRILEKYRQAKEIMSSNLLRQVELGGVKSDTISTAHSDISNAENNDV
YTVRRDANKTTLRSPPLVDEFLNVFNASVWF

>CaCAMTA1

MADCGSDPSGFRLDITQILSEVQHRWLRPAEICEILRNYRKFHLTPEAPYRPVSGSVFLF
DRKVLRYFRKDGHNWRKKKDGKTVKEAHEKLKVGSIDVLHCYYAHGEEDDNFQRRSYWML
EQDLMHIVFVHYLEVKGNKVNVGYIRSVKFAHSNYQNECSLSDSMPTGHKKLASANADSA
SLASTLTEAHEEAESEDNHHACSRFHSYPDRASGMDSHLVESRDTICSSYGSPQSSVEYT
SLPSIDGAGKCDLGNFASGPQRTVDLGYGFPVSQHCSNGEMVGQDDFKNNLSVHENWQCS
FGDSPLQFHGQNVNQDLIADLSYGLGNSFQNRSLPSDLLSVRGQSYLYPDAQEGQLTQLD
LQYLNSLLEVQGDMNQESNMDMIELGDYSTVKQPQLSSVKMEEGLKKVDSFSRWVAKELE
DVEELHMQPNNRISWNAIDTEEEDSYLPNRLHMDSDSLNPSLSQEQVFSIIDFSPNWAYS
NFETKVLITGRFLKSEGELVEYKWSCMFGEVEVPAEVLADGVLRCHAPPHKPGVLPFYVT
CSNRLACSEVREFEYRFGPFQEFGAANVSTTEMHLLERIENLLSLGPVSNCRISDTMEAA
KDKQSTVNKIIFMMEEENQQMIERASDYDTSQCRVKEDLFLESKLKQNFYAWLIHQVTDD
GRGRTLLDDEGQGILHLVAALGYDWALKPILASGVSVDFRDINGWTALHWAAFYGREKTV
VGLVSLGASPGALTDPSAEFPLARTPADLASANGHKGISGFLAESSLTTHLTKLTVSDAK
EELASEGCEAKVGETVTERVAVTTTGNDVPDVLSLKDSLAAIRNATQAAARIHQIFRVQS

FQRKQIIEHSDDELSSDENALSILASKSCKLGQNNGIAHAAAIQIQKKFRGWNKRKEFLL
IRQKIVKIQAHIRGHQVRKKYKPIIWSVGILEKVILRWRRKRSGLRGFKSEEVMNKPSTQ
DDSLPEDDYDFLKEGRKQTEVRMQKALARVKSMTQYPEGRAQYRRLLTAAEGLREVKV
>CaCAMTA2
YDINDLVREAQIRWLKPAEVLFILRNHENHQLSSGSLFLFNKRVLRFFRKDGHSWRKKKD
GRTVGEAHERLKVGNAEALNCYYAHGEQNPNFQRRSYWMLDPAYDHIVLVHYRDITEGRQ
NPVFMSESSPISSAFSPSPRSYSTQHTGSTVIASESYELYQNQSSPGEICSDAVINNSGT
SDTTGRTEEPISSPGLEMSQALRRLEEQLSLNDDSFKEIDPLYADAINDDSSFIQMQGNS
NGLLLQHHLGLSFSYINIFVECILTCLFLARYVYDNAIHFNFVQRTANTFITSRISLPPY
SPPHHCWDNFPFAFVCTAITLIVPAFQSLTRTSRHSVTGCFFIKFKHATIYSESSESHHQ
DLTQDSDMWKDMLDDYGVSAAAESQTKYLHKLDENAMLQISSERCAIETYGSYKWPCFSD
KEAPTAQVPDFKQLEDFKYPTYPPDINTFGSNPDKYTTLFDQDHIGTSLEDEVSLTIAQK
QKFTIRDISPDWGYSSEATKVVIIGSFLCNPSECTWACMFGDIEVPVQIIQEGVICCQAP
RHLPGKVTLCVTSGNRESCSEVREFEYRVEPDDCARNNQPDVVGAYQSSEELLLLVRFVQ
LLLSDLSVQKGDSSELANDFLEKSKANEDSWSQVIESLLFGTSTSMITIDWLLQELLKDK
FQQWLYCKLQQKDNQIGCSLSKKEQGIIHMVAGLGYEWALHPILNAGLSVNFRDINGWTA
LHWAARFGREKMVASLIASGASAGAVTDPSSRDPVGKTAASIASSCGHKGLAGYLSEVAL
TSHLSSLTLEQSELSKGTADVEAERTISSISNTSATINEDQRSLKDTLAAVRNAAQAAAR
IQSAFRAHSFRKRQLRESVVSATTSGDEYGVLSNDIHGLSAASKLAFRNTRDYNSAALAI
QKKYRGWKGRKDFLAFRQKVVKIQAHVRGYQVRKQYKVCWAVGILEKVVLRWRRRGVGLR
GFRHDIESIDESEDEDILKVFRKQKVDAALDEAVSRVLSMVESPGARQQYHRILEKYRQA
KVS
>CaCAMTA3
MKSPSAWTCELVIFGYSQHNGGIEDEVAPTKRDSCNTLQPQVGNEEWIHVYYAHGEDSST
FVRRCYWLLDKTLEHVVLVHYRETIAGFSNNKAAPVSSGSALSGPADMSASWDLSGQPDS
AVDQQYSVSQHAHLEPNSDMTVQNHEQRLLEINTLEWDDLLAPGDSNMIISTQQAGGRTV
SVQHTSYEQHNLCELNGYRLNGVSSSLERMSTVNNGNEVTFQTADGQMTSSFQENESGVM
TDSFGRWMSYLITDPPGSPDDPTPESSASTEILQPVVYRCVVSPQTPGLVNIYLSFDGNK
PISQIMSFEFRAPSVHLSTEPPENKSNWDELALQMRLAHLLFSTSKSLNILSSKIHQELL
KDAKTFAGKCSHIIDNWACLIKSIEDKKLSVACAKDRLFELSLRTSLQEWLMERIVEGCK
ISEHDERGYTWAVYPFSWSGLSLDYRDKYGRTALHWAAYYGREKMVATLLSAGAKPNLVT
DPTSENLGGCTASELASKNGHEGLGAYLAEKAIVAQFKDMALAGNISGSLQTTTESINPG
NFTEEELNLKDSLAAYCTAADSAARIQAAFRERSLKARTKAVESSNQEMEARNIIVAMKI
QHAFRNYEMQKQLAAAARIQYRFRTWKMRKEFLHMRRQAIKIQAVFRDFQVRRHYREIIW
SVGVLEKALFRWHLKKKGFRGLQLPSSQVVNHKPDDMEEDFFQASRKQAEERIERSVVRV
QAMFRSKQTQEQYWRMKLEHNKATVIAILEIDSETNFKTPLRFTYIFFLSQLIYEGTLNP
DTEME
>AtCAMTA1
MARKKKSVSFSHLDIARNEGNKIISFSVHAYSDRLGFVDSLFDYESLRSLLVDFWVYPSMVDRRSFGSITPPLQLDMEQLLSEAQ
HRWLRPTEICEILQNYHKFHIASESPTRPASGSLFLFDRKVLRYFRKDGHNWRKKKDGKTIREAHEKLKVGSIDVLHCYYAHGEA
NENFQRRCYWMLEQYYYRKASSHWVLVATLSLFSFGYLRPSWVRHLMHIVFVHYLEVKGNRTSIGMKENNSNSVNGTASVNID
STASPTSTLSSLCEDADTVLVQGIVNKQVPSYDHLLNLKLEIAMVGHLLLACVMFHRFMGTESEKMQPSNTDSMLVEENSEKGG
RLKAEHIRNPLQTQFNWQDDTDLALFEQSAQDNFETFSSLLGSENLQPFGISYQAPPSNMDSEYMPVMKILRRSEDSLKKVDSFS
KWAIKELGEMEDLQMQSSRGDIAWTTVECETAAAGISLSPSLSEDQRFTIVDFWPKSAKTDAEVEVMVIGTFLLSPQEVTKYNW

SCMFGEVEVPAEILVDGVLCCHAPPHTAGHVPFYVTCSNRFACSEVREFDFLSGSTQKINATDVYGTYTNEASLQLRFEKMLAHR
DFVHEHHIFEDVGDKRRQISKIMLLKEEKEYLLPGTYQRDSTKQEPKGQLFRELFEEELYIWLIHKVTEEGKGPNILDEDGQGILH
FVAALGYDWAIKPVLAAGVNINFRDANGWSALHWAAFSGREETVAVLVSLGADAGALTDPSPELPLGKTAADLAYANGHRGISG
FLAESSLTSYLEKLTVDSKENSPANSCGEKAVQTVSERTAAPMTYGDVPEKLSLKDSLTAVRNATQAADRLHQVFRMQSFQRKQL
CDIGDDEKIDISDQLAVSFAASKTKNPGQGDVSLSCAATHIQKKYRGWKKRKEFLLIRQRIVKIQAHVRGHQVRKQYRTVIWSVG
LLEKIILRWRRKGNGLRGFKRNAVAKTVEPEPPVSAICPRIPQEDEYDYLKEGRKQTEERLQKALTRVKSMVQYPEARDQYRRLL
TVVEGFRENEASSSASINNKEEEAVNCEEDDFIDIESLLNDDTLMMSISP

>AtCAMTA2

MADRGSFGFAPRLDIKQLLSEAQHRWLRPAEICEILRNHQKFHIASEPPNRPPSGSLFLFDRKVLRYFRKDGHNWRKKKDGKTVK
EAHEKLKVGSIDVLHCYYAHGEDNENFQRRCYWMLEQDLMHIVFVHYLEVKGNRMSTSGTKENHSNSLSGTGSVNVDSTATR
SSILSPLCEDADSGDSRQASSSLQQNPEPQTVVPQIMHHQNASTINSYNTTSVLGNRDGWTSAHGNRVKGSNSQRSGDVPAWDA
SFENSLARYQNLPYNAPLTQTQPSTFGLIPMEGKTEKGSLLTSEHLRNPLQSQVNWQTPVQESVPLQKWPMDSHSGMTDATDLA
LFGQGAHENFGTFSSLLGSQDQQSSSFQAPFTNNEAAYIPKLGPEDLIYEASANQTLPLRKALLKKEDSLKKVDSFSRWVSKELG
EMEDLQMQSSSGGIAWTSVECENAAAGSSLSPSLSEDQRFTMIDFWPKWTQTDSEVEVMVIGTFLLSPQEVTSYSWSCMFGEVE
VPADILVDGVLCCHAPPHEVGRVPFYITCSDRFSCSEVREFDFLPGSTRKLNATDIYGANTIETSLHLRFENLLALRCSVQEHHIFE
NVGEKRRKISKIMLLKDEKEPPLPGTIEKDLTELEAKERLIREEFEDKLYLWLIHKVTEEGKGPNILDEDGQGVLHLAAALGYDW
AIKPILAAGVSINFRDANGWSALHWAAFSGREDTVAVLVSLGADAGALADPSPEHPLGKTAADLAYGNGHRGISGFLAESSLTSY
LEKLTVDAKENSSADSSGAKAVLTVAERTATPMSYGDVPETLSMKDSLTAVLNATQAADRLHQVFRMQSFQRKQLSELGGDNKF
DISDELAVSFAAAKTKKSGHSSGAVHAAAVQIQKKYRGWKKRKEFLLIRQRIVKIQAHVRGHQVRKQYRAIIWSVGLLEKIILRW
RRKGSGLRGFKRDTISKPTEPVCPAPQEDDYDFLKEGRKQTEERLQKALTRVKSMAQYPEARAQYRRLLTVVEGFRENEASSSS
ALKNNTEEAANYNEEDDLIDIDSLLDDDTFMSLAFE

>AtCAMTA3

MAEARRFSPVHELDVGQILSEARHRWLRPPEICEILQNYQRFQISTEPPTTPSSGSVFMFDRKVLRYFRKDGHNWRKKKDGKTV
KEAHERLKAGSVDVLHCYYAHGQDNENFQRRSYWLLQEELSHIVFVHYLEVKGSRVSTSFNRMQRTEDAARSPQETGDALTSE
HDGYASCSFNQNDHSNHSQTTDSASVNGFHSPELEDAESAYNQHGSSTAYSHQELQQPATGGNLTGFDPYYQISLTPRDSYQKEL
RTIPVTDSSIMVDKSKTINSPGVTNGLKNRKSIDSQTWEEILGNCGSGVEALPLQPNSEHEVLDQILESSFTMQDFASLQESMVKS
QNQELNSGLTSDRTVWFQGQDMELNAISNLASNEKAPYLSTMKQHLLHGALGEEGLKKMDSFNRWMSKELGDVGVIADANES
FTQSSSRTYWEEVESEDGSNGHNSRRDMDGYVMSPSLSKEQLFSINDFSPSWAYVGCEVVVFVTGKFLKTREETEIGEWSCMFG
QTEVPADVISNGILQCVAPMHEAGRVPFYVTCSNRLACSEVREFEYKVAESQVFDREADDESTIDILEARFVKLLCSKSENTSPVS
GNDSDLSQLSEKISLLLFENDDQLDQMLMNEISQENMKNNLLQEFLKESLHSWLLQKIAEGGKGPSVLDEGGQGVLHFAASLGY
NWALEPTIIAGVSVDFRDVNGWTALHWAAFFGRERIIGSLIALGAAPGTLTDPNPDFPSGSTPSDLAYANGHKGIAGYLSEYALRA
HVSLLSLNDKNAETVEMAPSPSSSSLTDSLTAVRNATQAAARIHQVFRAQSFQKKQLKEFGDKKLGMSEERALSMLAPKTHKSG
RAHSDDSVQAAAIRIQNKFRGYKGRKDYLITRQRIIKIQAHVRGYQFRKNYRKIIWSVGVLEKVILRWRRKGAGLRGFKSEALVE
KMQDGTEKEEDDDFFKQGRKQTEDRLQKALARVKSMVQYPEARDQYRRLLNVVNDIQESKVEKALENSEATCFDDDDDLIDIE
ALLEDDDTLMLPMSSSLWTS

>AtCAMTA4

MSSVAEDNSFTCDIATIFVAICRNPPANPSDSLFQYEISTLYQEAHSRWLKPPEVLFILQNHESLTLTNTAPQRPTSGSLLLFNKRVLK
FFRKDGHQWRRKRDGRAIAEAHERLKVGNAEALNCYYAHGEQDPTFRRRIYWMLDPEYEHIVLVHYRDVSEREEGQQTGGQV
YQFAPILSTQNVSYNQYIGDSSDIYQQSSTSPGVAEVNSNLEGSASSSEFGQALKMLKEQLSIGDEHVNSVDPHYIQPESLDSLQFL
EYSDIDHLAQPTTVYQRPENNKLERCYGGNFGAQYSAKNDSNKLERCYGGYVGGAEYHSSNLMLVKNGSGPSGGTGGSGDQG
SESWKDVLEACEASIPLNSEGSTPSSAKGLLAGLQEDSNWSYSNQVDQSTFLLPQDLGSFQLPASYSALVAPENNGEYCGMMED
GMKIGLPFEQEMRVTGAHNQKFTIQDISPDWGYANETTKVIIIGSFLCDPTESTWSCMFGNAQVPFEIIKEGVIRCEAPQCGPGKV
NLCITSGDGLLCSEIREFEYREKPDTCCPKCSEPQTSDMSTSPNELILLVRFVQTLLSDRSSERKSNLESGNDKLLTKLKADDDQW
RHVIGTIIDGSASSTSTVDWLLQELLKDKLDTWLSSRSCDEDYITCSLSKQEQGIIHMVAGLGFEWAFYPILAHGVNVDFRDIKGW

SALHWAAQFGSEKMVAALIASGASAGAVTDPSRQDPNGKTAASIAASNGHKGLAGYLSEVALTNHLSSLTLEETENSKDTAQVQ

TEKTLNSISEQSPSGNEDQVSLKDTLAAVRNAAQAAARIQAAFRAHSFRKRKQREAALVACLQEYGMYCEDIEGISAMSKLTFG

KGRNYNSAALSIQKNFRGYKDRKCFLELRQKVVKIQAHVRGYQIRKNYKVICWAVRILDKVVLRWRRKGVGLRGFRQDVESTE

DSEDEDILKVFRKQKVDVAVNEAFSRVLSMSNSPEARQQYHRVLKRYCQTKAELGKTETLVGEDDDGLFDIADMEYDTLFSLP

>AtCAMTA5

MAGVDSGKLIGSEIHGFHTLQDLDIQTMLDEAYSRWLRPNEIHALLCNHKFFTINVKPVNLPKSGTIVLFDRKMLRNFRKDGHN

WKKKKDGKTIKEAHEHLKVGNEERIHVYYAHGEDTPTFVRRCYWLLDKSQEHIVLVHYRETHEVHAAPATPGNSYSSSITDHLS

PKIVAEDTSSGVHNTCNTGFEVRSNSLGSRNHEIRLHEINTLDWDELLVPADISNQSHPTEEDMLYFTEQLQTAPRGSVKQGNHLA

GYNGSVDIPSFPGLEDPVYQNNNSCGAGEFSSQHSHCGVDPNLQRRDFSATVTDQPGDALLNNGYGSQDSFGRWVNNFISDSPG

SVDDPSLEAVYTPGQDSSTPPTVFHSHSDIPEQVFNITDVSPAWAYSTEKTKILVTGFFHDSFQHLGRSNLICICGELRVPAEFLQMG

VYRCFLPPQSPGVVNLYLSVDGNKPISQLFSFEHRSVQFIEKAIPQDDQLYKWEEFEFQVRLAHLLFTSSNKISVLTSKISPENLLEA

KKLASRTSHLLNSWAYLMKSIQANEVPFDQARDHLFELTLKNRLKEWLLEKVIENRNTKEYDSKGLGVIHLCAVLGYTWSILLFS

WANISLDFRDKQGWTALHWAAYYGREKMVAALLSXGARPNLVTDPTKEFLGGCTAADLAQQKGYDGLAAFLAEKCLVAQFKD

MQTAGNISGNLETIKAEKSSNPGNANEEEQSLKDTLAAYRTAAEAAARIQGAFREHELKVRSSAVRFASKEEEAKNIIAAMKIQH

AFRNFEVRRKIAAAARIQYRFQTWKMRREFLNMRKKAIRIQAAFRGFQVRRQYQKITWSVGVLEKAILRWRLKRKGFRGLQVS

QPDEKEGSEAVEDFYKTSQKQAEERLERSVVKVQAMFRSKKAQQDYRRMKLAHEEAQLEYDGMQELDQMATEES

>AtCAMTA6

MDGDGLGRLIGSEIHGFHTLQDLDVQTMLEEAKSRWLRPNEIHAILYNPKYFTINVKPVNLPNSGRIILFDRKMLRNFRKDGHNW

KKKKDGRTVKEAHEHLKVGNEERIHVYYAHGEDNTTFVRRCYWLLDKARENIVLVHYRDTQEAATTSGDSISSPISVSEQTFPN

RVAAEDIDTVVRNHDISLHDINTLDWDELLVPTDLNNQSAPTVDNLSYFTEPLQNAANGTAEHGNATVADGSLDALLNDGPQSR

ESFGRWMNSFISESNGSLEDPSFEPMVMPRQDPLAPQAVFHSHSNIPEQVFNITDVSPAWAYSSEKTKILVTGFLHDSYQHLERSNL

YCVCGDFCVPAEYLQAGVYRCIIPPHSPGMVNLYLSADGHKPISQCFRFEHRAVPVLDKTVPEDNQDSKWEEFEFQVRLSHLLFT

SSNKLNVLSSKISPHNLRDAKKLASKTNHLLNSWAYLVKSIQGNKVSFDQAKDHLFELSLKNRLKEWLMEKVLEGRNTLDYDS

KGLGVIHLCASLGYTWSVQLFSLSGLSLNFRDKQGWTALHWAAYYGREKMVAALLSAGARPNLVTDSTKDNLGGCMAADLA

QQNGYDGLAAYLAEKCLVAQFRDMKIAGNITGDLEACKAEMLNQGTLPEDEQSLKDALAAYRTAAEAAARIQGAFREKALKA

ARSSVIQFANKEEEAKSIIAAMKIQNAFRKYDTRRKIEAAYRIQCRFQTWKIRREYLNMRRQAIRIQAAFRGLQARRQYKKILWS

VGVLEKAVLRWRQKRKGFRGLQVAAEEDSPGEAQEDFYKTSQRQAEERLERSVVRVQAMFRSKKAQQDYRRMKLTHEEAQLE

YGCLEDI

>BrCAMTA1 XP_009105129.1

MQSEYEIRTLNQEAQTRWLKPPEVYFILKNHERYELTHKAPHKPTSGSLCLYNKRVLKFFRKDGHHWRKKRDGRAIAEAHERLK

VGNVEALNCYYAHGEHDPSFQRRIFWMLDLEYDHIVLVHYRDVSDAKEGKQSSGTVLQFSLKPSTLFSSPSYSHHIGDSSTDIQQ

QHSEANSGVVFSSNGGEEGSGSSYEFENREAIKRLEVQLSLGDDNNVVQNESLDGLQFLDFSTDLDHLVPPPATIHQRPESSSKLG

RCYGGYVGPQCNVGPPLYSQNQSLDSLLSLEYIKDMNQLAQPEAGQQRQESNRLERSYGGYIGTEYHPNNLMLVNNGSGGSGG

SGESWKDVLEACGASIALNSQGSTPSSAKVLLSGMQEDSNWTYINQADQATLLLPQELDSSLQLPPPCYSELRAPENNGYYNTM

LDDEGQTGPPLEQVMNPTVAYNQKFTIQDISPEWGYANETTKVIIIGSFLCDPTESTWACMFGNAQVPFEIVKEGVIRCQAPPCGP

GKVNLCITSGDGLSCSQIVEFEYRDKPDDRSSPRSSPDVLLLLVKFVQTLLLDRKGNLEPNTDDDEWRRIIDKIRDGTATSSSTVD

WLLQELLKDKLATWLSSRSQDKDQTSCSLSKQEQGIIHMVAGLGFDWALSPILGHGVSIDFRDDNGWSALHWAAQLGSEKMVA

ALIASGASAGAVTDPNTQDPVGKTPASIAASNGHKGLAGYLSEVALTNHLSSLTLEETEDSIESARAEAEIAVKSIAGKSHPVNDDP

QSLAALKNAAEAAARIQAAFRAHSFRKRQQREADMYACLKEYGIYADMSKQNVRNYHSAALSIQKKYRGYKGRKEFLTKRQK

AIMIQAHVRGYQTRKQYKVICWAVGMLDKVILRWRRKGVGLKGFRQDVESGEDSEDEDILRVFRKQKVEGAVNEAFSRVMSM

TRTPEARQQYHRVLTTYCKKKAELGKTETLGIGGSGGGFEDDDALFDIADMEDDHLFSLP

>BrCAMTA2 XP_009105273.1

MQSEYDVSTLYREAHTRWLKPPEVHFILLNHERYRLTDKPPHKPSSGSVLLYNKRVLKFFRKDGHQWKRKKDGRAIAEAHERLK

VGNVEALQCYYAHGEHEPSFQRRIYWILDPEYEHIALVHYRDVSDGKEVKQQTGGTVLHFSPNPSTLGSIGTQNASYSHCIGVSS

EIHQQQHSSASAGNVEVNSDVFCNSNGTETSKGSGSLSYEFETREALKRLEEQLSLGDDDNVVQNESLDGLQFLGFSKTDIDHHL
VPPATVHQRPESSSKLGRCYGGYVGGAQCNVSTVGSPLHSLNSLLSLECTEDINAQPAAGHQRAENNRLERCYGGYIGAEYHSN
NLMLVKNDSGGSGGSGDQKAESWKDVLEACEASIALNSEQGSTPSSAKGLLTRMQEDSNLSYSNQADQATLLLPQELGPSFELP
TRYSELGALANNANNSRMELPFEQVMNQTVAHKQKFTIQDISPEWGYANETTKVMIIGSFLCDPKESTWSCMFGSTEVPFEIIKE
GVIRCQAPPCGPGKVNLCIASGDGLSCSQIKEFEYRDKPDDTSCPWSSRDELLLLVRLVQTLVSDSKSNLEPWSHILETVLDGTAT
SSSTVDWLLQELVKEKLDAWLSSRPRQVEDQTSCCSLSKQEQGIIHMVAGLGFEWALHPILSRGVSVDFRDINGWSALHWAARF
GSEKMVAALIASGASAGAVTDPTAQDPAGKTAASIAASNGHKGLAGYLSEVALTNHLSSLTLEETEKSKETAQLQAEVTLNSISER
SPHSLKDSLAAVRNAAQAVARIQAAFRAHSFRKRQQREAAMAAYFQEYGIYADIKGISAISKVAPGNVKNYHSAALSIQKNYRRY
KRRKEFLSLRKKVVKIQAHVRGYQIRKHYKVICWAVGILDKVVLRWRRKGAGLRGFRQDVEDSEEEDILKVFRKQKVDVAVKE
AFSRVLSMAKSPEARQQYHRVLKRYCQTKAELGKTETLGAGGDEDEDLFDLADMEDDSLFAL

>BrCAMTA3 XP_009105274.1

MQSEYDVSTLYREAHTRWLKPPEVHFILLNHERYRLTDKPPHKPSSGSVLLYNKRVLKFFRKDGHQWKRKKDGRAIAEAHERLK
VGNVEALQCYYAHGEHEPSFQRRIYWILDPEYEHIALVHYRDVSDGKEVKQQTGGTVLHFSPNPSTLGSIGTQNASYSHCIGVSS
EIHQQQHSSASAGNVEVNSDVFCNSNGTETSKGSGSLSYEFETREALKRLEEQLSLGDDDNVVQNESLDGLQFLGFSKTDIDHHL
VPPATVHQRPESSSKLGRCYGGYVGGAQCNVSTVGSPLHSLNSLLSLECTEDINAQPAAGHQRAENNRLERCYGGYIGAEYHSN
NLMLVKNDSGGSGGSGDQKAESWKDVLEACEASIALNSEGSTPSSAKGLLTRMQEDSNLSYSNQADQATLLLPQELGPSFELPT
RYSELGALANNANNSRMELPFEQVMNQTVAHKQKFTIQDISPEWGYANETTKVMIIGSFLCDPKESTWSCMFGSTEVPFEIIKEG
VIRCQAPPCGPGKVNLCIASGDGLSCSQIKEFEYRDKPDDTSCPWSSRDELLLLVRLVQTLVSDSKSNLEPWSHILETVLDGTATSS
STVDWLLQELVKEKLDAWLSSRPRQVEDQTSCCSLSKQEQGIIHMVAGLGFEWALHPILSRGVSVDFRDINGWSALHWAARFGS
EKMVAALIASGASAGAVTDPTAQDPAGKTAASIAASNGHKGLAGYLSEVALTNHLSSLTLEETEKSKETAQLQAEVTLNSISERSP
HSLKDSLAAVRNAAQAVARIQAAFRAHSFRKRQQREAAMAAYFQEYGIYADIKGISAISKVAPGNVKNYHSAALSIQKNYRRYK
RRKEFLSLRKKVVKIQAHVRGYQIRKHYKVICWAVGILDKVVLRWRRKGAGLRGFRQDVEDSEEEDILKVFRKQKVDVAVKEA
FSRVLSMAKSPEARQQYHRVLKRYCQTKAELGKTETLGAGGDEDEDLFDLADMEDDSLFAL

>BrCAMTA4 XP_009107886.1

MAGVDSGRLIGSEIHGFHTLQDLDIRTMLEEAYTRWLRPNEIHALLSNHNYFTINVKPVHLPKSGTIVFFDRKMLRNFRKDGHN
WKKKKDGKTIKEAHEHLKVGNEERIHVYYAHGDDNPTFVRRCYWLLDKSQEHIVLVHYRETHEVQAAPATPGNSYSSSTTDHV
SAKPVTEDINSGVRNACNTARSNSLVARNHEISLHEINTLDWDELLVETGMTNQSSPTQDDVLYFTEQLQTAAMGSAQQGNHHA
VYNGSTDIPSYLGLGDPVYQNNSPCGAREFSSQHLHCVVDPNQQTRDSSATVADEQGDALLNNGYGSQESFGKWVNNFISDSPG
SVDDPSLEAVYTPGQESSAPPAVVHSQSNIPEQVFNITDVSPAWAYSTEKTKILVTGFFHDSFQHFGRSNLFCICGELRVPAEFLQM
GVYRCFLPPQSPGIVNLYLSADGTKPISQLFSFEHRSVPVIEKVVPQEDQLYKWEEFEFQVRLSHLLFTSSSKISVFSSRISADNLLE
AKKLASRTSHLLNSWAYLMKSIQANELPFDQARDPLFELTLKNRLKEWLLEKVIENRNTKEYDSKGLGVIHLCAVLGYTWSILLF
SWANISLDFRDKHGWTALHWAAYYGREKMVAALLSAGARPNLVTDPTKEYLGGCTAADIAQQKGYEGLAAFLAEKCLVAQFR
DMKMAGNISGNLEGVKAETSTNPGHSNEEEQSLKDTLAAYRTAAEAAARIQGAFREHELKVRSKAVRFASKEEEAKNIIAAMKI
QHAFRNYETRRKIAAAARIQYRFQTWKMRREFLNMRKKAIKIQAVFRGFQVRRQYQKITWSVGVLEKAILRWRLKRRGFRGLQ
VSQPEEKEGTEAVEDFYKTSQKQAEDRLERSVVRVQAMFRSKKAQQDYRRMKLAHEEAQLEYDGMQELNQMDIES

>BrCAMTA5 XP_009107887.1

MAGVDSGRLIGSEIHGFHTLQDLDIRTMLEEAYTRWLRPNEIHALLSNHNYFTINVKPVHLPKSGTIVFFDRKMLRNFRKDGHN
WKKKKDGKTIKEAHEHLKVGNEERIHVYYAHGDDNPTFVRRCYWLLDKSQEHIVLVHYRETHEVQAAPATPGNSYSSSTTDHV
SAKPVTEDINSGVRNACNTARSNSLVARNHEISLHEINTLDWDELLVETDDVLYFTEQLQTAAMGSAQQGNHHAVYNGSTDIPSY
LGLGDPVYQNNSPCGAREFSSQHLHCVVDPNQQTRDSSATVADEQGDALLNNGYGSQESFGKWVNNFISDSPGSVDDPSLEAV
YTPGQESSAPPAVVHSQSNIPEQVFNITDVSPAWAYSTEKTKILVTGFFHDSFQHFGRSNLFCICGELRVPAEFLQMGVYRCFLPPQS
PGIVNLYLSADGTKPISQLFSFEHRSVPVIEKVVPQEDQLYKWEEFEFQVRLSHLLFTSSSKISVFSSRISADNLLEAKKLASRTSHL
LNSWAYLMKSIQANELPFDQARDPLFELTLKNRLKEWLLEKVIENRNTKEYDSKGLGVIHLCAVLGYTWSILLFSWANISLDFRD
KHGWTALHWAAYYGREKMVAALLSAGARPNLVTDPTKEYLGGCTAADIAQQKGYEGLAAFLAEKCLVAQFRDMKMAGNISG

NLEGVKAETSTNPGHSNEEEQSLKDTLAAYRTAAEAAARIQGAFREHELKVRSKAVRFASKEEEAKNIIAAMKIQHAFRNYETRR
KIAAAARIQYRFQTWKMRREFLNMRKKAIKIQAVFRGFQVRRQYQKITWSVGVLEKAILRWRLKRRGFRGLQVSQPEEKEGTE
AVEDFYKTSQKQAEDRLERSVVRVQAMFRSKKAQQDYRRMKLAHEEAQLEYDGMQELNQMDIES
>BrCAMTA6 XP_009112123.1
MAYRGSFGFAPQLDIQQLLSEAQHRWLRPAEICEILRNYQKFHIASEPPNRPPSGSLFLFDRKVLRYFRKDGHNWRKKKDGKTVK
EAHEKLKVGSIDVLHCYYAHGEDNENFQRRCYWMLEQELMHIVFVHYLEVKGNRISSSGIKENNSNSLSGSTSVNIDSTANTSST
LSPLCEDADSGNRDGWIHGNRVKESDSQRLVGVPALDASFENPLARYQNPPYNPLLTQTNPTNTGLMSVEGHLRNPLQNQVNW
QIPVQDSLPLQKWPMDSHGTDLALHENFGTFSSLIGSQNQQQPIGGGSFQAPFTSVEAAYIPKFGPEDLLYEASANQTLPLRKSLL
KKEDSLKKVDSFSRWVSNELAEMEDLQMQSSSGGIGWTSVETAAAASSLSPSLSKDQRFTMIDFWPKWTQTDTEVEVMVIGTF
LLSPQEVTSYSWACMFGEVEVPAEILVDGVLCCHAPPHEVGQVPFYITCSDRFSCSEVREFDFLPGSARKLNTVDIYGAYTNEASL
HLRFENLLARMSSAQEHNVFEDVGEKRRKISRIMLLKDEKESFLTSTVEKDLTEVEAKERLIREEFEDKLYLWLIHKVTEEGKGPN
ILDEEGQGVLHLAAALGYDWAIKPILAAGVSINFRDANGWSALHWAAYSGREDTVALLVSLGADSGALTDPSPELPLGKTASDLA
YGNGHRGISGFLAESSLTSYLEKLTVDGKEDASTDSSRAKAVQTVAERTATPMSYGDVPETLSMKDSLTAVLNATQAADRLHQVF
RMQSFQRKQLSEIGDKNEFGLSDELAVSFAAGKTKKAGGHSSGAAVHAAAVQIQKKYRGWKKRKEFLLIRQRIVKIQAHVRGH
QVRKQYRAIIWSVGLLEKIILRWRRKGSGLRGFKRDAVTKAPEPVCAAPAQEDDYDFLKEGRKQTEERLQKALTRVKSMAQYPE
ARAQYRRLLTVVEGIRENEASSSSAMNNN
NNNNNNSNTEEAANYNEEDDLIDIDSLLDDDTFMSLAFE
>BrCAMTA7 XP_009112533.1
MAEARRFSPNNELDVGHILSEARNRWLRPPEICEILQNYQKFQISSEPPTTPASGSVFLFDRKVLRYFRKDGHNWRKKRDGKTVK
EAHERLKAGSVDVLHCYYAHGHDNENFQRRSYWMLQEELSHIVFVHYLEVKGSRISTSYNRMQRTAEDSAQFSHETGEVFTSE
RDGYASGSINQYDHNNHPQATDSANVNGAHTPELEDAESAYNQQGSSIVYSHQQPPSTGFDPLYQMSLTPARTINGPGVTNGLRN
KKSIDSQTWEEILGNCGSGAEGLLPLQPNSEHEVLDQILQDSSFTMQAPYLSTKKQHLLDGALGEEGLKKVDSFSRWMSKELGD
VCVIADANESFTHSSSTAYLDGYVMSPSLSKEQLFSIIDFAPNWTYVGCEVNVLVTGKFLKTPEEAEREEWCCMFGQTEAPADVI
ADGILQCVAPMREAGRVPFYITCSNRLACSEVREFEYKVLESQAFGRETDDSTESLEARFVKLLCSKSDSPSSNGSDMSQVSEKIS
LLLFENDDQLDQMLMNEISQESMKEKLLQEALKESLHSWLLQKIAEGGKGPNVLDEGGQGILHFAAALGYNWALEPTVVAGVS
VDFRDVTGWTALHWAAFFGRELVIGSLIALGASPGTLTDPNPDFPSGSTPSDLAYANGFKGIAGYLSEYALTAHVSLLSLNESNAE
TSESTTPSPSSSSLTDSLTAVRNATQAAARIHQVFRAQSFQKKQMKEFGVSEERALSMLAPKTHKQGRAHSDDSVQAAAIRIQNK
FRGYKGRKDYLITRQRIIRIQAHVRGYQVRKNYRKIIWSVGILEKVILRWRRKGAGLRGFKSDALVDKMQDGTGREEDDDFFKQ
GRKQTEERLQKALARVKSMAQYPEARDQYRRLLNVVNDIQESKVEKALESSEEATCFDDDLIDIEALLGDDDTLMLPMSSTLW
NT
>BrCAMTA8 XP_009112534.1
MAEARRFSPNNELDVGHILSEARNRWLRPPEICEILQNYQKFQISSEPPTTPASGSVFLFDRKVLRYFRKDGHNWRKKRDGKTVK
EAHERLKAGSVDVLHCYYAHGHDNENFQRRSYWMLQEELSHIVFVHYLEVKGSRISTSYNRMQRTAEDSAQFSHETGEVFTSE
RDGYASGSINQYDHNNHPQATDSANVNGAHTPELEDAESAYNQQGSSIVYSHQQPPSTGFDPLYQMSLTPARTINGPGVTNGLRN
KKSIDSQTWEEILGNCGSGAEGLLPLQPNSEHEVLDQILQDSSFTMQAPYLSTKKQHLLDGALGEEGLKKVDSFSRWMSKELGD
VCVIADANESFTHSSSTAYLDGYVMSPSLSKEQLFSIIDFAPNWTYVGCEVNVLVTGKFLKTPEEAEREEWCCMFGQTEAPADVI
ADGILQCVAPMREAGRVPFYITCSNRLACSEVREFEYKVLESQAFGRETDDSTESLEARFVKLLCSKSDSPSSNGSDMSQVSEKIS
LLLFENDDQLDQMLMNEISQESMKEKLLQEALKESLHSWLLQKIAEGGKGPNVLDEGGQGILHFAAALGYNWALEPTVVAGVS
VDFRDVTGWTALHWAAFFGRELVIGSLIALGASPGTLTDPNPDFPSGSTPSDLAYANGFKGIAGYLSEYALTAHVSLLSLNESNAE
TSESTTPSPSSSSLTDSLTAVRNATQAAARIHQVFRAQSFQKKQMKEFGVSEERALSMLAPKTHKQGRAHSDDSVQAAAIRIQNK
FRGYKGRKDYLITRQRIIRIQAHVRGYQVRKNYRKIIWSVGILEKVILRWRRKGAGLRGFKSDALVDKMQDGTGREEDDDFFKQ
GRKQTEERLQKALARVKSMAQYPEARDQYRRLLNVVNDIQESKVEKALESSEEATCFDDDLIDIEALLGDDDTLMLPMSSTLW
NT
>BrCAMTA9 XP_009112535.1

MAEARRFSPNNELDVGHILSEARNRWLRPPEICEILQNYQKFQISSEPPTTPASGSVFLFDRKVLRYFRKDGHNWRKKRDGKTVK
EAHERLKAGSVDVLHCYYAHGHDNENFQRRSYWMLQEELSHIVFVHYLEVKGSRISTSYNRMQRTAEDSAQFSHETGEVFTSE
RDGYASGSINQYDHNNHPQATDSANVNGAHTPELEDAESAYNQQGSSIVYSHQQPPSTGFDPLYQMSLTPRTINGPGVTNGLRN
KKSIDSQTWEEILGNCGSGAEGLLPLQPNSEHEVLDQILQDSSFTMQAPYLSTKKQHLLDGALGEEGLKKVDSFSRWMSKELGD
VCVIADANESFTHSSSTAYLDGYVMSPSLSKEQLFSIIDFAPNWTYVGCEVNVLVTGKFLKTPEEAEREEWCCMFGQTEAPADVI
ADGILQCVAPMREAGRVPFYITCSNRLACSEVREFEYKVLESQAFGRETDDSTESLEARFVKLLCSKSDSPSSNGSDMSQVSEKIS
LLLFENDDQLDQMLMNEISQESMKEKLLQEALKESLHSWLLQKIAEGGKGPNVLDEGGQGILHFAAALGYNWALEPTVVAGVS
VDFRDVTGWTALHWAAFFGRELVIGSLIALGASPGTLTDPNPDFPSGSTPSDLAYANGFKGIAGYLSEYALTAHVSLLSLNESNAE
TSESTTPSPSSSSLTDSLTAVRNATQAAARIHQVFRAQSFQKKQMKEFGVSEERALSMLAPKTHKQGRAHSDDSVQAAAIRIQNK
FRGYKGRKDYLITRQRIIRIQAHVRGYQVRKNYRKIIWSVGILEKVILRWRRKGAGLRGFKSDALVDKMQDGTGREEDDDFFKQ
GRKQTEERLQKALARVKSMAQYPEARDQYRRLLNVVNDIQESKVEKALESSEEATCFDDDLIDIEALLGDDDTLMLPMSSTLW
NT
>BrCAMTA10 XP_009122514.1
MADRGSFGFISPPRLDMEQLLSEAQHRWLRPAEICEILRNYHKFHIATESPTRPASGSLFLFDRKVLRYFRKDGHNWRKKKDGKTI
KEAHEKLKVGSIDVLHCYYAHGEGNENFQRRCYWMLEVELMHIVFVHYLEVKGSRTSIGMKENNSNSLSGTASVNIDSAASPTS
RLSSYCEDADSGDSHQSSSVLRASPEPQTGNRNGWTSAPGMRTVSQVHGNRVGETDSQRLFDVQTWDAVDNLVTRYDQPCNN
LLLEERTDKGGMLPAEHLRSPLQTQLNWQIPAQDDLPLPKWPGYLLPHSGMTDDTDLALLEQSAQDNFESFSSLLDIEHLQSDGI
SPSDMESEYIPVKKSLLRHEDSLKKVDSFSRWASKELGEMEDLQMQSSRGDIAWTSVDCETAAAGVAFSPSLSEDQRFTILDYWP
KCAQTDADVEVVVIGTFLLSPQEVTICSWSCMFGEVEVPAEILVDGVLCCHAPPHTAGQVPFYVTCSNRFACSELREFDFRSGST
KKIDAAGIYGYSTKEASLQMRFEKLLAHRDFVQEHQIFEDVVEKRRKISKIILLNEEKENLFPRIYERHSTKQEPKELVLRQQFED
ELYIWLIHKVTEEGKGPNILDEGGQGVLHFVAALGYDWAIKPILAAGVNINFRDANGWSALHWAAFSGREETVAVLVSLGADAG
ALTDPSPELPLGKTAADLAYGKEHRGISGFLAESSLTSYLEKLTMESKENSPANSGGPKAVQTVSERTAAPMSSGDIPETLSLKDSL
TAVRNATQAADRLHQVFRMQSFQRKQLSGFDVDDDDEIGISNELAVSFAASKTKNPGQSEIFVHSAATHIQKKYRGWKKRKEFL
LIRQRVVKIQAHVRGHQVRKQYKPIVWSVGLLEKIILRWRRKGTGLRGFKRNAVPKTVEPEPQCPMVPKEDDYDFLEKGRKQTE
ARLEKALTRVKSMVQYPEARDQYRRLLTVVEGFRENEASSSLSVNNREEPVNYEDDDLIDIDSLLNDDILMSTSPXMTPFVYSFS
FFRLSNFLHLFSVNIIS
>BrCAMTA11 XP_009127544.1
MQSEYEISNLYHEAQTRWLKPPEVHFILQNHERYQLTHKAPQNPPSGSLLLFNRRVLKFFRKDGHQWRRKKDGRAIAEAHERLK
VGNVEALSCYYVHGEHDPTFQRRIYWMLDPEYDHIVLVHYRDISDGKEGRQTSGTVLQFSQNASTLFSSPSSIGTQNASYNHYIG
DSADVLQQHSSTSPEVNSEVVFNSNGVETPQGSGSSYEFENRQAIKRLEEQLSLGDDIVSTVDPLYAQNESLDSLQFLAQPGTVY
QRPENNKLERCYGGYVGAQYNVDPLYSQNESLDSLLSLDSAEDINHLAQQATGHQRPENNRLERSYGGYIGADYHPNNVTLAK
NDTGGNGGSGDQESESWKDVLEACEASVALNSEGSTPSSVKGLLPGMQEDSNWSYSNQADQSALLLPQELGSFEHPACYPELG
DPENNAEYSRIMDDEGIIRMPLQQEMRPTVSHKQEFTIQDVSPQWGYANETTKVIIIGSFLCDPTESTWSCMFGSVEVPFEIIKDGV
IRCEAPPCGPGKVNLRITSGDGLSCSQIKEFEYRDKPDTSCSRCSRDELFLLVRFVQTLLSDKKSNIEPDIDKLKKIKADDDEEWSH
IIDTILDDTATPSSTVDWLLQKLLKDKLDAWLSSRSQDEDQTSCSLSKQEQGIIHMVAGLGFEWALHPILGLGVSVDFRDSNGWS
ALHWAARYGREKMVAALIASGASAGAVTDPNAQDPAGKTAASIGASNGHKGLAGYLSEVALTNHLSSLTLEETEHSLGSAQVQA
EMIVNSISGRSPPGNDDPHSRAALRNVAQAAARIQAAFRAHSFRKRQEREAAMAACYQEYGIYADIEGIAAMSKLAFGNVKNYN
SAALSIQKKYRGYKGRKEFLAKRQKVVKIQAYVRGYQVRKHYKVICWAVGILDKVVLRWRRKGVGLKGFRQDVESREEESED
EDILKVFRKEKVDGAVNEAFSRVLSMTNSPDARQQYQRVLKRYCQTKAELGKTETLGTDGGDEDDDVLLDIADMRYENLRTLP
>BrCAMTA12 XP_009140343.1
MAEARRFGLNNELDVGQILSEARNRWLRPPEICEILQNYQKFQISTEPPTTPASGSVFLFDRKVLRYFRKDGHNWRKKRDGKTV
KEAHERLKAGSVDVLHCYYAHGQDNENFQRRSYWMLQEELSHIVFVHYLEVKGSRVSTSYNRMQRTEDSTRSSQETGEVYTSE
RNGYASGSINQYDHSNNQSQATDSASVNGVHTPELEDAQSAYNQQGSPILYSHQALQQPPATSFDPYYQMSLTPRDSYQKEIHTIS
SSTMVEKGRTINGPVVTNSIKNKKSIDSQTWEEILGNCGSGGEGLPMQPHSEHEGLDQMLQSYSFTMQDFASLQESIVKSQNQEL

NSGLTSDRSLWLQGQAVDIEPNALSNLASSEKAPYLSTMKQHLLDGALGEEGLKKMDSFNRWMSKELGELGDVGVTADANESF
THSSSTAYWEEVESEDVSNGGYVMSPSLSKEQLFSIIDFAPNWTYVGCEVKVLVSGKFLKMAESGEWCCMFGQTEVPADIIANGI
LECVAPMHEAGRVPFYVTCSNRLACSEVREFEYKVLESQGFDRETYDSSTGCNSIESLEARFVKLLCSKSDCTNSSLPGGNDSDL
SQVSEKISLLLFENDDQLDQMLMNEISQENMKNNLLQEALKESLHSWLLQKIAEGGKGPNVLDEGGQGVLHFAAALGYNWALE
PTIVAGVSVDFRDVNGWTALHWAAFFGRELIIGSLIALGASPGTLTDPNPDFPSGSTPSDLAYANGYKGIAGYLSEYALRTHVSLLS
LNEKNAETSLGGAVEAAPSPSSSALTDSLTAVRNASQAAARIHQVFRAQSFQKKQMKEFGDRKLGMSEERALSMLAPKTHKQGR
GHSDDSVQAAAIRIQNKFRGYKGRKDYLITRQRIIKIQAHVRGYQVRKNYRKIIWSVGILEKVILRWRRKGAGLRGFKSDALVTK
MQDGTEKEEDDDFFKQGRKQTEERLEKALARVKSMVQYPEARDQYRRLLNVVNDIQESKVEKALANSEEATCFDDDLIDIEAL
LGDDDTLMMPMSSTLWNA

>BrCAMTA13 XP_009146049.1

MDGDGSSGRLIGSEIHGFHTLQDLDVQTMLEEARSRWLRPNEIHAVLSNPKYFTINVKPVNLPTSGRIILFDRKMLRNFRKDGHN
WKKKKDGRTVKEAHEHLKVGDEERIHVYYAHGEDNTTFVRRCYWLLDKARENIVLVHYRDTQEAATTSGDSNSHSSSNLVAVE
DIDFNPDDSLYLGINNTPVVKTHETRLHDINTLDWDELLVQSDLDNQSAPIVDDMSYFTEHLQNATKDTAEHLTVADESLDALLN
NGPQSRENFGRWMNSFIGDPNGSLEDPSFEAMVTLDQNPLAPQATFHPHSDLPQQVFNITEVSPAWAYSSEKTKIVVTGILHNSYQ
HLGSSNLFCICGDSCVPAEYVQAGVYRCFIPPHSPGMANLYLSADGHEPISQCFTFEHRPAPVSAKPVHENDQESKWEEFELQVR
LAHLLFTSSNKLNVLSSNISPENFLDAKKLSNKTSHLLNSWAYLIKSVQGSKVSFDQAKDQIFELTLKNRIKEWLMEKVLEGRNA
RDYDSKGLGVIHLCAILGYTWSIQLFSLSGLSLDFRDKQGWTALHWAAYYGREKMVAALLTAGAKPNLVTDSRKDNLGGCTPA
DLAQQNGFDGIAAYLAEKCLVAQFVAMKLAGNISGTLETCKGEMSNQGPLPDDEQNLKDALAAYRTAAEAAARIQGAFREKAL
KAARSKVIQFENKEEEAKSIIAAMKIQNAFRKYDTRRKIEAAYRIQCRFQTWKMRREFLNMRRQAIRIQAAFRGLQARKQYRKIL
WSVGVLEKAIIRWRQKRKLFIGLQVSEAEAEEKAEEDFYKASQKQAEERLERSVVRVQAMFRSKKAQEDYRRMKLTHEETQLE
YEFLHDV

>SlCAMTA1 Solyc01g057270.2.1

MYLLMESKVDLEQILKELHHRWLLPHEVCQILRNHQSFCLTQQLQLKPPAGSIFLYDRKLLPNFCKDGHHWRKNKDGQTIKEAH
EKFKAGSVDVLHCYYVHGEGNKNFQRRSYWMLEEQLEHIVLVHYRDVKEVFPQQFNDMVVAGYRLGASRLQPVHPGLLLENP
DSSSKPCFVFGPAFQKSHTSNPSLVDLKEQALSSELHSGDSKGLVAFSRSKERFQLNPQVRAFMSSGFRKFERNLNVMLQRKFYS
GHYNLADLRSSKLTYAKLYAGKAVANNRSRLAITSGKVFEENIHVAPPQIQNISSSQTVVTPDAAVKTSSLDGGLNSDEVGSLKKL
DILGKWMDREFAGGNKSLMSSDSGNYWNTLDTDNGDKEVSTLSRHLLLEANSVGTSPSQKQLFRIFDFSPQWAFSGVETKVLIV
GTFLVHRKYLTCLKWSCMFGEVEVSAEVQTQSIRCQVPFHAPGHVPFYVTCGNRLACSEVREFEYREKSSELALALRPSDEVHL
QVQLVKLLYSGLNKKFLDCSSRECENCKLKTQLCSLKCQTGNATERLEDLLAVIECDHINFKDVQIQNFMKDKLYEWLVSRAHE
EDKGPNILNDQGKGVIHLVAALGYEWGLLPLIAAGISPNFRDACGRTALHWAAHYGREDMVIALIKLGVAAGAVDDPTTASPGG
RTAADLASSRGYKGIAGYLAESDLTSHHQLLATSKNALDTIGAGLEAEKVYESAVQEIVPLNGTIDDDVSLKASLASLRKSAHAA
ALIQAAFRARSFRQRQLRESRNDVSEASLDLVALGSLNKVQKVNCFEDYLHSAAINIQQKYCGWKGRREFLKVHNQIVKMQAL
VRGHEVRKQYKKFVWAVSILEKGILRWRRKKTGLRGFWPEKTSETGIVEREKEEEYDYLSIGLKQKCAGVEKALGRVESMVRH
PEARDQYMRMVAKFKSCKLDDGGREVNRSSPPV

>SlCAMTA2 Solyc01g105230.2.1

MEDCGSDPPGFRLDITQILSEVQHRWLRPAEICEILRNHRKFHLTPEAPFRPVSGSVFLFDRKVLRYFRKDGHNWRKKKDGKTVK
EAHEKLKVGSIDVLHCYYAHGEEDDNFQRRSYWMLEQDLMHIVFVHYLEVKGNKVNVSSIRSTKSVHPNYLNDCSLSDSFSTR
HKKLTSANADSTSLASTLTEAHEEAESEDSHQACSRFHSYPDRASGMDSHLVENRDTISSSYGSPQSSVEYTPLPGIDGSGKCDLG
NFASGPQRTIDLGSWEPLPQHCLNGEMVCQDDFKNNLSVHGNWQFHGQNVNQDLIADSSYDLGLPSDLLTVRGPSYLYSNEKE
EQLAQLNLQFLKSLVEVQGDINQENSMDMLELGDYSTIKQPHLSSVKVEEGLKKVDSFSRWVAKELEDVEELHMQPSNQMSWN
VIDTEEEGSCLPSQLHVDSDSLNLSLSQEQVFSIIDFSPNWAYSNLETKVLITGRFLKSEGELVEYKWSCMFGEVEVPAEVLADGV
LRCHAPPHKPGVLPFYVTCSNRLACSEVREFEYRFGPYQEVGAADVSMTEKHLLERIENLLSLGPVSSCRSSDSMEDSEEKRSTV
NKIISMMEEENQPIIERASYGDTSQCRVKEDLYFERKLKQNFYAWLVHQVTDDGRGRTLLDGEGQGVLHLVAALGYDWAFKPIL
ASGVSVDFRDMNGWTALHWAAFYGREKTVVSLVSLGASPGALTDPSAEFPLGRTPADLASANGHKGISGFVAESSLTTHLSKLTV

TDAKEELDSEVCEAKVGETVTERVAVSTTENDVPDVLSLKDSLAAIRNATQAAARIHQIFRVQSFQRKQIIEHCDNELSSDENAIAI
VASRACKLGQNNGIAHAAAIQIQKKFRGWNKRKEFLLIRQKIVKIQAHIRGHQVRKKYKPIIWSVGILEKVILRWRRKRSGLRGF
RSEAVMSKPSTQEDSLPEDDYDFLKEGRKQTEVRMQKALARVKSMTQYPEGRAQYRRLLTAAEGLREVKQDGPIQIPEIPEDTIY
PEEELFDVDSLLDDDTFMSIAFE

>SlCAMTA3 Solyc04g056270.2.1

MADSRRYGLNAQLDIEQILLEAQHRWLRPAEICEILKNYQKFRIAPEPPNRPPSGSLFLFDRKVLRYFRKDGHSWRKKRDGKTVK
EAHERLKAGSIDVLHCYYAHGEENENFQRRSYWMLEEEMSHIVLVHYREVKGNRTNFSRIREPQQVTPDLQETDEDVHSSEVDS
SASAKFYPNDYQVNSQVTDTTSFSSAQASEYEDAESVYNQHPTSGFHSFLDAQPSAGDGLAVPYHPIPFSNDQVQFAGSSGTSFS
SIPPGNGNTSTANTYVPSRNLDFASWGTISVNNPAAYQSLHFQPSGQSSANNMMHEQGNTTMGQICSNDFTRQEHENHIDGLGN
WQTSEVDSSFISKWSMDQKLNPDLTSGQTIGSSGVYGVEHHNSLEASQLLPAQQDKHPIQNELQSQLSDANIGGSLNADLDHNL
SLGVKTDYSALKQPLLDGVLKREGLKKLDSFDRWISKELGDVSESHMQSNSSSYWDNVGDEDGVGNSTIASQVQLDTYVLSPS
LAQDQIFSIIDFSPNWAFSGSEIKVLITGRFLKSQQEVENCSWACMFGELEVPAEVIADGVLRCHTPVQKAGRVPFYITCSNRLACS
EVREFEFRVTEEKVKDQLLQKLLKEKLHVWLLQKVAEGGKGPNILDEGGQGVLHFAAALGYDWAVPPTIAAGVSVNFRDVNG
WTALHWAASYGRERTVGFLISLGAATGALTDPTPKHPSGRTPADLASSNGHKGIAGYLAESSLSSHLFSLELKEKKQGENEQAFG
EAVQTVSERTATPAWDGDWSHGVSLKDSLAAVRNATQAAARIHQVFRVQSFQRKQLKEYGGSEFGLSDERALSLLAMKTNRAG
QHDEPHAAAVRIQNKFRSWKGRRDFLLIRQRIIKIQAHVRGHQVRNKYKNIIWSVGILEKVILRWRRKGSGLRGFKPEAPTEGSN
MQDQPVQEDDYDFLKEGRKQTEERLQKALERVKSMVQYPEARDQYRRLLNVVSDMQEPNSTAASYNSAEAVDFNDDLIDLGD
LLDDDTFMPTAP

>SlCAMTA4 Solyc05g015650.2.1

MAESGYDINDLVREAQIRWLKPAEVLFILRNHENHQLSSEPSQKPPSGSLFLYNKRVLRFFRKDGHSWRKKKDGRTVGEAHERL
KVGNAEALNCYYAHGEQNPNFQRRSYWMLDPAYDHIVLVHYRDIIEGRQNPAFMSESSPISSAFSPSPSSYSTPHTGSTGIASECY
EQYQNQSSPGEICSDAIINNNGTTDTIGRTEEVISSPGLEMCQALRRLEEQLSLNDDSLKEIDPLYGDAINDDSSLIQMQGNSNRLL
LQHHSGESSESHHRDLTQDAHVWKDMLDHYGVSAAAESQTKYLHKLDENAMLQTLSERRAIEAYESYKWRDFSDKETQTAPV
QAFKQLEDFKYPTYPPDITTFGSNPDEYTTIFDQDQIGTSLEDEMSLTIAQKQKFTIRHISPDWGYSSEPTKIVIIGSFLCNPSECTWT
CMFGDIEVPIQIIQEGVICCQAPRHLPGKVTLCVTSGNRESCSEVREFEYRVKPDDCARNNQPDVEGAYRSTDELLLLVRFVQLLL
SDLSVQKRESSELGNDLLEKSKASEDSWSQIIESLLFGTSVPMVTIDWLLQELLKDKFQQWLCSKLQQKDNQIDCSLSKKEQGIIH
MVAGLGFEWALHPILNAGVSANFRDINGWTALHWAARFGREKMVASLIASGASAGAVTDPSSRDPVGKTAASIVALTSHLSSLTL
EESELSKGTADVEAERTISSISNTSATINEDQRSLKDTLAAVRNAAQAAARIQSAFRAHSFRKRQQREFGVSATTSVDEYGILSNDI
QGLSAASKLAFRNPREYNSAALAIQKKYRGWKGRKDFLAFRQKVVKIQAHVRGYQVRKQYKVCWAVGILEKVVLRWRRRGV
GLRGFRHDTESIDEIEDEDILKVFRKQKVDAALDEAVSRVLSMVESPGARQQYHRILEKYRQSKAELEGADSETASTAHGHV

>SlCAMTA5 Solyc12g035520.1.1

MAESGYNTNDLVQEGRFRWLRPAEVLFILQNHDDRQLAHQPPQKPASGSMFLFNKRVLRYFRKDGHSWRKKKDGRTVGEAHE
RLKVGNAEALNCYYAHGEKNSNFQRRSYWILDPAYEHIVLVHYRDITEGRQIAAFMSQSSPISSTFPLSPSLYSTQHPGFNVPGTES
YQQYQDESRPGYGEICSDAVIHSNGMNVSDITRMMEGVSNSPKVEISQALRRLEEQLNLNDDSSSDIYSLYSEIENSNDAENVVH
DKSSLVQIQDNSNNFLFLPHSGESSESRDQLLNLDNSMWKEMLDHCRSSPASQPQAKCFEKLDENGMLQTSSGSEPIEAIKSDRW
PIIGGKEALKCSVTNLKQVDDFKYIGCAQINVFGSYPDQCTTIFDQDQIGISSETNMSLTIVQKQKFTIHDISPDWGYASDATKVVII
GSYLCNPSEYTWTCMFGDTEVPVQIIKDGAIRCQAPPHLPGKVALCVTTGNRTPCSEVREFEYRAKFDDRGQNVVPEVGGASKS
SEELLLLVRFVQMLLSDSSVQIGDGSELSNDILEKSKASEDSWSQVIESLLFGTSTSTVTIDWLLQELLKNKLQQWLSSKLQVKNN
EMVYSLSRKDQGIVHMIAGLGFEWALHPVLNAGVSANFRDIRGWTALHWAARFGREKMVASLIASGAFAGAVTDPSSQDPFGK
TAASIASSCGHKGVAGYLSEVALTSHLTSLTLEECDVSKGTADIEAEQTISNITTTSPVTHEDQLSLKDTLDAVRNAAQAAARIQSA
FRAHSFRKRRLREAAHVATTCRDEYCILSNDVLGLSAASKLAFRNVRDYNSAALSIQRKYRGWKGRKDFLVFRQKVVKIQAHV
RGYQVRKEYKVCWAVGILEKVVLRWRRRGVGLRGFRLEDEPIEESENEDILKLFRKQKVDAAINEAVSRVLSMVDSPEARQQYR
RILEKYRQAKAEVAGAKSDAISTAHSDISNVENNDVYHS

>SlCAMTA6 Solyc12g099340.1.1

MESSVSGRLLGCEIHGFRTMQDLDIPNIMEESKMRWLRPNEIHAILCNHKYFNINVKPVNLPKSNLILSGTIVLFDRKMLRNFRRD
GYNWKKKKDGKTVKEAHEHLKVGNDERIHVYYAHGEDNTTFVRRCYWLLDKTLEHVVLVHYRETQEVSSNSTVAQGSPAAP
VSSGSALSDPADLSASWVLSGELDSAVDQQYSASRHAHLEPNRDMTVQNHEQRLLEINTLEWDDLLAPGDPNKMVATQQAVGK
TAYVQHTSYEQRNLCELNGYSFDGGVSSSLERISTFNNSNEITFQTVDGQMTSSFEKNESGVMTVSTGDSLDSLNQDRLQTQDSF
GRWMNYLIKDSPESIDDPTPESSVSTGQSYAREQIFNITEILPAWAPSTEETKICVIGQFHGEQSHLESSSLRCVCGDACFPAEVLQP
GVYRCIVSPQTPGLVNIYLSFDGNKPISQVMSFEFRAPSVHVWTEPPENKSDWDEFRNQMRLAHLLFSTSKSLNILSSKIHQDLLK
DAKKFAGKCSHIIDDWACLIKSIEDKKVSVPHAKDCLFELSLKTRLQEWLLERVVEGCKISEHDEQGQGVIHLCAILGYTWAVYP
FSWSGLSLDYRDKYGWTALHWAAYYGREKMVATLLSAGAKPNLVTDPTSENLGGCTASDLASKNGHEGLGAYLAEKALVAQF
KDMTLAGNISGSLQTTTESINPGNFTEEELNLKDSLTAYRTAADAAARIQAAFRERALKVRTKAVESSNPEMEARNIIAAMKIQHA
FRNYEMQKQLAAAARIQYRFRTWKMRKEFLHMRRQAIKIQAVFRGFQVRRQYRKIIWSVGVLEKALFRWRLKRKGLRGLKLQ
STQVTKPDDVEEDFFQASRKQAEERIERSVVRVQAMFRSKQAQEQYRRMKLEHDKATLEYEGTLNPDTEMD

>OsCAMTA1 LOC_Os07g30774

MAGAGGWDPLVGSEIHGFLTYPDLNYEKLVAEAAARWFRPNEIYAILANHARFKIHAQPVDKPVSGTVVLYDRKVVRNFRKDG
HNWKKKKDGRTVQEAHEKLKIGNEERVHVYYARGEDDPNFFRRCYWLLDKDLERIVLVHYRQTAEENAMAPPNPEPEVADVP
TVNLIHYTSPLTSADSTSGHTELSLPEEINSHGGISASSETGNHDSSLEEFWANLLESSIKNDPKVVTSACGGSFVSSQQINNGPKNS
GNIVNTSMASNAIPALNVVSETYATNHGLNQVNANHFGALKHQGDQTQSLLASDVDSQSDQFISSSVKSPMDGNTSIPNEVPAR
QNSLGLWKYLDDDSPGLGDNPSSVPQSFCPVTNERLLEINEISPEWAYSTETTKVVVIGNFYEQYKHLAGSAMFGVFGEQCVAG
DIVQTGVYRFMVGPHTPGKVDFYLTLDGKTPISEICSFTYHVMHGSSLEARLPPSEDDYKRTNLKMQMRLARLLFATNKKKIAPK
LLVEGTKVANLMSALPEKEWMDLWNILSDPEGTYVPVTESLLELVLRNRLQEWLVEMVMEGHKSTGRDDLGQGAIHLCSFLGY
TWAIRLFSLSGFSLDFRDSSGWTALHWAAYHGRERMVATLLSAGANPSLVTDPTPESPAGLTAADLAARQGYDGLAAYLAEKGLT
AHFEAMSLSKDTEQSPSKTRLTKLQSEKFEHLSEQELCLKESLAAYRNAADAASNIQAALRERTLKLQTKAIQLANPEIEASEIVA
AMKIQHAFRNYNRKKAMRAAARIQSHFRTWKMRRNFINMRRQVIRIQAAYRGHQVRRQYRKVIWSVGIVEKAILRWRKKRKG
LRGIASGMPVVMTVDAEAEPASTAEEDFFQAGRQQAEDRFNRSVVRVQALFRSYKAQQEYRRMKIAHEEAKIEFSEGQLGAAC
RS*

>OsCAMTA2 LOC_Os04g31900

MSLSFDINVLHKEARSRWLKPSEVYYILQNHERFPITPEPPKKPPSGSLFLYNRRVNRYFRRDGHAWRRKKDGRTVGEAHERLKV
GNVDALSCYYAHGEQNPCFQRRCFWMLEPAYEHIVLVQYREVGAAEGRYNSASLLNGPTDSLSVLSYPNATYGNQYLGSTSGV
SDGSESLHSNLSSVTEVSSYSANKDNGILQSIQELSQSTIMGAPALGQSSLEQSIEVRWVDNSNSTNKSGLNRALKQIVEQLSLGD
DEDDDYIHQAQPFDFITNIEAPDRQRDASRNVSGGSQAKQIRAEEMQNGLGRGIPSSWEDVLQSSSGFPAPSIYQSTPHYPQNSEY
QPPGSLYNSDMQQISAAKRFLLETEDSIDSPSYNYVPREEGNNGTNTLSVHDYSLQSSLNPDWKKTAPLTLQSNLYGSEIPSLLLD
HGQFESLSSGENTRLILGQNPRFSIREVSPEWTYCYEITKVIITGDFLCDPSSSCWAVMFGDSEVPAEIVQAGVLRCHTPLHSSGKL
TICVTSGNREICSEVKDFEFRAKSTASSFLDISPSSRSLKSSEELLLLAKFVRMLLCENGSHANSNGDPQSVQCPKLKMNDEHWQR
LIDELKGGCENPLNVSDWIMEELLKSKLQQWLSVKLQGYDGIACSLSKHEQGIIHLISALGYEWALSSILSADVGINFRDTNGWT
ALHWAAYFGREKMVAALLAAGASAPAVTDPTAQDPVGKTAAFLASERGHLGLAAYLSEVSLTSYLASLTIQESDTSKGSAAAEA
ERAVESISQRNAQLHGGTEDELSLKDSLAAVRNAAQAAARIQNAFRAFSFRKRQQKTARLKDEYGMTQEDIDELAAASRSYYQS
LLPNGQFYDKAAVSIQKKFKGWKGRRHFLNMRRNAVKIQAHVRGHQVRKKYKTFVSTVSVLEKVILRWRRKGHGLRGFRAEQ
TAMAEAEEDDEDDDDDDFNDDEAVKVFRRQKVDESVKEAMSRVLSMVDSPEARMQYRRMLEEFRQATAE*

>OsCAMTA3 LOC_Os10g22950

MAAAADARRFAVVPQLDIAQILKEAQQRWLRPAEICEILKNYKSFRIAPEPPNRPQSGSLFLFDRKVLRYFRKDGHNWRKKKDG
KTVKEAHERLKSGSIDVLHCYYAHGEENENFQRRTYWMLEEDFMHIVLVHYLETKGGKSRTRGNNDMHQAAVMDSPLSQLPS
QTIDGESSLSGQFSEYEEAESDVYSGGTGYHSFTQMQQQQNGIGPVTDASMFSSRVSASSIGNYQGQHAMGHTTNFYSSSQHDS
PLVLSDPNLELANNGHESLWNGVMKPDEGTVQMTHLQPPVHPEQGMFTTEGQGVEYLTFDEVYSDGLSLKDIGAAGADVEPF
WQLSSATADISATENSVQQNDGSLGAAIGFPFLKTQSSNLSDILKDSFKKSDSFTRWMSKELLDVEDSQIQSSSGAYWNTEEADSII
EASSREPLDQFTVAPMVLQDQLFSIVDFSPSWTYAGSKTKVLVTGRFLHANEVTERCKWSCMFGEVEIQAEISADGTLRCYSPPH

KPGRVPFYVTCSNRLACSEVREFEFRPSDSQYMDAPSPLGATNKVYFQIRLDNLLSLGPDVYQATITNPSKEMIDLSKKISSLLAN
NDEWSKLLKLADDNEPLSHDQQDQYAENLIKEKLHVWLLHKVGDGGKGPSVLDDEGLGVLHLAAALGYDWAIRPTVTAGVNI
NFRDFHGWTALHWAAFCGRERTVVALIALGAAPGALTDPHPNYPAESTPADLASANGHKGISGFLAESSLTSHLQALNLKEANM
SEISGLPGIGDVTERNASQPAIGDSLGAVRNAAQAAARIYQVFRVQSFQRKQAVQYEGDKGGISDEHALSLLSMKPSKSGQLDPL
HAAASRIQNKYRGWKGRKEFLLFRQRIVKIQAHVRGHQVRKHYRKIVWSVGIVEKVILRWRRRRAGLRGFRPTEGAIESSSGGT
SSNLVKDKPAGDDYDFLQEGRKQTEERLQKALARVKSMVQYPEARDQYQRILNVVSKMQESQTVQEKILDESTEMDEGDFMSE
FKELWDDDTPLPGYF*

>OsCAMTA4 LOC_Os07g43030

MAEVRKYGLPNQPPDIPQILLEAQNRWLRPTEICHILSNYKKFSIAPEPPNRPASGSLFLFDRKILRYFRKDGHNWRKKKDGKTVK
EAHEKLKVGSVDVLHCYYAHGEENENFQRRTYWLLEEGFMNIVLVHYLEVKGKNNFSRVASKVSAVLKKLKRVQDYLMLIVLH
AQTLSLVRASVSKEKFGATDNCRASSRYHPFVEMQQPVDGVMMNNMLGVSAPSAGYHGEMQTTTANSDNHFATHYDIAGVFN
EAGAGLRGVSKTLHDSVRFAEPYPECSAEFMEPALYSSNATMESNNLDDNSRLETFMSEALYTNNLTQKEADALSAAGIMSSQA
ENNSYTDGIRYPLLKQSSLDLFKIEPDGLKKFDSFSRWMSSELPEVADLDIKSSSDAFWSSTETVNVADGTSIPINEQLDAFAVSPSL
SQDQLFSIIDVSPSYACTGSRNKVLITGTFLANKEHVENCKWSCMFGDVEVPAEVLAHGSLRCYTPVHLSGRVPFYVTCSNRVAC
SEVREFEFRDSDARQMDTSDPQTTGINEMHLHIRLEKLLSLGPDDYEKYVMSDGKEKSEIINTISSLMLDDKCLNQAVPLDEKEV
STARDQNIEKLVKEKLYCWLVHKVHDEDKGPNVLGKEGQGVIHLVAALGYDWAVRPIITAGVKVNFRDARGWTALHWAASCG
RERTVGALIANGAESGLLTDPTPQFPAGRTAADLASENGHKGIAGFLAESALTSHLSALTLKESKDGNVKEICGLGGAEDFAESSS
AQLAYRDSQAESLKDSLSAVRKSTQAAARIFQAFRVESFHRKKVVEYGDDDCGLSDERTLSLVSIKNAKPGQNDGSHSAAVRIQN
KFRGWKGRKEFMIIRQKIVKIQAHVRGHQVRKSYRRIVWSVGIVEKIILRWRRKRRGLRGFQPVKQLEGPSPIQQLEGPSQIQPAK
EEEEDEYDYLKDGRKQAEGRLQRALARVKSMTQYPEAREQYSRIANRVTELQEPQAMMIQDDMQSDGAIADGGDFMAELEEL
CGDGDAPMPTIL*

>OsCAMTA5 LOC_Os03g09100

MAEGRRYAIAPQLDIEQILKEAQRRWLRPTEICEILKNYRSFRIAPEPPNRPPSGSLFLFDRKVLRYFRKDGHNWRKKRDGKTVKE
AHERLKSGSIDVLHCYYAHGEENINFQRRSYWMLEEDYMHIVLVHYLEVKAGKLSSRSTGHDDVLQASHADSPLSQLPSQTTEG
ESSVSGQASEYDETESDIYSGGARYNSFSRMRQHENGGGSVIDDSIFSSYVPASSVGSYQGLQATAPNTGFYSHGQDNLPVVLNE
SDLGTAFNGPNSQFDLSLWIEAMKPDKGTHQIPLYQAPVPSEQSPFTGGPGIESFTFDEVYNNGLSIKDVDGDDTDGETPWQIPNA
SGTFATADSFQQNDKTLEEAINYPLLKTQSSSLSDIIKDSFKKNDSFTRWMSKELAEVDDSQITSSSGVYWNSEEADNIIEASSSDQ
YTLGPVLAQDQLFTIVDFSPTWTYAGSKTRVFIKGNFLSSDEVKRLKWSCMFGEFEVPAEIIADDTLVCHSPSHKPGRVPFYVTCS
NRLACSEVREFDFRPQYMDAPSPLGSTNKIYLQKRLDKLLSVEQDEIQTTLSNPTKEIIDLSKKISSLMMNNDDWSELLKLADDN
EPATDDKQDQFLQNRIKEKLHIWLLHKVGDGGKGPSMLDEEGQGVLHLAAALGYDWAIRPTIAAGVNINFRDAHGWTALHWA
AFCGRERTVVALIALGAAPGAVTDPTPSFPSGSTPADLASANGHKGISGFLAESSLTSHLQTLNLKEAMRSSAGEISGLPGIVNVAD
RSASPLAVEGHQTGSMGDSLGAVRNAAQAAARIYQVFRMQSFQRKQAVQYEDENGAISDERAMSLLSAKPSKPAQLDPLHAAA
TRIQNKFRGWKGRKEFLLIRQRIVKIQAHVRGHQVRKHYRKIIWSVGIVEKVILRWRRRGAGLRGFRPTENAVTESTSSSSGNVT
QNRPAENDYDFLQEGRKQTEERLQKALARVKSMVQYPDARDQYQRILTVVTKMQESQAMQEKMLEESTEMDEGLLMSEFKEL
WDDDMPTPGYF*

>OsCAMTA6 LOC_Os03g27080

MFGDVEVPAEVLADGSLRCYAPEHQSGRVPFYVTCSNRIACSEVREFEYRDSDAQYMETSHSQANGINEMHLQIRLEKLLTLGP
DDNQLLVCGNEKLELINAINSLMLDEKWSDQGSPSGSKDVVTPRNQSLKKLMKEKLHCWLIYKIYDCEKGPNILGKEGQGIIHL
AAALGFDWAIRPILVAGVNVNFRDAHGWTALHWAASCGRERTVGVLIANGAAAGALTDPTSEFPSGRTPADLASTNGHKGIAGF
LAESALTSHLSALTLKESKDSNAEEACRLTIPEDLPEMNYGQLAVQDSHAESLKDSLSAVRKSAQAAARIFQAFRVESFHRKKVV
EYGDDDCGLSDEHTFSLISLQKVKQGQHDTRLHSAAVRIQNKFRGWKGRKEFMIIRQRIVKLQAHVRGHQVRKNYKKVVWSV
GIVEKVILRWRRKGRGLRGFRPEKQLEGQTQIQPAKTEDEYDYLQDGRRQAEGRLQRALDRVRSMTQYPEAREQYRRLTTCVA
EMQQSRMMQDEMLSEAAGADGSDFMNGLEDLICRDDPQMSAIW*

>OsCAMTA7 LOC_Os01g69910

MQHQQGFDTHRLHQEVKSRWLKPKEVLQILQNHDRFIITHKTPHKPPSGAWFLFNRRVLRYFRNDGYEWRKKKNGKTIAEAHE
RLKVDNVDALNCYYAHADKNSTFQRRIYWMLDPAYDHIVFVHYRDVQEGSISVSALNDSSTSNQNGSGSRAEAQSSPGLTSELF
APCLNSCSPGSAEEVSSQIMAINNETNSVSQPDWVQHCNQAALRKLKVQLSLEDREDHDVDAKDIPSNSEPITVYGIQNEEPGTC
RNLADVFSGLEFSKENHPEETGLPFSSTIDVLKNSDTWLEEDQIEAILHSASMIVTENQWFNIREVSPEWSYCSESTKVIIAGDFLR
DPSHGSWAIVFGDVKVHAEIVQQGVIRCHTPCLDARKVTMYLIDENEKACSEARQFEFHNKPTKSVVCENRKPCREVHESELHQ
RPTESNNELLLLFNYAQLLFDGHVSEQFLKFGLPFPNLECGLQVSPSEIMKGASERLNRDTAVNCVMEVLLNNKFEEWLFSKYEQ
NSEGNHFLPRQYHGVIHTIAALGYNWALKLLLNSGVLVNYRDANGWTALHWAARFGREETVVLLLDAGAAAGALSDPTAQDP
AAKTPASVASAYGFKGLSAYLSEAELIAHLHSLESKENGSSGDQISRVVGRISDTSAHAQSGSDDQLALKESLGAMRYAVQAAGR
IQTAFRIFSFRKKQQAGLQNRGNHIISIREVGAASHGMLEKAALSIQKNFRCWKKRKEFLKIRKNVIKIQARVRAHQQHNKYKEL
LRSVGILEKVMLRWYRKGVGLRGFHPGAIAMPIDEEDEDDVAKVFRKQRVETALNKAVSRVSSIIDSPVARQQYRRMLKMHKQ
NKDDDEKVEVSPASHVYGSGSHHMCWLSHNNKAMH*

>NtCAMTA2 XP_016435125.1
QLLLFYFSGYDINDLVREAQIRWLKPAEVLFILRNHENHQLSNEAAQKPPSGSLFLFNKRVLRFFRKDGHSWRKKKDGRTVGEA
HERLKVGNAEALNCYYAHGEQNPTFQRRSYWMLDPAYEHIVLVHYRDITEGRQNPAFMSESSPISSTFSPSPSSYSTQQTGSTLIA
GESYEQYQNQSSPGEICSDAVINNNGMSDIIGRTKEVMSSPGLEMSQALRRLEEQLSLNDDSFKEIDPLYADAISDDSSLVEMQGN
SNSLLLQHHSAESSESHHQHLTQDGHVWKDMLDHYGVSTAAESLTKSLPKLDENGMLQISSERGAIEAYQSYKWPNFSEKEAQK
APIPAFKQLENFKYPAYSPGVTAFGSNSDQCTTIFDQDQIGTSFEDEMSLTISQKQKFTFRDISPDWGYSSEATKVVIIGSFLCNPSEC
MWTCMFGDSEVPVQIIQEGVICCQAPPHLPGKVTLCVTSGNRESCSEVKEFEYRDKPDDCARNNRSDVEGAYKSTEELLLLVRF
VQLLLLDLSAQKEDSSMLSNDFLEKCKANEDSWSQVIESLLFGTSTSTITIDWLLQELLKDKFQQWLSYKLQRKDNQMGCSLSK
KEQGIIHMVSGLGFEWALHPILNAAVSVNFRDINGWTALHWAARFGREKMVASLIASGASAGAVTDPSSRDPVGKTAASIASSCG
HKGLAGYLSEVALTSHLSSLTLEESELSKGTADVEAEKTISSISNTSATTNEDQRSLKDSLAAVRNAAQAAARIQSAFRAHSFRKR
QQRESAVTATASGDEYGILSNNIHGLSAASKWAFRNTRDYNSAALAIQKKYRGWKGRKDFLAFRQKVVKIQAHVRGYQVRKQ
YKVCWAVGILEKVVLRWRRRGVGLRGFRHDTESIDESEDEDILKVFRKQKVDAALDEAVSRVLSMVESPGARQQYHRILEKYR
QAKAELEGAESESASTAHGDMSNMENDDIYQFSSY
>NtCAMTA8 XP_016452770.1
MADSRRYGLNAQLDIDQILLEAQHRWLRPAEICEILKNYQKFRIAPEPPNRPPSGSLFLFDRKVLRYFRKDGHSWRKKKDGKTVK
EAHERLKAGSIDVLHCYYAHGEENENFQRRSYWMLEEEMSHIVLVHYREVKGNRTNFSRTREPQEAAPRFQETDEDVHSSEVDS
SASTKFYPNDYQVNSQVTDTTSLSSVQASEYEDAESAYNQHPTSGFHSFLDAQPSMTQKAGEGLAVPYHPIPFSTDDHQVQFAGS
SDMDFFSIAPGNKSGNTANTYIPSRNLDFPSWETTSVNNPAAYQSYHFQPSSQSGANNMTHEQGNTKTGQVFLNDFKRQERQNR
IDGLGDWQTSEGDAAFISKWSMDQKLHPDLASDHTIRSSAAYNVELHNSLEASHILPSHQDKHPMQNELPSQLSDPNVGGSLNA
DLDHNLSIGVRTDHSSLKQPLLDGVLREGLKKLDSFDRWMSKELEDVSEPHMQSNSSSYWDNVGDDDGVDNSTIASQVQLDTY
MLSPSLSQDQFFSIIDFSPSWAFAGSEIKVLITGKFLKSQPEVEKCSWACMFGELEVPAEVIADGVLRCHTPIQKAGRVPFYITCCNR
LACSEVREFEFRVTEGQDADVANANSCSSSESLLHMRFGKLLSLESTVSLSSPPRSEDDVSHVCSKINSLLNEDDNEWEEMLNLT
YENNFMAEKVKDQLLQKLLKEKLRVWLLQKVAEGGKGPNVLDEGGQGVLHFAAALGYDWAIPPTIAAGVSVNFRDVNGWTA
LHWAASYGRERTVGFLISLGAAPGALTDPTPKHPSGRTPADLASSNGHKGIAGYLAESSLSFHLSSLELKEMKQGENVQPFGEAV
QTVSERSATPAWDGDWPHGVSLKDSLAAVRNATQAAARIHQVFRVQSFQRKQLKEHGGSEFGLSDEHALSLLALKTNKAGQHD
EPVHTAAVRIQNKFRSWKGRRDYLLIRQRIIKIQAHVRGHQVRNKYKNIIWSVGILEKVILRWRRKGSGLRGFKPEATLTEGSDTQ
DRPVQEDDYDFLKEGRKQTEQRLQKALARVKSMVQYPEARDQYRRLLNVVSDMKDTTTTSDGAPSNSVEAADFGDDLIDLDD
LLDDDTFMSTAP
>NtCAMTA11 XP_016452774.1
MADSRRYGLNAQLDIDQILLEAQHRWLRPAEICEILKNYQKFRIAPEPPNRPPSGSLFLFDRKVLRYFRKDGHSWRKKKDGKTVK
EAHERLKAGSIDVLHCYYAHGEENENFQRRSYWMLEEEMSHIVLVHYREVKGNRTNFSRTREPQEAAPRFQETDEDVHSSEVDS
SASTKFYPNDYQVNSQVTDTTSLSSVQASEYEDAESAYNQHPTSGFHSFLDAQPSMTQKAGEGLAVPYHPIPFSNDHQVQFAGSS

DMDFFSIAPGNKSGNTANTYIPSRNLDFPSWETTSVNNPAAYQSYHFQPSSQSGANNMTHEQGNTKTGQVFLNDFKRQERQNRI
DGLGDWQTSEGDAAFISKWSMDQKLHPDLASDHTIRSSAAYNVELHNSLEASHILPSHQDKHPMQNELPSQLSDPNVGGSLNA
DLDHNLSIGVRTDHSSLKQPLLDGVLREGLKKLDSFDRWMSKELEDVSEPHMQSNSSSYWDNVGDDDGVDNSTIASQVQLDTY
MLSPSLSQDQFFSIIDFSPSWAFAGSEIKVLITGKFLKSQPEVEKCSWACMFGELEVPAEVIADGVLRCHTPIQKAGRVPFYITCCNR
LACSEVREFEFRVTEGQDADVANANSCSSSESLLHMRFGKLLSLESTVSLSSPPRSEDDVSHVCSKINSLLNEDDNEWEEMLNLT
YENNFMAEKVKDQLLQKLLKEKLRVWLLQKVAEGGKGPNVLDEGGQGVLHFAAALGYDWAIPPTIAAGVSVNFRDVNGWTA
LHWAASYGRERTVGFLISLGAAPGALTDPTPKHPSGRTPADLASSNGHKGIAGYLAESSLSFHLSSLELKEMKQGENVQPFGEAV
QTVSERSATPAWDGDWPHGVSLKDSLAAVRNATQAAARIHQVFRVQSFQRKQLKEHGGSEFGLSDEHALSLLALKTNKAGQHD
EPVHTAAVRIQNKFRSWKGRRDYLLIRQRIIKIQAHVRGHQVRNKYKNIIWSVGILEKVILRWRRKGSGLRGFKPEATLTEGSDTQ
DRPVQEDDYDFLKEGRKQTEQRLQKALARVKSMVQYPEARDQYRRLLNVVSDMKDTTTTSDGAPSNSVEAADFGDDLIDLDD
LLDDDTFMSTAP

>NtCAMTA13 XP_016452780.1

MADSRRYGLNAQLDIDQILLEAQHRWLRPAEICEILKNYQKFRIAPEPPNRPPSGSLFLFDRKVLRYFRKDGHSWRKKKDGKTVK
EAHERLKAGSIDVLHCYYAHGEENENFQRRSYWMLEEEMSHIVLVHYREVKGNRTNFSRTREPQEAAPRFQETDEDVHSSEVDS
SASTKFYPNDYQVNSQVTDTTSLSSVQASEYEDAESAYNQHPTSGFHSFLDAQPSMTQKAGEGLAVPYHPIPFSRNKSGNTANTY
IPSRNLDFPSWETTSVNNPAAYQSYHFQPSSQSGANNMTHEQGNTKTGQVFLNDFKRQERQNRIDGLGDWQTSEGDAAFISKWS
MDQKLHPDLASDHTIRSSAAYNVELHNSLEASHILPSHQDKHPMQNELPSQLSDPNVGGSLNADLDHNLSIGVRTDHSSLKQPLL
DGVLREGLKKLDSFDRWMSKELEDVSEPHMQSNSSSYWDNVGDDDGVDNSTIASQVQLDTYMLSPSLSQDQFFSIIDFSPSWAF
AGSEIKVLITGKFLKSQPEVEKCSWACMFGELEVPAEVIADGVLRCHTPIQKAGRVPFYITCCNRLACSEVREFEFRVTEGQDADV
ANANSCSSSESLLHMRFGKLLSLESTVSLSSPPRSEDDVSHVCSKINSLLNEDDNEWEEMLNLTYENNFMAEKVKDQLLQKLLK
EKLRVWLLQKVAEGGKGPNVLDEGGQGVLHFAAALGYDWAIPPTIAAGVSVNFRDVNGWTALHWAASYGRERTVGFLISLGA
APGALTDPTPKHPSGRTPADLASSNGHKGIAGYLAESSLSFHLSSLELKEMKQGENVQPFGEAVQTVSERSATPAWDGDWPHGVS
LKDSLAAVRNATQAAARIHQVFRVQSFQRKQLKEHGGSEFGLSDEHALSLLALKTNKAGQHDEPVHTAAVRIQNKFRSWKGRR
DYLLIRQRIIKIQAHVRGHQVRNKYKNIIWSVGILEKVILRWRRKGSGLRGFKPEATLTEGSDTQDRPVQEDDYDFLKEGRKQTE
QRLQKALARVKSMVQYPEARDQYRRLLNVVSDMKDTTTTSDGAPSNSVEAADFGDDLIDLDDLLDDDTFMSTAP

>NtCAMTA14 XP_016464002.1

MADCGSDPSGFRLDITQILSEVQHRWLRPAEICEILRNYKKFHITPEAPHRPVSGSVFLFDRKVLRYFRKDGHNWRKKKDGKTVK
EAHEKLKVGSIDVLHCYYAHGEEDDNFQRRSYWMLEQDLMHIVFVHYLEVKGNKANMGCVRSIKSAHSNYLNDCSLSDSFPR
GHKKLASANADSTSVASTLTSAHEEAESEDSHQACSRFQSYPERASGMDRNLVENRDTIYSSYGSPQSSVEYTSLPGIDVGEKCG
LGNFASGPQRTIDLGSQEPVSQHCSNGEIVCQDDFKNNLSVKGNWQIIYLSSPCPCFPSFYVPXDLVNSFHNKNLSSDLYTGRGQS
YLYPDEQEEQLTQLNIQYLNSLVEVQGDFNQENSMDMLGLGDYYTIKQPHLNSVKMEEGLKKVDSFSRWVVKELEDVEELHM
QPTNRISWNVIDTLDDGSCLPTQLHVDSDSLNPSLSQEQVFSIIDFSPNWAYSNLETKVLITGRFLKSEGELIECKWSCMFGEIEVP
AEVLADGVLRCHAPPHKPGVLPFYVTCSNRLACSEVREFEYRLGAYQEFGAANVSATEMHLLERIESLLSLEPLSSCHSSDSMEA
AKEKQSTVNRIICMMEEENQQMIERASDHDTSQCGVKEDLFLERKLKQNFYAWLVRQVTDDGRGRTAIDDEGQGVLHLAAALG
YDWALKPILASGVSVDFRDMNGWTALHWAAFYGREKTVVGLVSLGASPGALTDPSAEFPLGRTPADLASANGHKGISGFLAESS
LTTHLSKLTVTDAKEELASEVSGAKVGETVTERVAVTTTGDDMPDVLSLKDSLAAIRNATQAAARIHQIFRVQSFQRKQIIECSDN
ELSSDENALSIVASRACKLGQNNGIAHAAATQIQKKFRGWNKRKEFLLIRQKIVKIQAHVRGHQVRKKYKPIIWSVGILEKVILR
WRRKRSGLRGFRSEVVMNKPIIQDDSLPEDDYDFLKEGRKHTEVRMQKALARVKSMTQYPEGRAQYRRLLTAAEGLREVKPD
GPTCILESPEDTSYPEEELFDVENLLDDDTFMSIAFE

>NtCAMTA15 XP_016472343.1

MKFEVFIFAPFLLVTDTGRYLYNKSLDVGQILQEAQHRWFRPDEICEILRNHQKFGLTPQPPLRPPGGSLYLFDRKVLPYFCEDGH
QWRKEEDGKAVKEIHEKLKAGSNDVLHCYYAHGEDNENFQRRSYWMLEEPMEHIILVHYREVKEGYIVGASCLQPVHPGLLLE
NPQSSSAPCFESDLIVQESHTSTPSSVDWKEYVLSSELHSGHAKRNEADPLLVPAGLVESSRDSFQLNSRFSSLLSGEVLEENTHIA
PPQIQNISISQTVVSPDTAVQISSLEGGVNSDEAGSLKKFDSYVRWMDREKSRDCDESLMATKSGNYWNTLDTDNEGKSCNLSIQ

MEYERKRTDTQLPLAPFGHSSFQYPFLSKDIITEILIRLPVKLIVKFRCVSKSWRALITSPKFVKDHLRLALKNKAYHKVMWCHRP
QIKIEECYLSSLFDNSTTFSDVSALHPRLVSDGLYTRVPDSIVVLGSVHGLICLIGSVDGCIFQGYDDIDTERLVDIEDFKEDLFLWN
PSTRRYKKLPDHKPDYWFDTRVYGLGYDMLHDDYKFLGTFRGYQRLHCCKVQLYSLKSDSWKSLDDLPSALSGGNLQGVSGT
FVEGSLHWLSYTTDGPDKRWNIISFNLADEKWGTLEKPCYEEGELVLWLGVIGSDLSLFVDCEGTHIDVWVMQKYAVQGSWNK
KFIIKYPDGVGLTGFGPLFMLPYFFSITDEILMLFNSTANIYNTRDEVWRPNPYVINWLDHPTAFIYVESLVSPFSAKGTEATRKNL
LDVAADPTLPHTISVKCPQSDHKK

>NtCAMTA5 XP_016478076.1

MADSRRYGLNAQLDIDQILLEAQHRWLRPAEICEILKNYQKFRIAPEPPNRPPSGSLFLFDRKVLRYFRKDGHSWRKKKDGKTVK
EAHERLKAGSIDVLHCYYAHGEENENFQRRSYWMLEEEMSHIVLVHYREVKGNRTNFSRTREPQEATPRFQETDEDVHSSEVDS
SASTKFYPNGYQVNSQVTDATSLSSAQASEYEDAESAYNQHPTSGFHSFLDAQPSMMQKAGESLPVPYHPIPFSTDDHQVQFAG
SSDMDFFSSAPGNKSRNTANTYIPSRNLDFPSWETISVNNPAAYQSYHFQPSSQSGANNMTHEQGSTTMGQVFLNDFKKQGQNR
IDSLGDWQTSEGDAAFISKWSMDQKLNPNLASDHTIRSSAAYNVELHNSLEASHILPSHQDKHPMQNELPSQLSDANVGGSLNA
ELDHNLSIGVRTDHSSLKQPLLDGVLREGLKKLDSFDRWMSKELEDVSEPHMQSNSSSYWDNVGDDDGVDNSTIASQVQLDTY
MLSPSLSQDQFFSIIDFSPSWAFAGSEIKVLITGKFLKSQPEVEKWACMFGELEVPAEVIADGVLRCHTPNQKVGRVPFYITCSNRL
ACSEVREFEFRVSESQDVDVANSCSSSESLLHMRFGKLLSLESTVSLSSPPRSEDDVSNVCSKINSLLKEDDNEWEEMLNLTYENN
FMAEKVKDQLLQKLLKEKLRVWLLQKVAEGGKGPNVLDEGGQGVLHFAAALGYDWAIPPTIAAGVSVNFRDVNGWTALHWA
ASYGRERTVGFLIISLGAAPGALTDPTPKHPSGRTPADLASSNGHKGIAGYLAESSLSSHLSSLELKEMKQGETVQPFGEAVQTVS
ERSATPAWDGDWPHGVSLKDSLAAVRNATQAAARIHQVFRVQSFQRKQLKEHGGSEFGLSDEHALSLLALKTNKAGQHDEPVH
TAAVRIQNKFRSWKGRRDYLLIRQRIIKIQAHVRGHQVRNKYKNIIWSVGILEKVILRWRRKGSGLRGFKPEATLTEGSNMQDRP
VQEDDYDFLKEGRKQTEQRLQKALARVKSMVQYPEARDQYRRLLNVVSDMKDTTTTSDGAPSNSGEAADFGDDLIDLDDLLD
DDTFMSTAP

>NtCAMTA6 XP_016478077.1

MADSRRYGLNAQLDIDQILLEAQHRWLRPAEICEILKNYQKFRIAPEPPNRPPSGSLFLFDRKVLRYFRKDGHSWRKKKDGKTVK
EAHERLKAGSIDVLHCYYAHGEENENFQRRSYWMLEEEMSHIVLVHYREVKGNRTNFSRTREPQEATPRFQETDEDVHSSEVDS
SASTKFYPNGYQVNSQVTDATSLSSAQASEYEDAESAYNQHPTSGFHSFLDAQPSMMQKAGESLPVPYHPIPFSNDHQVQFAGSS
DMDFFSSAPGNKSRNTANTYIPSRNLDFPSWETISVNNPAAYQSYHFQPSSQSGANNMTHEQGSTTMGQVFLNDFKKQGQNRID
SLGDWQTSEGDAAFISKWSMDQKLNPNLASDHTIRSSAAYNVELHNSLEASHILPSHQDKHPMQNELPSQLSDANVGGSLNAEL
DHNLSIGVRTDHSSLKQPLLDGVLREGLKKLDSFDRWMSKELEDVSEPHMQSNSSSYWDNVGDDDGVDNSTIASQVQLDTYM
LSPSLSQDQFFSIIDFSPSWAFAGSEIKVLITGKFLKSQPEVEKWACMFGELEVPAEVIADGVLRCHTPNQKVGRVPFYITCSNRLA
CSEVREFEFRVSESQDVDVANSCSSSESLLHMRFGKLLSLESTVSLSSPPRSEDDVSNVCSKINSLLKEDDNEWEEMLNLTYENNF
MAEKVKDQLLQKLLKEKLRVWLLQKVAEGGKGPNVLDEGGQGVLHFAAALGYDWAIPPTIAAGVSVNFRDVNGWTALHWAA
SYGRERTVGFLIISLGAAPGALTDPTPKHPSGRTPADLASSNGHKGIAGYLAESSLSSHLSSLELKEMKQGETVQPFGEAVQTVSE
RSATPAWDGDWPHGVSLKDSLAAVRNATQAAARIHQVFRVQSFQRKQLKEHGGSEFGLSDEHALSLLALKTNKAGQHDEPVHT
AAVRIQNKFRSWKGRRDYLLIRQRIIKIQAHVRGHQVRNKYKNIIWSVGILEKVILRWRRKGSGLRGFKPEATLTEGSNMQDRPV
QEDDYDFLKEGRKQTEQRLQKALARVKSMVQYPEARDQYRRLLNVVSDMKDTTTTSDGAPSNSGEAADFGDDLIDLDDLLDD
DTFMSTAP

>NtCAMTA9 XP_016485864.1

MADCGSDPPGFRLDITQILSEVQHRWLRPAEICEILRNYRKFHITPEAPHRPVSGSVFLFDRKVLRYFRKDGHNWRKKKDGKTVK
EAHEKLKVGSIDVLHCYYAHGEEDDNFQRRSYWMLEQDLMHIVFVHYLEVKGNKANVGCVRSIKSAHSNYLNDCSLSDSFPRS
LKKLASVNADSTSVASTLTSAHEEAESEDSHQACSRFQSYPERASGMDRHLVENRDAIYSSYGSPQSSVEYTSLSSIDGGGKCGR
GNFASGPQRTIDLGSQEPVSQHCSNGEMVCQDDFKNNLSVQRNWQYSFGDSASQFHGQIVNQDLIADSSYDLVNSFHNKNLSSD
LYTGRGQSYLYPDEQEEQLTQLNIQYLNSLVEVQGDFNQENSMDMLGLGDYSTIKHPHLNSVKMEEGLKKVDSFSRWVVKELE
DVEELHMQRTNRISWNVIDTEDDGSCLPTQLHVDSDSLNPSLSQEQVFSIIDFSPNWAYSNLETKVLITGRFLKSEGELIECKWSC
MFGEVEVPAEVLADGVLRCHAPPHKPGVLPFYVTCSNRLACSEVREFEYRLGAYQEIGAANVSATEMHLLERIESLMSLGPVSSC

HSSDSMEAAKEKHSTVNKIICMMEEENQQMIERASDYDTSQCGVKEDLFLERKLKQNFYAWLVRQVTDDGRGRTAIDDEGQGV
LHLAAALGYDWALKPILASGVSVDFRDMNGWTALHWAAFYGREKTVVGLVSLGASPGALTDPSAEFPLGRTPADLASANGHKG
ISGFLAESSLTTHLSKLTVTDATEELASEVSGAKVGETVTERVAVTTTGDDVPDVLSLKDSLAAIRNATQAAARIHQIFRVQSFQRK
QIIERSDNELSSDENALSIVASRACKLGQNNGIAHAAATQIQKKFRGWNKRKEFLLIRQKIVKIQAHVRGHQVRKKYKPIIWSVGI
LEKVILRWRRKRSGLRGFRSEVVINKPSIQDDSLPEDDYDFLKEGRKQTEVRMQKALARVKSMTQYPEGRAQYRRLLTAAEGLR
EVKQDGSTCIQESSEDTSYPEEELFDVENLLDDDTFMSIAFE

>NtCAMTA10 XP_016485865.1

MADCGSDPPGFRLDITQILSEVQHRWLRPAEICEILRNYRKFHITPEAPHRPVSGSVFLFDRKVLRYFRKDGHNWRKKKDGKTVK
EAHEKLKVGSIDVLHCYYAHGEEDDNFQRRSYWMLEQDLMHIVFVHYLEVKGNKANVGCVRSIKSAHSNYLNDCSLSDSFPRS
LKKLASVNADSTSVASTLTSAHEEAESEDSHQACSRFQSYPERASGMDRHLVENRDAIYSSYGSPQSSVEYTSLSSIDGGGKCGR
GNFASGPQRTIDLGSQEPVSQHCSNGEMVCQDDFKNNLSVQRNWQYSFGDSASQFHGQIVNQDLIADSSYDLVNSFHNKNLSSD
LYTGRGQSYLYPDEQEEQLTQLNIQYLNSLVEVQGDFNQENSMDMLGLGDYSTIKHPHLNSVKMEEGLKKVDSFSRWVVKELE
DVEELHMQRTNRISWNVIDTEDDGSCLPTQLHVDSDSLNPSLSQEQVFSIIDFSPNWAYSNLETKVLITGRFLKSEGELIECKWSC
MFGEVEVPAEVLADGVLRCHAPPHKPGVLPFYVTCSNRLACSEVREFEYRLGAYQEIGAANVSATEMHLLERIESLMSLGPVSSC
HSSDSMEAAKEKHSTVNKIICMMEEENQQMIERASDYDTSQCGVKEDLFLERKLKQNFYAWLVRQVTDDGRGRTAIDDEGQGV
LHLAAALGYDWALKPILASGVSVDFRDMNGWTALHWAAFYGREKTVVGLVSLGASPGALTDPSAEFPLGRTPADLASANGHKG
ISGFLAESSLTTHLSKLTVTDATEELASEVSGAKVGETVTERVAVTTTGDDVPDVLSLKDSLAAIRNATQAAARIHQIFRVQSFQRK
QIIERSDNELSSDENALSIVASRACKLGQNNGIAHAAATQIQKKFRGWNKRKEFLLIRQKIVKIQAHVRGHQVRKKYKPIIWSVGI
LEKVILRWRRKRSGLRGFRSEVVINKPSIQDDSLPEDDYDFLKEGRKQTEVRMQKALARVKSMTQYPEGRAQYRRLLTAAEGLR
EVKDGSTCIQESSEDTSYPEEELFDVENLLDDDTFMSIAFE

>NtCAMTA3 XP_016489138.1

MAESGYDINDLVREAQIRWLKPAEVLFILRNHEYHQLSNEPAQKPPSGSLFLFNKRVLRFFRKDGHSWRKKKDGRTVGEAHERL
KVGNAEALNCYYAHGEQNPNFQRRSYWMLDPVYEHIVLVHYRDITEGRQNPAFMSESSPISSTFSPSPSSYSTQQTGSAVIAGESY
EQYQNQFSPGEICSDAVINNNRTSDITGRTNEVMSSPGLEMSQALRRLEEQLSLNDDSFKEIDPLYADAISDDSSLVEMQGSSNSLL
LQHHSAESSESHHQHLTQDGHIWKDMLDHYGVSTADESLNKSLPKLDENGMLQISSERGAIEAYQSYKWPNFSEKEAQKAPIPA
FKQLENFKYPAYSPGVTAFGSNSDQCTTIFDQDQIGTSLEDEMSLTISQKQKFTIRDISPDWGYSSEATKVVIIGSFLCNPSECMWT
CMFGDTEVPIQIIQEGVICCQAPPHLPGKVTLCVTSGNRESCSEVKEFEYRVKPDDCARNNRSDIEGAYKSTEELLLLVRFVQMLL
LDLSVHKEDSSELSNDFLEKSKANEDSWSQVIESLLFGTSTSTITIDWLLQELLKDKFQQWLSYKLQRKDNQMGCSLSKKEQGII
HMVSGLGFEWALHPILNAGVSVDFRDINGWTALHWAARFGREQMVASLIASGASAGAVTDPSPRDPVGKTAASIASSCGHKGLA
GYLSEVALTSHLSSLTLEESELSKGTADVEAEKTISSISNTSATTNEDQRSLKDSLAAVRNAAQAAARIQSAFRAHSFRKRQQRESA
IATTASGDEYGILSNDILGLSAASKWAFRNTRDYNSAALAIQKKYRGWKGRKDFLAFRQKVVKIQAHVRGYQVRKQYKVCWAV
GILEKVVLRWRRRGVGLRGFRH

>NtCAMTA12 XP_016491317.1

MTELYNKQPTIAPVSSPNTKTVKYSRFQVGSGNRILNERGRERAILHHREILLLRISMAESGYNINNLVREGRFRWLRPAEVLFILQ
NHEDQQLANQPPQKPASGSMFLFNKRVLRYFRKDGHSWRKKKDGRTVGEAHERLKVGNAETLNCYYAHGEKNPNFQRRSYW
MLDPAYEHIVLVHYRDITEGMQIAAFMSQSSPISSTFSLSPSLYSTQHPGFTVVGSESYQQYQNESSPGSGEICSGAGINSNGMNIS
DITGRTEGVSSSPQVEISQALRKLEEQLSLNETDPLYSEIENSDDVENFGHDNSSLVQIQHKSNNLLLQPYSGESSESQHQLLNLDG
DIWKEMLDHCRSFPAAESQDKCFEKLDENGTLQTLSGMGPIEVTESDRWLKFGGKEALKSSLTNFKQVEDFKYPACARINTYGS
YSDQYTTIFDQDLIGTSFEDDMSLTIAQKQKFTFHDISPDWGYSSEATKVMIVGSFLCNPSEYTWTCMFGDIEVPVQIIKEGAIRCQ
APPHLPAEVGGACKSSEELLHLVRFVQMLLSDSSVQKGDGSGSSNDILENSKASEDSWSQVIESLLFGTSTSMVTVDWLLQELLK
DKLQQWLSSKLQVQNNQMGYSFSRKEQGIIHMVAVLGFEWALQPILDAGVSVNFRDINGWTALHWAARFGREKMVASLVASGA
FAGAVTDPSSQDPFGKTAASIASSCGHKGVAGYLSEVALTSHLSSLTLEESELSKGAADVEAERTISSISTTNAATHEDQLSLKDTL
AAVRNAAQAAARIQSAFRAHSFRKRRQREAARAATTSGDEYCVLSNDVLGLSAASKLAFRNMRDYNSAALAIQKKYRGWKCR
KGFLAFRQKVVKIQVLYLFESRIDISTSFVTTYKIKDDRSKKKRCHQFRCY

>NtCAMTA1 XP_016491500.1

MESSRAGQLAGSDIHGFHTLQDLDIPSIMEEAKMRWLRPNEIHAILCNYKYFNIFVKPVNLPMSGTIVLFDRKMLRNFRKDGHN
WKKKKDGKTVKEAHEHLKVGNEERIHVYYAHGEDHPTFVRRCYWLLDKSLEHIVLVHYRETQEAQGSPATSVAKGSPATPVNS
NSSSDPSDPSGWVLSEKCNSVDERTYGSSQHAHLEPNRDVTAKNHEQRLLEINTLEWDELLAPDNPNKLIATQEAGGRASVGQQ
NQIEVNGYSLNDGSLSVSRVPVASLESFVCQVAGSDTVNFNPSNDMPFHSGDGQMTSNFRKNEPGVTTVGAGDSFDSLNKDGLQ
TQDSFGRWINYFISDSPGSADEMMTPESSVTIDQSYVMQQIFNITEISPTWALSSEETKILVIGHFPGAQSQLAKSNLFCVCADVCFP
AEFVQSGVYRCVISPQPPGLVSLYLSFDGNTPISQVMTYEFRAPSACKWTAPLEEQSSWDEFRVQMRLAHLLFSTSKSLSIFSSKV
HQDSLKEAKRFVRKCSHITDNWAYLIKSIEDRKLPVPHAKDCLFELSLQTKFHEWLLERVIGGCKTSEWDEQGQGVIHLCAILGY
TWAVYPFSWSGLSLDYRDKYGWTALHWAAHYGREKMVATLLSAGAKPNLVTDPTSENPGGSTAADLASKNGFEGLGAYLAEK
ALVAHFKDMTLAGNVSGSLQTTTEHINPGNFTEEELYLKDTLAAYRTAADAAARIQAAFREHSFKVQTKAVESSNPEMEARNIVA
AMKIQHAFRNYESRKKLAAAARIQYRFRSWKMRKDFLNMRRHAIKIQAVFRGFQVRKQYRKIVWSVGVLEKAVLRWRLKRKG
FRGLQVQSSQAVDIKPDGDVEEDFFRASRKQAEERVERSVVRVQAMFRSKRAQEEYRRMKLEHDNATLEYERASLLNPDIQIG

>NtCAMTA4 XP_016507792.1

MQVGNEERIHVYYAHGEDHPTFVRRCYWLLDKSLEHIVLVHYRETQEAQGSPATSVAKGSPATPVNSNSSSDPSDPSGWVLSEK
CNSVDERTYGSSQHAHLEPNRDVTAKNHEQRLLEINTLEWDELLAPDNPNKLIATQEAGGRASVGQQNQIEVNGYSLNDGSLSV
SRVPVASLESFVCQVAGSDTVNFNPSNDMPFHSGDGQMTSNFRKNEPGVTTVGAGDSFDSLNKDGLQTQDSFGRWINYFISDSP
GSADEMMTPESSVTIDQSYVMQQIFNITEISPTWALSSEETKILVIGHFPGAQSQLAKSNLFCVCADVCFPAEFVQSGVYRCVISPQ
PPGLVSLYLSFDGNTPISQVMTYEFRAPSACKWTAPLEEQSSWDEFRVQMRLAHLLFSTSKSLSIFSSKVHQDSLKEAKRFVRKCS
HITDNWAYLIKSIEDRKLPVPHAKDCLFELSLQTKFHEWLLERVIGGCKTSEWDEQGQGVIHLCAILGYTWAVYPFSWSGLSLDY
RDKYGWTALHWAAHYGREKMVATLLSAGAKPNLVTDPTSENPGGSTAADLASKNGFEGLGAYLAEKALVAHFKDMTLAGNVS
GSLQTTTEHINPGNFTEEELYLKDTLAAYRTAADAAARIQAAFREHSFKVQTKAVESSNPEMEARNIVAAMKIQHAFRNYESRKK
LAAAARIQYRFRSWKMRKDFLNMRRHAIKIQAVFRGFQVRKQYRKIVWSVGVLEKAVLRWRLKRKGFRGLQVQSSQAVDIKP
DGDVEEDFFRASRKQAEERVERSVVRVQAMFRSKRAQEEYRRMKLEHDNATLEYERASLLNPDIQIG

>NtCAMTA7 XP_016507793.1

MESSRAGQLAGSDIHGFRTLQDLDIPSIMEEAKMRWLRPNEIHAILCNYKYFNIFVKPVNLPTSGTIVLFDRKMLRNFRKDGHNW
KKKKDGKTVKEAHEHLKVGNEERIHVYYAHGEDHPTFVRRCYWLLDKSLEHIVLVHYRETQETQGSPVTSVAKGSPATPVNSNS
SSDPSDPSGWVLSEECNSVDERAYGSSQHAHLEPNRDMTAKNHEQRLLEINTLEWDELLAPENPNKLNATQEAGGRASAGQQN
QFEVNGYSLNDGSLSVSRVPVASLESFVCQVAGSDTVNFNPSNDTSFRSGDGQMTSNFQKNESGVTTVGAGDSFDSLNKDGLQT
QDSFGRWINYFISDSPGSADEMMTPESSVTIDQSYVMQQIFNITEISPTWALSSEETKILVIGHFPGGQSQLAKSNLFCVCADVCFP
AEFVQSGVYRCVISPQPPGLVNLYLSFDGNTPISQVMTYEFRAPSARKWTAPLEEQSSWDEFRVQMRLAHLLFSTSKSLSIFSSKV
HQDSLKEAKRFVRKCSHITDNWAYLIKSIEDRKLPVPHAKDCLFELSLQTKFHEWLLERVIGGCKTSEWDEQGQGVIHLCAILGY
TWAVYPFSWSGLSLDYRDKYGWTALHWAAHYGREKMVATLLSASAKPNLVTDPTSENPGGSTAADLASKNGFEGLGAYLAEK
ALVAHFKDMTLAGNVSGSLQTTTEHINSGNFTEEELYLKDTLAAYRTAADAAARIQAAFREHSFKVQTKAVESSNPEIEARNIVA
AMKIQHAFRNYESRKKLAAAARIQYRFRSWKMRKDFLNMRRHAIKIQAVFRGFQVRKQYRKIVWSVGVLEKAVLRWRLKRKG
FRGLQVQSSQAVDIKPDGDVEEDFFRASRKQAEERVERSVVRVQAMFRSKRAQEEYRRMKLEHDNATLEYERASVLNPDIQIG

>NtCAMTA16 XP_016506959.1

MADTRRYLSNQPLDLEQVLQETQHRWLRPAEICEILRNHHKFYLTPEPPVRPPGGSLFLFDRKVLRYFRKDGHQWRKKKDGKTV
KEAHEKLKAGSVDVLHCYYAHGENNENFQRRSYWMLEEKLEHIVLVHYREVIESYRVGASRLQPIHPGQLLENPSSSPCFVSGLI
VQESHTSSPSSVDWKEQALSSELYTGDSKGNEVNPLLVPASGHFLPITSSFSTEKPTGLVEFSRDNFQLNPQFGSFVSIDAQSSDRN
LNVTLQKKFYSGYLNVADLLSSKLTYARLDGGRAVKDVANSRNRLTITSGEVLEENIHLAPAQIQNISSSQTVVTPDAAVQNSSLE
GRLNSDEAGSLKKLDSFGRWMDREIAVDGNESLLASDSGNYWNTLDNGDKEVARLSCHMQLDTNSLGPFLSQEQLFSISDFAPD
WAYSGVETKVLIIGTFLGHGKHPTSQKWSCMFGEVEVSAELLTQSIIRCEVPSHSPGRVPFYVTCSNRLACSEVREFEYREKSSEL
ALALRPSDEVRLQVRLAKLLYSGLNKKFLDCSSTDCERGKLKTLLCSLKCNIGNASESLEDLLAIIEGNHINFRDTLIQSFMKDKF
YEWLVSRAHEEDKGPNILDDEGQGVIHLVASLGYEWGLVLLTAAGINPNLRDARGRTALHWAAHYGREDMVIALVKLGVAVCA

VDDPTAAFPGGQTAADLASSGGHKGVAGYLAESELTAHLQSLAINNNALDSICAGLEAEKAFESAAQEVVPLNGTIHDDISLKGS
LASVRKSAHAAALIQAAFRARSFHQRQLRESRNDVSEASVDLVALGSLNKVQKVNHFEDYLHPAAIKIQQKYRGWKGRREFLKI
RNRIVKIQAHVRGHQVRKQYKKFVWSVSIVEKAILRWRRKKPGLRGFQPEKTSQKELPEFEKNDEYEYLSIGRKQKFAGVQKAL
ARVQSMVRHPEARDQYMRLVAKFDSFKLDDGGSSI

>NtCAMTA17 XP_016507791.1

MAESGYNINDLVREGHFRWLRPAEVVFILQNHEDQQLANQPPQKPASGSMFLFNKRVLRYFRKDGHSWRKKKDGRTVGEAHER
LKVGNAEALNCYYAHGEKNPNFQRRSYWMLDPAYEHIVLVHYRDITEGMQIAAFMSQSSPISSTFSLSPSLYSTQHPGFTVFGSES
YQQYPNESSPGSGEVCSDAGINGKGMNISDITGRTEGVSSSPRVEISQALRKLEEQLSLNDDSLEQIDPLYSEIENSDDVENFVHDN
NSLVQIQHKSNNLLLQPHSGESSESQHQLLNLDGNIWKEMLDHCRSFPAAESPAKCFEKLDENGTLQTSSGVGPIEATESDRWLK
FGGKALKSSLTNFKQVEDFKYPACARINTYGSYSDQYTTIFDQDQIGTSFEDDMSLTIAQKQKFTIHDISPDWGYSSEATKIVIVGS
FLCNPSEYTWTCMFDDIEVPVQIINEGAIRCQAPPHLPCKVTLCVTTGNRVSCSEVWEFEYRVKFDDHGQKNLAEVGGACKSSE
ELLLLVRFVQMLLSDSSVQKGDGSGSSNDILENSKASEDSWSQVIESLLFGTSTSMVTVDWLLQELLKDRLKQWLSSKLQVKNN
QMGYSFSRKEQGIIHMVAGLGFEWALHPILDAGVGVNFRDINGWTALHWAARFGREKMVASLVASSAFAGAVTDPSSQDPFGRT
AASIASSCGHKGVAGYLSEVALTSHLSSLTLEENELSKGTADVEAERTISSISTTSAATHEDQLSLKDTLAAVRNAAQAAARIQSAF
RAHSFRKRRQREAARAATTSGDEYCVLSNDVLGLSAASKLAFRNMRDYNSAALAIQKKYRGWKCRKDFLAFRQKVVKIQAHV
RGYQVRKEYKVCWAVGILEKVVLRWRRRGVGLRGFRLEEEPIEESEDEDILKLFRKQKVDAAINEAVSRVLSMVDSPEARQQYH
RILEKYRQAKAELGVNSDTVSTAHGDISNSDI

>NtCAMTA18 XP_016507792.1

MAESGYNINDLVREGHFRWLRPAEVVFILQNHEDQQLANQPPQKPASGSMFLFNKRVLRYFRKDGHSWRKKKDGRTVGEAHER
LKVGNAEALNCYYAHGEKNPNFQRRSYWMLDPAYEHIVLVHYRDITEIAAFMSQSSPISSTFSLSPSLYSTQHPGFTVFGSESYQQ
YPNESSPGSGEVCSDAGINGKGMNISDITGRTEGVSSSPRVEISQALRKLEEQLSLNDDSLEQIDPLYSEIENSDDVENFVHDNNSL
VQIQHKSNNLLLQPHSGESSESQHQLLNLDGNIWKEMLDHCRSFPAAESPAKCFEKLDENGTLQTSSGVGPIEATESDRWLKFGG
KALKSSLTNFKQVEDFKYPACARINTYGSYSDQYTTIFDQDQIGTSFEDDMSLTIAQKQKFTIHDISPDWGYSSEATKIVIVGSFLC
NPSEYTWTCMFDDIEVPVQIINEGAIRCQAPPHLPCKVTLCVTTGNRVSCSEVWEFEYRVKFDDHGQKNLAEVGGACKSSEELL
LLVRFVQMLLSDSSVQKGDGSGSSNDILENSKASEDSWSQVIESLLFGTSTSMVTVDWLLQELLKDRLKQWLSSKLQVKNNQM
GYSFSRKEQGIIHMVAGLGFEWALHPILDAGVGVNFRDINGWTALHWAARFGREKMVASLVASSAFAGAVTDPSSQDPFGRTAA
SIASSCGHKGVAGYLSEVALTSHLSSLTLEENELSKGTADVEAERTISSISTTSAATHEDQLSLKDTLAAVRNAAQAAARIQSAFRA
HSFRKRRQREAARAATTSGDEYCVLSNDVLGLSAASKLAFRNMRDYNSAALAIQKKYRGWKCRKDFLAFRQKVVKIQAHVRG
YQVRKEYKVCWAVGILEKVVLRWRRRGVGLRGFRLEEEPIEESEDEDILKLFRKQKVDAAINEAVSRVLSMVDSPEARQQYHRI
LEKYRQAKAELGVNSDTVSTAHGDISNSDI

>NtCAMTA19 XP_016507793.1

MAESGYNINDLVREGHFRWLRPAEVVFILQNHEDQQLANQPPQKPASGSMFLFNKRVLRYFRKDGHSWRKKKDGRTVGEAHER
LKVGNAEALNCYYAHGEKNPNFQRRSYWMLDPESYQQYPNESSPGSGEVCSDAGINGKGMNISDITGRTEGVSSSPRVEISQAL
RKLEEQLSLNDDSLEQIDPLYSEIENSDDVENFVHDNNSLVQIQHKSNNLLLQPHSGESSESQHQLLNLDGNIWKEMLDHCRSFP
AAESPAKCFEKLDENGTLQTSSGVGPIEATESDRWLKFGGKALKSSLTNFKQVEDFKYPACARINTYGSYSDQYTTIFDQDQIGTS
FEDDMSLTIAQKQKFTIHDISPDWGYSSEATKIVIVGSFLCNPSEYTWTCMFDDIEVPVQIINEGAIRCQAPPHLPCKVTLCVTTGN
RVSCSEVWEFEYRVKFDDHGQKNLAEVGGACKSSEELLLLVRFVQMLLSDSSVQKGDGSGSSNDILENSKASEDSWSQVIESLL
FGTSTSMVTVDWLLQELLKDRLKQWLSSKLQVKNNQMGYSFSRKEQGIIHMVAGLGFEWALHPILDAGVGVNFRDINGWTAL
HWAARFGREKMVASLVASSAFAGAVTDPSSQDPFGRTAASIASSCGHKGVAGYLSEVALTSHLSSLTLEENELSKGTADVEAERTI
SSISTTSAATHEDQLSLKDTLAAVRNAAQAAARIQSAFRAHSFRKRRQREAARAATTSGDEYCVLSNDVLGLSAASKLAFRNMR
DYNSAALAIQKKYRGWKCRKDFLAFRQKVVKIQAHVRGYQVRKEYKVCWAVGILEKVVLRWRRRGVGLRGFRLEEEPIEESE
DEDILKLFRKQKVDAAINEAVSRVLSMVDSPEARQQYHRILEKYRQAKAELGVNSDTVSTAHGDISNSDI

>ZmCAMTA1 GRMZM2G171600

MQQQQQGLDIGKLQEVVKTRWLKPQEVLKILQNHELFTISHKPPQKPQSGSWFLFNRRVLRYFRNDGFEWQKKRNGKTINAHE

RLKVDNVDALNCYYARGDKNPTFQRRIYWMLDPAYEHIVLVHYRDVLEGSISVSARNDSSTLNQNGSASRAEVHSSPGWTSELI
AHCTNSCSPGSAEEVSSQISASESDLIQHKAALRKLKMQLSLEDKEDCDVNAEDVPADNEPIILPVIQNEEPGTSRNHDDIFDVLD
FSEDHINGTGTHPCHSAIDVLKNSDTWLEDDQLEAILHPACMTLTENQWFRIHEVSPESAFSYESTKVIIVGDFLCNPPHSSWQVL
FGDVKVCVEIIQQGVIRCHTPCLDAGKVRMCLLDGNGKSCSEAREFEFLEKPTKCMIDGNTNPCNEAQDVKLHQIPTKSSEELSL
LLHYVHTLFDGHASGLFSNFSLPLQNLGCGIQSNQMDVMKKAYKQLDPENVVSSVMEVLLNDKFKQWLSSKCEQNIDGDHLLP
KQYRNIIHTVAALGYDLALKPLLSSGVPINYRDANGWTALHWAARFGREDMVVALLTAGAAAGALSHPTSEDPAAKTPASIALA
YGFKGLSAFLSEAQLTTHLDSIESKENGKLDSREEGICRAVDRISDKSSHVHGGTDDQLALKDSLGAVRNAVQAAGRIQAAFRIFS
FKKKKEMALGNRNSCCLSISEAGAVSHDMLEKAVLSIQKNFRCWKKRKEFLKMRNNVVRIQARVRAHQERNKYKELISSVGILE
KVMIRWYHKGVGLRGFNSGAMTIDEEVDEDVAKVFRKLRVETAIDEAVSRVSCIIGSPKAMHQYRRMLNRYQQTKDDQEK
>ZmCAMTA2 GRMZM2G431243
MPRSGSLFLFDRKVLRYFRKDGHNWRKKKDGKTVKEAHEKLKAGSIDVLHCYYAHGEENENFQRRTYWLLEEDFTHIVLVHY
LEVKGCKQSFNRVKEELMQLSNVDSPSCSNSITSQNQMGPQNMEAAESPISGQISEYEDTESDNCRASSRYHPLAEMQQLVDGVI
TENMLYPSASTVGSRQGYHGEMLPITDNLDNRSFSNHDIARMLDGANIGLSDVSSTLFDSVPFNEPFTNYSAGFTEPTLHSSFACL
EANDLDDSSCLQTFTSEALYTNHLNQKEADALGFTGILASEVNRDRYNDGSIKHSLLKQSSLDLLTIEAPGLKKNDSFSRWMSKE
LEELDAYVVNPSLSQDQLFSILDVSPSCAYIGTNTKVSVTGTFLVNKKHVESHRWSCMFGDVEVPAEVLTDGTLRCYAPAHQSGR
VPFYVTCSNRVACSEVREFEYRDSEAHYMETSRSQANGVNEMHLHIRLEKLLTLGPDDHQMLVISSGNEKYEIMNAINSLMLDG
KWSNQESSVKEVVSTARVQSLKKLVKEKLHQWLICKVNDDGKGPNVLCKEGQGVIHLVAALGYDWAIRPIMIAGVNVNFRDAH
GWTALHWAASLGRERTVSVLIANGAAAGALTDPTSEFPSGRSPADLASVNGHKGIAGFLAESALTSHLSALTIRESNDSTVEACG
LPFAEDLTGIDSVHLAGEGPDAESLEGSLSAVRKSTQAAARIFQAFRVESFHRKKVVEYGDDTCGLSDECTLSLVSLKNVKPGQH
DTHLHSAAVRIQNKFRGWKGRKEFMIIRQRIVKLQAHVRGHQVRKNYRKVVWSVGIVEKVILRWRRKRPGLRGFRPEKQLEGP
SQIQPAKAEDEYDFLHDGRRQAEARLQRALARVHSMSQYPEAREQYHRLTTCVAEMKQSRMMQDEMLSQAAGGADDFMAGL
EDLICIDDAPPRCLPFGEAEPFYIR
>ZmCAMTA3 GRMZM2G447551
MAEARRHAVAPQLDIEQILKEAQHRWLRPAEICEILKNYRNFHIAPEPPNRPPSGSLFLFDRKVLRYFRKDGHSWRKKKDGKTVK
EAHERLKSGSVDVLHCYYAHGEGNENFQRRSYWMLEEDFMHIVLVHYLEVKGGKSTSRIRGHDDMLQAARTDSPLSQLPSQTT
EGGNSLSGQASEYEETESDIYSGGAGYHPFSWTQHHENGGGPVIGTSIPSSYVPALPLGNLQGFPATVTNTDIYSRQDALPVTLNE
PGLAIEFNGADNQLDPSSLNGLVKPFLQMSTPQSTVPSELFPFTEEHGNESFTFHEVYSNGLSIKDAEIVGTNEESVWKLPGAISSIP
SEDSFQQNDRSLEETISYPLLKTRSSNLSEMLKDSFKKSDSFTRWMSKALGEVDSQIKSSSGVYWNSEETNNIIETSSCDQLDQCTI
DPVLAQEQLFSIVDFSPSWTYAGSKTRVLINGKFLNSAELKRCKWSCMFGEVEVPAEISADGILRCYSPSHKPGRVPFYVTCTNRL
ACSEIREFEFRPSVTQYMDAPSPHGATNKTYLQMRLDNLLSLGHNEYQATVSNPTKEMVDLSKKISSLMTDNDSWSQLLKLASD
NEPVTDDDQDEFFEKHLKEKLHIWLVHKASDGGKGPNVLDDEGQGVLHLAAALGYDWVIRPAVSAGVNINFRDAHGWTALHW
AAFCGRERTVVALIALGAAPGALTDPTPIFPTGSTPADLASANGYKGISGFLAESSLTSHLQTLDLKEGKGSNPPEISGLPGIGDVTE
RRASPLAGEGLQAGSMGDSLGAIRNAAQAAARIYQVFRVQSFQRKQAVQYEDDNGAVSDDRAISLLSVKPSKPVQLDPLHAAA
TRIQNKYRGWKGRKEFLLIRQRIVKIQAHVRGHQVRKHYRKIIWSVGIVEKIILRWRRKGAGLRGFRSTEGATVGSSSNLIQNKPE
DDYDFLQQGRKQTEERLQKALARVKSMAQYPDARDQYQRILTVVTKIQESKAMQEKMLEESTEMDEGFFMSDFKELWDDDIP
MPSWS
>ZmCAMTA4 GRMZM2G143205
MSQSFDINVLREEARSRWLKPSEVYYILQNHERFPITHEAPKKPPSGSLFLYNRRVNRYFRRDGHTWRRKKDGRTVGEAHERLK
VGNVDSLSCYYAHGEQNPCFQRRCFWMLEPAYEHIVLVQYREVAEGRYYSSQLSNGPPEPLSSLGYPNAICGNQYHRSTSGTSEG
SESHQSYSNLSSVTEVSSYSGNKEYNKNDGSLLSIPEVGHTCQQNQTGNGNSKNKSELNMALKKIAEQLSLGEDDDDDYIYSNQ
THSMGGDNQIKQIRQEGTQKGLSRNIAPSWEDVLHSSSGLPTSSIYQQSDVKYQKKSEYQPPEILDSSDLRIQLSATKRFLLGPEAs
IDsPSLNSVLRNRVNsVTDTISAYDSRFESSLNPDWQTKTALTFQSNSQGSEITELFDHDHFEPYSREDTTISLGQTNKFNIREVSPE
WAFSYEITKVIITGDFLCDPSNLCWAVMFGDNEVPVEIVQPGVLRCHTPLHSNGNLRICITSGNREVCSEFKDFEFRSKPTSSSFTDI
APSSRHLKSSEELLLLAKFARMLLSGNGNREVPDGDPQSGQCPKLKTNEELWDRLINELKVGCENPLSSVDWIVEQLLKSNLQQ

WLSVKLRGFNGTDFLSKQEQGIIHLISALGYEWALSPVLSAGVGLNFRDSNGWTALHWAAYFGREKMVAALLAAGASATAVTD
PTAQDPVGKTAAFLASERGHTGLAGYLSEVSLTSYLASLTIEESDVSKGSAEVEAERAVEGISQRNAQRHGGTEDELSMKDSLAA
VRNAAQAAARIQNAFRAFSFRKRQQKTARLRDVYGMTQEDIDELAAASRLYHQAHASSGQFYDRAAVSIQKKYKGWKGRKHF
LNMRRNAVKIQAHVRGHQVRKKYRTIVSTVSVLEKVILRWRRKGHGLRGFRAEQQPMVEAIEEDDEEDDDFDDDEAVKVFRRQ
KVDQAVKEAVSRVLSMVDSTEARMQYRRMLEEFRQATAELEGSNEVTSIFDSDLELLGINNFM

>ZmCAMTA5 GRMZM2G152661

MGFNRILTNGAPIGLFFGGSLFLYNRRVNRYFRRDGHTWRRKKDGRTVGEAHERLKFFVHVFLQTWSKHSHRKQGRCRIPWPRV
IVPNCGSGSPWVIVPNARESLLQHSHDARTCVSVGQVEFHGCFRLSQMNAYYGALGAFGQVGNVDALSCYYAHGEQNPSFQRR
CFWMLEPAYEHIVLVQYREVDVVRNYSSQLSNGLPEPLSSLGYPNAICGNQYHSSSSGASECSESHQSYSNLSSVTEVSSYSGNK
EYTKNDGGLLSIPEVGHTCLQQNRTDNGNSKNKSGLNIALKKIAEQLSLGEDDDDYIFSNQAHSVGGDNQVKHIQQEGTQKGLS
RNIAPSWEDVLQSSSGLPISSIYQQSDVEYQKNSEYHPPERLDSSDLRIQLAAAKKFLLGPEATVDsPSLNSVLRNRANCVTDTISA
YDSRFGSSLNPDWQTKTALTFQSNSQGSEITELFDHGHFEHYSREDTTFSLGQTNKFNIREISPEWAFSYEITKVIITGDFLCNPSNL
GWAVMFGDSEVPAKVVQPGVLLCHTPLHCSGNLRICITSGNREVCSEFKDFEFRSKPSSSFTDIAPSSRHLKSSEELLILAKFARML
LSGNGNPEVPDGDPQSGQCPKLKMDEGLWDRLIEELKVGCESPLSSVDWILEELLKSKLQKWLSVKLRGFNGTDSISKHDQGIIH
LISALGYEWALSSVLSVGVGLNFRDSNGWTALHWAAYFGSCSFRRNTKVAKVATFLNMRRNAVKIQAHVRGHQVRKKYRTIVS
TVSVLEKVILRWRRKGHGLRGFRAEQQSMVEAIEEDDEEDDDFDDDEAVKIFRRQKVDQAVKEAVSRVLSMVDSTEARMQYRR
MLEEFRQATNWKDQMK

>ZmCAMTA6 GRMZM2G032336

MAGGAGGRDPLVASEIHGFLTCADLNFDKLMMEAGTRWFRPNEIYAVLANYARFKVHAQPIDKPISGTVVLYDRKVVRNFRKD
GHNWKKKKDGKTVQEAHEKLKIGNEEKVHVYYARGEDDPNFFRRCYWLLDKELERIVLVHYRQTSEESALPPSHVEAEVAEVP
RINMIHYTSSTDSASAHTELSSSAAAAPEDINSNGGGAVSSETDNQGSSLESFWVDLLESSMKNDTPVDASACGGSLVSNQQTNN
GMGDSGNNILYINATSNAIFSPPTNVVSEAYANPGLSQVSESYFGSLKDQANHAPSLLTSDLDSQSKQHTNSLMKTPVSDNmPND
VPARQNsLGLWKYLDDDISLDDNPSSGILPTEQVTGEIPFQITEISSEWAYCTEDTKVLVVGCFHENYRHLAGTNLFCVIGDQCVD
ANIVQTGVYRFIARPHAPGRVNLYLTLDGKTPISEVLSFHYRmVPDsQNLAEDEPQKsKLQMQMRLARLLFTTNKKKIAPKLLVE
GSKVSNLLSASTEKEWMDLSKFVTDSKGTYVPATEGLLELVLRNRLQEWLVEKLIEGHKSTGRDDLGQGPIHLCSCLGYTWAIH
LFSLSGFSLDFRDSSGWTALHWAAYCGREKMVAALLSAGANPSLVTDPTHDVPGGQTAGDLAAGQGYHGLAAYLSEKGLTAHF
EAMSLSKGKRSTSRTESLKRNTKEFENLSEQELCLRESLAAYRNAADAASNIQAALRERTLKLQTKAIQLANPENDASAIVAAMR
IQHAYRNYNRKKMMRAAARIQSHFRTWQIRRNFMNMRRQAIKIQAAYRGHQVRRQYRKVLWSVGVVEKAILRWRKKRKGLR
GIATGMPVAMATDAEAASTAEEDYYQVGRQQAEDRFNRSVVRVQALFRSHRAQQEYRRMKVAHEEAKVEFGRK

>ZmCAMTA7 GRMZM2G017368

MDAESPISGQISEYEDAETDNSRASSRYHPFTEMQQPVDGTVMGNFFGASSPSVSVNNLAAGYLGEMQPTGANFTSHFATRNDI
ASVFNDTGsELGGGPKTSIDSVLLGEPFPEYPGGFMESTLYSSVATLGNSLEDGLQTFMSEALYTNNLTQKEVDALGAAGITSSKY
LKNDAVGHWSLPISMSWYDVTLYFQTENDGYTDQSVRYPLLKQSSSDLFKMEPDGLKKFDsFSRWMsNELPEVVDLDIKSSSDA
FWSTTETVNVADGSSIPINEPLDVFVVSPSLSQDQLFSIIDVSPSWAYNGTKTKVLITGTFLAKKEDVENCSWSCMFGDSEVSAEV
LVDGSLRCYTPVHHSGRVPFYVTCSNRVACSEVREFEFRDSETHYMDISDKHTTGINEMHLRIRLDKLLSLEPEDYEKYVLSNGN
KSELINTISSLMLDNNLSNLALPSDEKELCTVQDQNLEKQVKEKLYYWLIHKIHDDGKGPNVLGKEGQGAIHLVAALGYDWAIK
PIVAAGVNINFRDIRGWTALHWAACCGRERTVGALIASGAASGALTDPTQQYPSGRTPADLASENGHKGIAGFLAESALTSHLSAL
TLKESPSGNVEEICGLTAAEGFAASSSSQLACVNSQEESLKDSLGAVRKSTQAAARIFQAFRVESFHRKKVIEYGDDDCGLSDERT
LSLVSLRNPKSGHGDSHSAAVRIQNKFRGWKGRKEFMLIRQKIVKIQAHVRGHQVRKNYRKVVWSVGIVEKVILRWRRKGRGL
RGFQPEKQLEGPSWQIQPAKAEAEDEYDFLKDGRKQATGRLDRALARVRsmNQYPEARDQYRRLQACVNSLRESQAMQDRML
ADSAGTDGGDFMTELEELCRDDGDAPMSTIS

>ZmCAMTA8 GRMZM2G153594

MLEEDYMHIVLVHYLETKGGKSSRARGNNIIQEAAVGSPSQIMEVESSLSGQASEYEEAESDIYSGGAGYDSFTWMQQHENGTG
PVIDSSLFSSYTPASSIGNYQGQHATQNKSFYPVNQHNGPLILNGSSDMLGTNGRANQTDLPSWNSVIELDEPGQMPHLQFPVPS

DQGATTEGLGVDYLTFDEVYSDGLSLNDIGAAGTHGKSYLQFSSATGDLSATENSLPQQNDGSLEEAAIGYPFLKTQssNLsDILK DsFKKTDSFTRWMSKELPEVEDSQIHSSSGGFWSTGEANDIIEASSHEPLDQFTVSPMLSQEQLFSIVDFAPNWTYVGSKTKILVAG NILNDSQITERCKWSCMFGEVEVPAKILADGTLICYSPQHKLGRVPFYITCSNRLACSEVREFEFRPTVSQYMDAPSPHGETNKVY FQIRLDKLLSLEPDEYQATVSNPSLEMIDLSKKISSLMASNDEWSNLLKLAVDNEPSTADHHDQFVEKLIKEKLHVWLLNKVGM GGKGPSVLDDEGQGVLHLAAALGYDWAIRPTLAAGVNINFRDVHGWTALHWAAICGRERTVVALIALGAAPGALTDPTPDFPG STPADIASANGQKGISGFLAESSLTSHLQALNLKEANMAQISGLPGIGDVTERDSLHPPSGDSLGPVRNAAQAAARIYQVFRVQSF QRKQAAQSEDDKGGMSDERALSLLSVKPPKSGQLDPLHSAATRIQNKFRGWKGRKEFLLIRQRIVKIQAHVRGQQVRKHYRKI VWSVGIVEKVILRWRRRGAGLRGFRSTEGSVESSNGGTSSSSIQDKPSGDDYDFLQEGRKQTEERLQKALARVKsmAQYPEARD QYHRILTVVSKMQESQAMEEKMLEESAGMDFMSEFKELWDDDTPIPGYI

>ZmCAMTA9 GRMZM2G341747

MASAEARRLAVVPQLDIEQILKEAQHRWLRPAEICEILKNYRNFRIAPEPPNRPPSGSLFLFDRKVLRYFRKDGHNWRKKNDQKT VKEAHERLKSGSIDVLHCYYAHGEENINFQRRTYWMLEEDYMHIVLVHYLETKGGKSSRARGNNMIQEAAVDSPLSQLPSQTM EGESSLSGQASEYEEAESDIYSGGAGHDSFTWVQQHENGTGPMIASSVFSSYTPALSIGNYHGLHATQNTSFYPVNQLNSPVILNG SSAMLGTNGCANQTDLPSWNSVIELDHEPVQMPDLQFPVPPDQGTSTEGLGVDYLTFDEVYSDGLSLQDIGATGTHGESYLQFS SGTGDLAATVNSFPQENDGSLEAAIGYPFLKTQssNLsDILKDsFKKTDSFTRWMSKELPEVEDSQIQSSSGAFWSSEEANNIIEASN HEALDQFTVSPMLSQDQLFSIVDFSPNWTYVGSKTKILVAGNILNDSQITERSKWSCMFGEVEVPANILADGTLICYSPQHKPGRV PFYITCSNRLACSEVREFEFRPTVSQYMDAPSPHGETNKVYFQIRLDKLLSLGPDEYQATVSNPTLEMVDLSRKISSLMASNDEW SNLLKLAVDNEPSTADQQDQFAENLIKGKLHIWLLNKVGMGGKGPSVLDDEGQGVLHLAAALGYDWAIRPTLAAGVNINFRDI HGWTALHWAAFCGRESTVVALIALGAAPGALTDPTPDFPGSTPADLASSNGQKGISGFLAECSLTSHLQVLNLKEANMAQISGLP GIGDVTERDSLQPPSGDSLGPVRNATQAAARIYQVFRVQSFQRKQAAQYEDKGGMSDERALSLLSVKPPKSGQLDPLHSAATRI QNKFRGWKGRKEFLLIRQRIVKIQAHVRGHQVRKHYRKIVWSVGIVEKVILRWRRRGAGLRGFRSQEGSVESSSGGTSSSSIQN KSSGDDYDFLQEGRKQTEERLQKALARVKsmAQYPEARDQYQRIFTVVSKMQESQAMQEKMPEESAEMDMSEFKELWDDDAP IPGYF

Table S2. Primers for qRT-PCR validation of SmCAMTAs gene, F: forward primer, R: reverse primer

Gene name	Sequence of primer (5’-3’)
SmCAMTA1-F	ACAACTCACTGGGAAGGAAAT
SmCAMTA1-R	GAGCATCTTACGGTCAAACAG
SmCAMTA2-F	GGACAGTTCGGCGTCTACAAA
SmCAMTA2-R	ATCGGATGATAAGGGACAGCA
SmCAMTA3-F	AGCCCTGAACTGTTATTATGC
SmCAMTA3-R	GAGCCCGTATGTTGAGTAGAA
SmCAMTA4-F	AGACATCACCAGGAGGACAGA
SmCAMTA4-R	TGAGGCAGCAACAGAAGATTA
SmCAMTA5-F	GCATTATTTCGGTGGCGTTTG
SmCAMTA5-R	CAGCGTTGCTTTATTATGTTCCA
SmCAMTA6-F	GAAGTAGAGGTTCCCGCAGAG
SmCAMTA6-R	AACAGGTCCCAAGGACAATAA

Table S3. Primers for PCR of SmCAMTA and SmCBF genes, F: forward primer, R: reverse primer

Gene name	Sequence of primer (5’-3’)
SmCAMTA1-F	ACAACTCACTGGGAAGGAAAT
SmCAMTA1-R	GAGCATCTTACGGTCAAACAG
SmCAMTA2-F	GGACAGTTCGGCGTCTACAAA

SmCAMTA2-R	ATCGGATGATAAGGGACAGCA
SmCAMTA3-F	AGCCCTGAACTGTTATTATGC
SmCAMTA3-R	GAGCCCGTATGTTGAGTAGAA
SmCAMTA4-F	AGACATCACCAGGAGGACAGA
SmCAMTA4-R	TGAGGCAGCAACAGAAGATTA
SmCAMTA5-F	GCATTATTTCGGTGGCGTTTG
SmCAMTA5-R	CAGCGTTGCTTTATTATGTTCCA
SmCAMTA6-F	GAAGTAGAGGTTCCCGCAGAG
SmCAMTA6-R	AACAGGTCCCAAGGACAATAA
SmCBF1-F	ATTCATGATCACCGCGGTGGGGA
SmCBF1-R	TCCGAAGAGGATGGCACGGCCTG
SmCBF2-F	ATTCTGCCGAGGAGCACATGGAGG
SmCBF2-R	TCCCTACTCATTGCTTGCAGGG
SmCBF3-F	ATTCATGATCACCGCGGTGGGG
SmCBF3-R	TCCACCTTGCGCGTGCTACCG
SmERF1-F	ATCCGAGATTTGATACAG
SmERF1-R	TGAACGAAAGCTCCCTTA

Table S4. Annotation information of other genes in the sub network of SmCAMTA2.

Gene ID	Annotation
EGP33830	Pyruvate dehydrogenase E1 component subunit alpha-3, chloroplastic OS=Arabidopsis thaliana OX=3702 GN=PDH-E1 ALPHA PE=2 SV=1
EGP19277	Secretory carrier-associated membrane protein 4 OS=Arabidopsis thaliana OX=3702 GN=SCAMP4 PE=1 SV=1
EGP13905	Uncharacterized protein At2g34160 OS=Arabidopsis thaliana OX=3702 GN=At2g34160 PE=1 SV=1
EGP28770	F-box/kelch-repeat protein At3g23880 OS=Arabidopsis thaliana OX=3702 GN=At3g23880 PE=2 SV=1
EGP32522	Ubiquitin-conjugating enzyme E2 20 OS=Arabidopsis thaliana OX=3702 GN=UBC20 PE=2 SV=1
EGP07338	Sm-like protein LSM3A OS=Arabidopsis thaliana OX=3702 GN=LSM3A PE=1 SV=1
EGP18038	Plasma membrane ATPase 2 (Fragment) OS=Solanum lycopersicum OX=4081 GN=LHA2 PE=3 SV=1
EGP10537	Pentatricopeptide repeat-containing protein At2g37230 OS=Arabidopsis thaliana OX=3702 GN=At2g37230 PE=2 SV=1
EGP10060	Ubiquitin receptor RAD23b OS=Arabidopsis

	thaliana OX=3702 GN=RAD23B PE=1 SV=3
EGP08960	Cytochrome P450 82A3 OS=Glycine max OX=3847 GN=CYP82A3 PE=2 SV=1
EGP16737	BTB/POZ and MATH domain-containing protein 2 OS=Arabidopsis thaliana OX=3702 GN=BPM2 PE=1 SV=1
EGP31613	Cullin-3A OS=Arabidopsis thaliana OX=3702 GN=CUL3A PE=1 SV=1
EGP00685	Nucleolar GTP-binding protein 1 OS=Arabidopsis thaliana OX=3702 GN=At1g50920 PE=2 SV=1
EGP27228	Polyadenylate-binding protein 5 OS=Arabidopsis thaliana OX=3702 GN=PAB5 PE=1 SV=3
EGP05651	Acyl-CoA-binding domain-containing protein 4 OS=Arabidopsis thaliana OX=3702 GN=ACBP4 PE=1 SV=1
EGP26096	AT-rich interactive domain-containing protein 5 OS=Arabidopsis thaliana OX=3702 GN=ARID5 PE=1 SV=1
EGP13931	Probable RNA methyltransferase At5g51130 OS=Arabidopsis thaliana OX=3702 GN=At5g51130 PE=2 SV=1
EGP12619	Probable eukaryotic translation initiation factor 5-2 OS=Arabidopsis thaliana OX=3702 GN=At1g77840 PE=1 SV=1
EGP25703	E3 ubiquitin-protein ligase SPL2 OS=Arabidopsis thaliana OX=3702 GN=SPL2 PE=2 SV=1
EGP15964	BRCT domain-containing protein At4g02110 OS=Arabidopsis thaliana OX=3702 GN=At4g02110 PE=4 SV=3
EGP15132	Putative ABC1 protein At2g40090 OS=Arabidopsis thaliana OX=3702 GN=At2g40090 PE=2 SV=2
EGP05619	Mitogen-activated protein kinase kinase kinase NPK1 OS=Nicotiana tabacum OX=4097 GN=NPK1 PE=1 SV=1
EGP06516	Protein BASIC PENTACYSTEINE4 OS=Arabidopsis thaliana OX=3702 GN=BPC4 PE=1 SV=1
